# Diagnostic performance of plasma pTau217 in genetically admixed South American populations

**DOI:** 10.1038/s41467-026-76434-2

**Published:** 2026-08-06

**Authors:** Pamela V. Martino-Adami, Joice Coutinho de Alvarenga, Pilar Freccero, Hanna Huber, Julio Fernandez, Ivonne Carolina Bolaños Burgos, Maria Barbara Postillone, Gabriela Tomé Oliveira Engelmann, Ines Mintz, Marco Aurelio Romano Silva, Nancy Medel, Erika de Oliveira Hansen, Julieta Lisso, Natalia Dias Silva, Andréa Teixeira Carvalho, Nicolás Irureta, Débora Marques de Miranda, Mariana Vallejo-Azar, Luiz Armando Cunha de Marco, Stefanie Heilmann-Heimbach, Jonas Jardim de Paula, Silvia Kochen, Rafaela Teixeira de Ávila, Patricia Solis, Anja Schneider, Paula Gonzalez, Bernardo de Mattos Viana, Maria Carolina Dalmasso, Maria Aparecida Camargos Bicalho, Alfredo Ramírez

**Affiliations:** 1 Division of Neurogenetics and Molecular Psychiatry, Department of Psychiatry and Psychotherapy, Faculty of Medicine and University Hospital Cologne, University of Cologne, Cologne, Germany; 2 Reference Center for the Elderly, University Hospital of the Universidade Federal de Minas Gerais (UFMG), Belo Horizonte, Brazil; 3 Molecular Medicine Postgraduate Program, Faculty of Medicine, Universidade Federal de Minas Gerais (UFMG), Belo Horizonte, Brazil; 4 Older Adult Psychiatry and Psychology Extension Program (PROEPSI), Faculty of Medicine, Universidade Federal de Minas Gerais (UFMG), Belo Horizonte, Brazil; 5 Studies in Neuroscience and Complex Systems Unit (ENyS-CONICET-HEC-UNAJ), Florencio Varela, Argentina; 6 German Center for Neurodegenerative Diseases (DZNE), Bonn, Germany; 7 Asistencia Medica Integral, Hospital El Cruce, Florencio Varela, Argentina; 8 Neurotec R National Institute of Science and Technology (INCT-Neurotec R), Faculty of Medicine, Universidade Federal de Minas Gerais (UFMG), Belo Horizonte, Brazil; 9 Fundação Oswaldo Cruz (Fiocruz), Instituto René Rachou, Minas Gerais, Brasil; 10 Institute of Human Genetics, University Hospital of Bonn & University of Bonn, Bonn, Germany; 11 Department of Old Age Psychiatry and Cognitive Disorders, University Hospital Bonn, Bonn, Germany; 12 Cologne Excellence Cluster on Cellular Stress Responses in Aging-Associated Diseases (CECAD), University of Cologne, Cologne, Germany; 13 Department of Psychiatry & Glenn Biggs Institute for Alzheimer’s and Neurodegenerative Diseases, San Antonio, TX USA

**Keywords:** Alzheimer's disease, Diagnostic markers

## Abstract

Plasma pTau217 is a leading biomarker for Alzheimer’s disease, but evidence from genetically admixed populations in low- and middle-income countries remains limited. We evaluated the diagnostic performance of pTau217 and pTau217/Aβ42 measured using Simoa technology in memory-clinic cohorts from Brazil (Cog-Aging-Study, *n* = 353) and Argentina (GeNED.ar, *n* = 134). Here we show that both biomarkers accurately identified cerebrospinal fluid (CSF)-defined amyloid pathology in Cog-Aging-Study-Brazil and showed high concordance with clinical diagnosis across both cohorts. Classification performance was improved using two-cut-off approaches that account for diagnostic uncertainty. We further show that *APOE-ε4*, lower body mass index, and reduced kidney function were associated with higher pTau217 in Cog-Aging-Study-Brazil, whereas education also influenced biomarker levels in GeNED.ar-Argentina. African ancestry modified *APOE-ε4* effect on CSF but not plasma biomarkers. These findings support the use of plasma pTau217-based biomarkers in genetically admixed South American populations and in settings with limited access to CSF testing and brain imaging.

## Abstract (web summary)

Martino-Adami et al. show that plasma pTau217 accurately identifies Alzheimer’s disease pathology and aligns with clinical diagnosis in admixed South American cohorts, supporting its use where access to cerebrospinal fluid testing is limited.

## Introduction

Dementia is one of the leading causes of disability and death among older adults worldwide. The number of people living with dementia is projected to rise substantially in the coming decades, especially in low- and middle-income countries (LMICs). Alzheimer’s disease (AD) accounts for 60% to 80% of all dementia cases and begins years before the onset of clinical symptoms, offering a window for early intervention. Early detection is crucial, especially as disease-modifying treatments become available. However, in high-income countries (HICs), even dementia specialists misdiagnose AD in 25-30% of cases based solely on clinical evaluation, limiting treatment options^1^. Similar challenges are expected in LMICs, where access to confirmatory testing remains limited.

Current AD biomarkers capture the core histopathological features of the disease, including amyloid pathology (measured by CSF amyloid-β [Aβ] or PET), neurodegeneration (indicated by CSF total Tau), and tangle pathology (assessed by Tau-PET or CSF phosphorylated Tau [pTau])^2^. However, access to these methods remains limited in many LMICs. In this context, blood-based biomarkers (BBMs) have emerged as a promising alternative, providing a less invasive, more affordable, and more accessible diagnostic option^3^.

Among BBMs, plasma pTau217 has emerged as the most reliable biomarker, demonstrating diagnostic accuracy and disease specificity comparable to CSF and PET measures^4^, and consistently outperforming plasma Aβ42/Aβ40 in detecting Aβ pathology^5^. Incorporating plasma pTau217 into the diagnostic process has been shown to significantly improve accuracy and reduce misdiagnosis^6^. When both plasma and CSF biomarkers are available, pTau217 can also serve as an objective measure to quantify the degree to which clinical diagnoses accurately reflect underlying AD pathology. Nevertheless, most evidence originates from HICs, with limited data from LMICs. In South America, where populations exhibit significant genetic and environmental diversity, the diagnostic performance of plasma pTau217 remains largely uncharacterised. Applying findings from HIC cohorts in North America or Europe may overlook the influence of genetic admixture and regional factors on biomarker performance.

Here, we assess the agreement between clinical diagnosis and CSF-defined AD pathology, as well as the diagnostic accuracy of plasma pTau217 and pTau217/Aβ42, in two genetically diverse South American cohorts: Cog-Aging-Study in Brazil and GeNED.ar in Argentina. We show that plasma pTau217-based biomarkers accurately identify AD pathology and exhibit high concordance with clinical diagnosis in these admixed populations. We further show that demographic, genetic, and clinical factors influence plasma biomarker levels, highlighting the importance of considering clinical context when interpreting biomarker results. Together, these findings support the implementation of plasma pTau217-based biomarkers in South American memory clinics and provide a foundation for the development of region-specific diagnostic methods.

## Results

### Participant characteristics

Cut-offs for CSF biomarkers were first derived in Cog-Aging-Study–Brazil, and plasma biomarker cut-offs were then derived independently and subsequently validated against CSF-based classifications within the same cohort. In GeNED.ar–Argentina, plasma cut-offs were also derived independently; however, no CSF data were available for validation. We next describe the clinical and demographic characteristics of each cohort.

In Cog-Aging-Study–Brazil, a total of 353 participants were included (Table 1). Of these, 114 had CSF samples and 304 plasma samples. However, 49 had only CSF samples available, 239 had only plasma samples, 45 had paired CSF and plasma samples collected within 12 months, and 20 had unpaired CSF and plasma samples collected more than 12 months apart. Differences between groups partly reflect distinct recruitment pathways, as described in the Supplementary Methods. The average age across groups ranged from 72.8 years in the CSF-only group to 77 years in the plasma-only group. Women made up between 51.1 and 71.1% of each group. *APOE-*ε4 carriers were more common among participants with paired samples (44.4%) and unpaired samples (45.0%) than among those with plasma only (28.8%). Years of education were generally low, with means from 4.4 to 6.5. Clinical diagnoses varied across groups. Among those with CSF only, 43.5% were cognitively unimpaired (CU), 21.7% had mild cognitive impairment (MCI), and 34.8% had dementia of the Alzheimer’s type (DAT). In the plasma-only group, 34.7% were CU, 45.6% MCI, and 19.7% DAT. For participants with paired CSF and plasma, at the time of CSF collection, 13.3% were CU, 26.7% MCI, and 60% DAT, whereas at plasma collection, 15.6% were CU, 35.6% MCI, and 48.9% DAT. Among those with unpaired samples, 10% were classified as CU, 70% as MCI, and 20% as DAT at both collection times. Across groups, BMI and kidney function were similar. A high prevalence of vascular and metabolic comorbidities was observed, including hypertension (62.2–95.0%), dyslipidaemia (50.0–80.0%), and diabetes (27.8–35.6%). The history of smoking ranged from 27.3 to 41.2%, and depression was reported by 44.2 to 60.3% of participants.

**Table 1:** Clinical and demographic characteristics of the participants from Cog-Aging-Study–Brazil and GeNED.ar–Argentina

	Cog-Aging-Study–Brazil						GeNED.ar–Argentina
Characteristics	CSF only (n = 49)	Plasma only (n = 239)	CSF and plasma (paired^a^, n = 45)	CSF and plasma (unpaired^b^, n = 20)	All CSF^e^ (n = 114)	All plasma^f^ (n = 304)	Plasma only (n = 134)
Age (mean (SD))	72.8 (8.6)	77 (7.1)	75.4 (7.4)^c^/75.5 (7.3)^d^	76 (6)^c^/75.7 (5.2)^d^	74.6 (7.7)	76.7 (7)	72 (6.8)
Sex (female, %)	69.4	71.1	51.1	65	61.4	67.8	70.1
*APOE* (%)							
ε2/ε2	-	0.4	-	-	-	0.3	0.8
ε2/ε3	5.6	10.3	2.2	-	3.0	8.4	9.3
ε2/ε4	2.8	0.9	2.2	10.0	4.0	1.7	2.3
ε3/ε3	55.6	59.9	53.3	60.0	55.4	58.9	61.2
ε3/ε4	30.6	25.4	35.6	25.0	31.7	26.9	23.3
ε4/ε4	5.6	3.0	6.7	5.0	5.9	3.7	3.1
Education (years, mean (SD))	5.4 (3.9)	6 (5.1)	6.5 (5.2)	4.4 (2.8)	5.7 (4.4)	5.9 (5)	8.7 (4.5)
Clinical diagnosis (%)							
CU	43.5	34.7	13.3^c^/15.6^d^	10^c^/10^d^	25.2	30.3	60.5
MCI	21.7	45.6	26.7^c^/35.6^d^	70^c^/70^d^	32.4	45.7	26.1
DAT	34.8	19.7	60^c^/48.9^d^	20^c^/20^d^	42.3	24.0	13.4
BMI (mean, (SD))	26.8 (4.4)	27.1 (4.5)	26.5 (4.9)	27.3 (3.3)	26.8 (4.4)	27 (4.5)	28.9 (5.2)
Smoking (%)	41.2	30.5	27.3	40	34.7	30.7	16.3
Alcoholism (%)	28.6	40.7	52.3	55	44.4	43.3	N/A
Hypertension (%)	69.4	74.1	62.2	95	71.3	73.7	68.1
eGFR (mean, (SD))	70.1 (18.5)	68.6 (16.9)	71.1 (17.7)	65.6 (14)	69.5 (16.9)	68.7 (16.8)	N/A
Dyslipidaemia (%)	50	58	55.6	80	58.4	59.1	37.5
Diabetes (%)	27.8	35.1	35.6	35	32.7	35.2	35.1
Depression (%)	47.2	60.3	44.2	47.4	45.9	57.1	16.3

^a^Samples collected within a 12-month interval.

^b^Samples collected more than 12 months apart.

^c^At CSF collection time.

^d^At plasma collection time.

^e^All patients with CSF samples.

^f^All patients with plasma samples.

*SD* standard deviation, *CU* cognitively unimpaired, *MCI* mild cognitive impairment, *DAT* dementia of the Alzheimer’s type, *BMI* body mass index, *eGFR* estimated glomerular filtration rate, *N/A* not available.

In GeNED.ar–Argentina, 134 participants with plasma samples were included in the study (Table 1). The mean age was 71.7 years, and 70.1% were women. *APOE-*ε4 carriers were less frequent (27.9%) than in Cog-Aging-Study–Brazil. Participants had, on average, higher educational attainment (8.7 years). Most individuals were CU (60.5%), followed by those with MCI (26.1%) and DAT (13.4%). The mean BMI was 28.9, and the prevalence of diabetes was 35.1%, similar to that in Cog-Aging-Study–Brazil. Hypertension was reported by 68.1% of participants, dyslipidaemia by 37.5%, smoking by 16.3%, and depression by 16.3%, all less frequent than in Cog-Aging-Study–Brazil. Information on alcoholism and kidney function was not available.

### CSF biomarkers in Cog-Aging-Study–Brazil

Given the limited availability of CSF biomarker data from Latin American populations, we first performed descriptive analyses assessing their distributional properties, sex differences, age-related associations, and comparison across clinical diagnostic groups.

The distributions of the available CSF Aβ42/Aβ40 ratio and pTau181 in this cohort were assessed by visual inspection of histograms and Q-Q plots. The Aβ42/Aβ40 ratio was approximately symmetric on the original scale, and log-transformation did not materially alter its distribution (Supplementary Fig. 1A, C); therefore, subsequent analyses were performed using untransformed values. In contrast, pTau181 was right-skewed on the original scale, and log-transformation substantially reduced the skewness, yielding an approximately symmetric distribution suitable for subsequent analyses (Supplementary Fig. 1B, D).

No significant associations were observed between either biomarker and sex (Supplementary Fig. 2A, B) or with age, either in the overall sample or within clinical diagnostic groups (CU, MCI, DAT) (Supplementary Fig. 2C, D). In contrast, both biomarkers were significantly associated with clinical diagnosis, with lower Aβ42/Aβ40 ratio and higher pTau181 levels observed in more advanced disease stages (Supplementary Fig. 2E, F).

### CSF Aβ42/Aβ40 and pTau181 cut-offs and diagnostic concordance in Cog-Aging-Study–Brazil

**Fig. 1:**
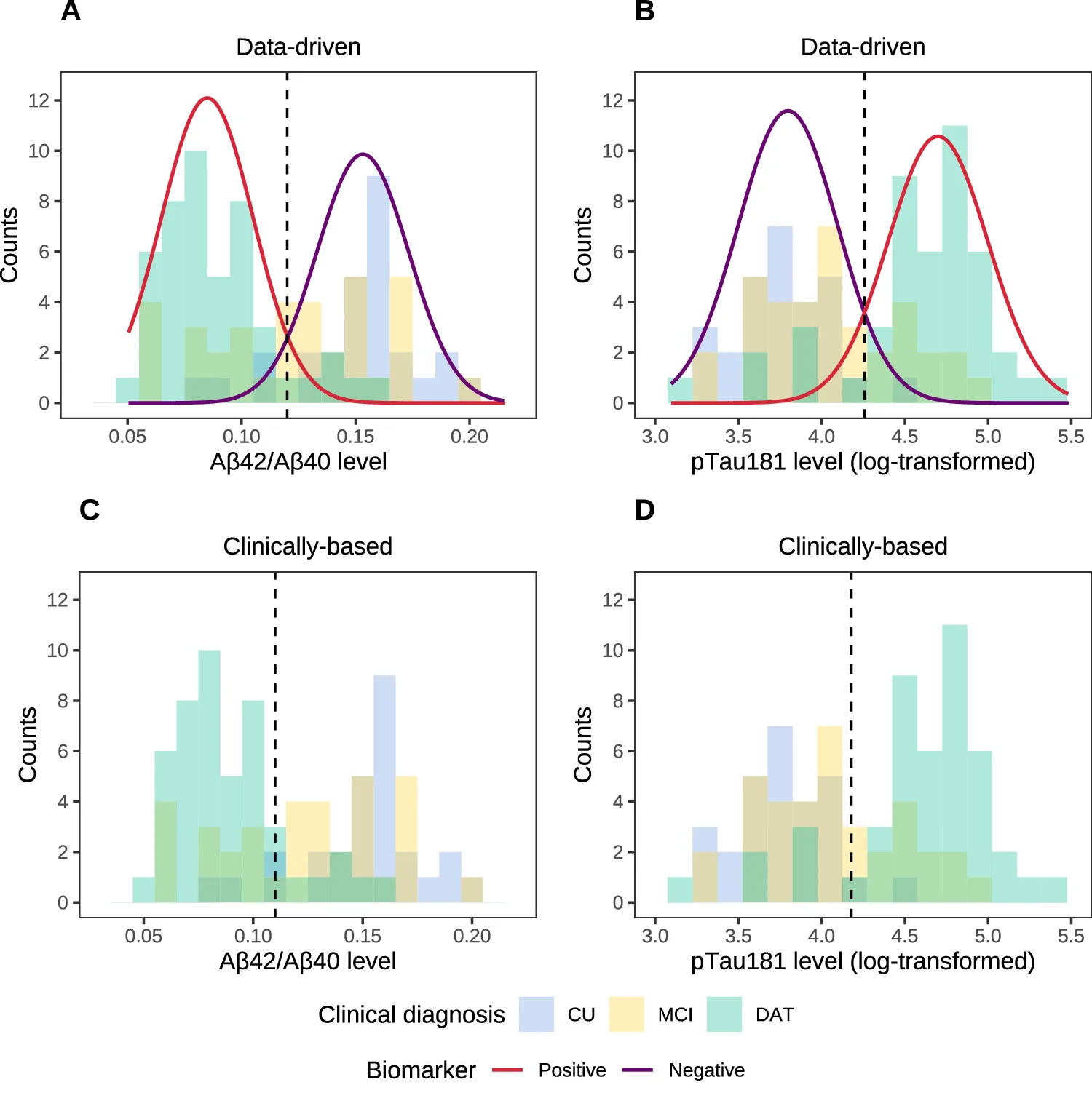
Data-driven and clinically-based cut-offs for CSF Aβ42/Aβ40 and pTau181 in Cog-Aging-Study–Brazil. Histograms show the distributions of biomarker levels across clinical diagnostic groups. Vertical dashed lines represent either data-driven (**A**,**B**) or clinically-based (**C**,**D**) cut-offs. For Aβ42/Aβ40, values to the left of the cut-off indicate abnormal levels; for pTau181, values to the right indicate abnormal levels. In the panels with data-driven derived cut-offs (**A**,**B**), the overlaid curves depict the modelled distributions of normal and abnormal biomarker values. CU, cognitively unimpaired; MCI, mild cognitive impairment; DAT, dementia of the Alzheimer’s type.

To derive cohort-specific CSF cut-offs, we employed two methods and compared their performance. First, we used an unbiased, data-driven approach. We applied Gaussian mixture modelling (GMM) to the full sample with available CSF data (CU, *n* = 28; MCI, *n* = 36; DAT, *n* = 47). By identifying natural groupings in biomarker distributions without relying on predefined clinical labels, this approach reduces potential bias from diagnostic misclassification^7^. For both biomarkers, a bimodal distribution with equal variances provided the best fit (Supplementary Table 1). These models yielded cut-offs of 0.12 for Aβ42/Aβ40 and 70.63 pg/mL for pTau181. The overlap between the two distributions, estimated by the overlapping coefficient (OVL) from the GMM model, was 0.09 for Aβ42/Aβ40 and 0.13 for pTau181, indicating a clear separation between groups. Using these cut-offs, 55.1% of participants fell within the abnormal Aβ42/Aβ40 distribution and 47.7% within the abnormal pTau181 distribution (Fig. 1A, B).

Next, we derived clinically based cut-offs by identifying the CSF values that maximised discrimination using Youden’s index from a logistic regression model. To ensure clear separation between groups, only CU and DAT participants were included, as MCI is a heterogeneous and diagnostically uncertain group that could attenuate discrimination. Because these cut-offs rely on predefined clinical labels, they may reflect underlying inaccuracies in diagnostic classification. The resulting cut-offs were 0.11 for Aβ42/Aβ40 and 65.27 pg/mL for pTau181, classifying 57.3% of participants as abnormal for Aβ42/Aβ40 and 56% for pTau181 (Fig. 1C, D).

Next, we categorized participants into AT groups (A-T-, A + T-, A-T + , A + T + ) using the cut-offs from both methods and examined the distribution of AT profiles across clinical diagnostic groups (Supplementary Fig. 3). In both approaches, at least 85% of CU participants were classified as A-T-, and approximately 87% of DAT participants were classified as A + . MCI participants showed a heterogeneous distribution, with A-T- representing the largest subgroup. Given the high concordance between methods, GMM-derived cut-offs were selected as the reference approach for all subsequent analyses. Although performance was comparable to clinically derived thresholds, GMM provides a data-driven, label-independent framework that can be consistently applied across CSF and plasma biomarker analyses.

### Plasma biomarkers in Cog-Aging-Study–Brazil

We next extended the same descriptive framework used for CSF biomarkers to plasma biomarkers, assessing distributional properties, sex differences, age-related associations, and differences across clinical diagnostic groups. Given the greater accessibility of plasma biomarkers for clinical use compared to CSF, we further evaluated their associations with cognitive and functional scales. We focused on pTau217 and pTau217/Aβ42, proposed biomarkers of Aβ pathology, as well as NfL and GFAP, which reflect more general neurodegenerative and astroglial processes involved in AD pathophysiology.

All plasma biomarkers exhibited right-skewed distributions on the original scale, which were subsequently log-transformed, yielding approximately symmetric distributions suitable for subsequent analyses (Supplementary Fig. 4). Only GFAP showed a significant association with sex, with higher levels in females (*p* = 3.41 × 10-³; Supplementary Fig. 5A–D). In the overall sample, pTau217, NfL, and GFAP were significantly correlated with age (pTau217: r = 0.17, *p* = 2.74 × 10^-^³; NfL: r = 0.39, *p* = 2.11 × 10^-^¹¹; GFAP: r = 0.32, *p* = 1.28 × 10^-^⁸), whereas no association was observed for pTau217/Aβ42 (r = 0.07, *p* = 0.23). Within diagnostic groups, pTau217 showed positive associations with age in CU and MCI participants and a negative association in DAT. Similarly, pTau217/Aβ42 showed a negative association with age in DAT, despite the absence of an overall correlation. NfL and GFAP were associated with age only in CU and MCI groups (Supplementary Fig. 5E–H). Regarding clinical diagnosis, all biomarkers showed increasing levels with disease severity, with pTau217 and pTau217/Aβ42 providing the clearest separation between diagnostic groups (Supplementary Fig. 5I–L).

Higher biomarker levels were associated with lower CERAD EVOC scores, indicating worse episodic memory performance, with larger effect sizes for pTau217, pTau217/Aβ42, and GFAP (Supplementary Fig. 6A-D). Similarly, higher biomarker levels were associated with higher FAQ scores, reflecting greater functional impairment, with more pronounced effects for pTau217, pTau217/Aβ42, and GFAP (Supplementary Fig. 6E–H). Consistent associations were observed for global disease severity: biomarker levels increased across CDR global score categories, with pTau217, pTau217/Aβ42, and GFAP showing the clearest separation (Supplementary Fig. 6I–L), and were positively associated with CDR sum of boxes, with the strongest effects observed for these same biomarkers (Supplementary Fig. 6M–P).

Finally, associations between plasma biomarkers and CSF Aβ42/Aβ40 were examined in the subset of participants with paired CSF-plasma samples (*n* = 45) to assess their relationship to amyloid pathology. pTau217, pTau217/Aβ42, and GFAP showed significant negative associations, whereas NfL showed no association. pTau217 and pTau217/Aβ42 exhibited the strongest associations with CSF Aβ42/Aβ40 (Supplementary Fig. 7).

Taken together, these analyses showed that pTau217 and pTau217/Aβ42 provided the most consistent discrimination across clinical groups, the strongest associations with cognitive and functional measures, and the closest alignment with CSF Aβ42/Aβ40, consistent with their role as markers of Aβ pathology. Accordingly, pTau217 and pTau217/Aβ42 were selected for subsequent cut-off derivation analyses.

### Data driven plasma pTau217 and pTau217/Aβ42 cut-offs in Cog-Aging-Study–Brazil

**Fig. 2:**
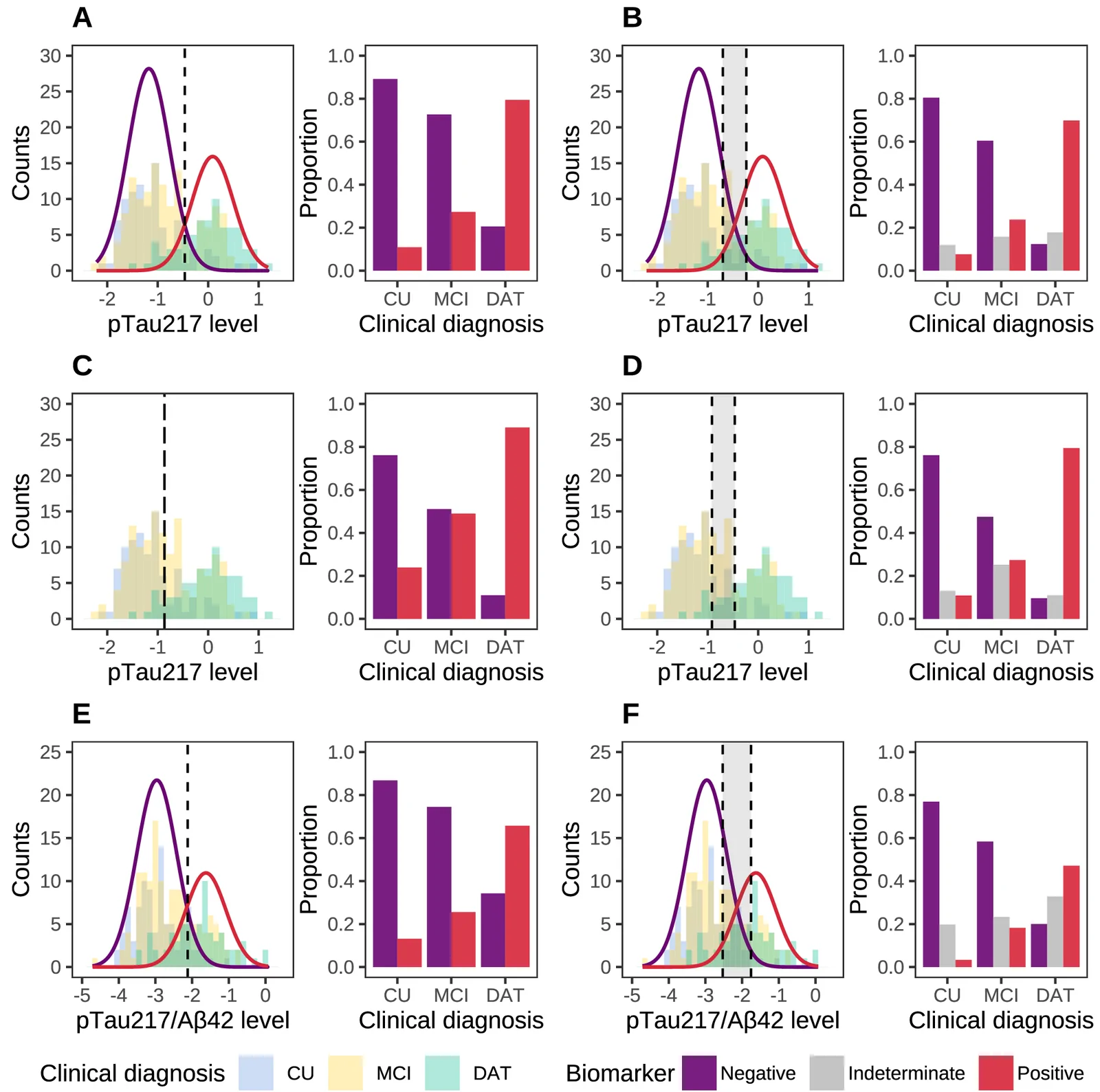
Data-driven cut-offs for plasma pTau217 and pTau217/Aβ42 in Cog-Aging-Study–Brazil and comparison with externally published cut-offs. Histograms show the distribution of biomarker levels across clinical diagnostic groups. **A**,**B** and **E**,**F** show GMM-derived distributions of normal and abnormal biomarker values. In (**A**) and (**E**), the dashed line indicates the single cut-off separating normal (left) from abnormal (right) biomarker values. In (**B**) and (**F**), the dashed lines indicate the lower and upper cut-offs of the two-cut-off approach; values below the lower cut-off were classified as biomarker-negative, values above the upper cut-off as biomarker-positive, and values within the grey-shaded area as indeterminate. **C**,**D** show the classification obtained using externally published pTau217 cut-offs from Ashton et al. Bar plots represent the proportion of participants classified as biomarker-negative, indeterminate, or biomarker-positive within each diagnostic group. Plasma pTau217 and pTau217/Aβ42 values were log-transformed. CU cognitively unimpaired, MCI mild cognitive impairment, DAT dementia of the Alzheimer’s type.

To derive cohort-specific cut-offs for plasma pTau217 and pTau217/Aβ42, we applied GMM and included all participants with available plasma samples (CU, *n* = 92; MCI, *n* = 139; DAT, *n* = 73). As in the CSF biomarker analysis, a bimodal distribution with equal variances provided the best fit (Supplementary Table 2). These models yielded a cut-off of 0.63 pg/mL and an OVL coefficient of 0.13 for pTau217, and a cut-off of 0.12 and an OVL of 0.22 for pTau217/Aβ42. Using these cut-offs, 34.9% of participants were classified as pTau217+ (Fig. 2A), and 31.2% as pTau217/Aβ42 + (Fig. 2E). Similar to the CSF results, the status of pTau217 and pTau217/Aβ42 showed high concordance with the clinical diagnosis. At least 86% of CU participants fell into the normal biomarker distribution, whereas at least 65% of DAT participants fell into the abnormal distribution.

Following the BBM Workgroup’s recommendation to use a two-cut-off approach to define three categories: positive, intermediate, and negative^8^, we evaluated combinations of lower and upper cut-offs, introducing a “grey zone” or indeterminate category between them. Because GMM generated probabilities of class membership for each participant, we derived cut-offs at probability thresholds of ≥0.80, ≥0.85, or ≥0.90, as previously applied^9^ (Supplementary Fig. 8).

For pTau217, a probability threshold of ≥0.80 (lower cut-off, 0.52 pg/mL; upper cut-off, 0.76 pg/mL) classified 57.6% of participants as pTau217-, 30.3% as pTau217 + , and 12.1% as indeterminate (Supplementary Fig. 9C). Increasing the threshold to ≥0.85 (lower cut-off, 0.50 pg/mL; upper cut-off, 0.79 pg/mL) resulted in 57.9% classified as pTau217-, 29.9% as pTau217 + , and 15.2% as indeterminate (Fig. 2B, Supplementary Fig. 9E). At ≥0.90 (lower cut-off, 0.47 pg/mL; upper cut-off, 0.84 pg/mL), 53.6% were classified as pTau217-, 26.6% as pTau217 + , and 19.7% as indeterminate (Supplementary Fig. 9G). Across all thresholds, agreement with clinical diagnosis remained high, with about 80% of CU participants classified as pTau217-, and around 70% of DAT participants as pTau217 + . The proportion of indeterminate cases was evenly distributed across diagnostic groups.

Similar results were observed for pTau217/Aβ42. At a threshold of ≥0.80 (lower cut-off, 0.09; upper cut-off, 0.16), 57.7% of participants were classified as pTau217/Aβ42-, 22.5% as pTau217/Aβ42 + , and 19.8% as indeterminate (Supplementary Fig. 9D). At ≥0.85 (lower cut-off, 0.08; upper cut-off, 0.17), 55.0% were pTau217/Aβ42-, 20.5% pTau217/Aβ42 + , and 24.5% indeterminate (Fig. 2F, Supplementary Fig. 9F). At ≥0.90, 48.7% were classified as pTau217/Aβ42-, 17.8% as pTau217/Aβ42 + , and 33.5% as indeterminate (Supplementary Fig. 9H). In all cases, concordance with clinical diagnosis was high, though lower than for pTau217 alone. Approximately 80% of CU participants were classified as pTau217/Aβ42-, whereas about 50% of DAT participants were classified as pTau217/Aβ42 + . The proportion of indeterminate cases was evenly distributed across diagnostic groups, but at the ≥0.90 threshold, it was well beyond the recommended range^8^.

Next, we assessed the performance of plasma cut-offs in the subset of participants with paired CSF samples, thereby providing available amyloid status (A-, n = 17; A + , n = 28). For both biomarkers, the models using two cut-offs at the ≥0.85 probability threshold provided the best overall performance, with a PPV of 0.88 for pTau217 and 0.89 for pTau217/Aβ42, and an NPV of 0.81 for pTau217 and 0.73 for pTau217/Aβ42. These findings were in line with the 0.90 PPV/NPV threshold recommended by the BBM Workforce^8^, although lower for pTau217/Aβ42, supporting the use of data-driven cut-off calculation in populations without gold-standard reference measures such as CSF or PET (Table 2).

**Table 2:** Predictive performance of plasma pTau217 and pTau217/Aβ42 cut-offs in Cog-Aging-Study–Brazil

Biomarker	Method	Threshold	Single cut-off	Lower cut-off	Upper cut-off	Indeterm (%)	PPV (95% CI)	NPV (95% CI)	Sensitivity (95% CI)	Specificity (95% CI)
pTau217	GMM	-	≥0.63	-	-	-	0.85 (0.68–0.94)	0.72 (0.49–0.88)	0.82 (0.64–0.92)	0.76 (0.53–0.90)
	Ashton et al. (2024)	–	>0.42	–	–	–	0.83 (0.66–0.93)	0.80 (0.55–0.93)	0.89 (0.73–0.96)	0.71 (0.47–0.87)
	GMM	0.80	–	≤0.52	≥0.76	6.7	0.88 (0.70–0.96)	0.76 (0.53–0.90)	0.85 (0.66–0.94)	0.81 (0.57–0.93)
	GMM	0.85	–	≤0.50	≥0.79	11.1	0.88 (0.69–0.96)	0.81 (0.57–0.93)	0.88 (0.69–0.96)	0.81 (0.57–0.93)
	GMM	0.90	–	≤0.47	≥0.84	17.8	0.86 (0.67–0.95)	0.80 (0.55–0.93)	0.86 (0.67–0.95)	0.80 (0.55–0.93)
	Ashton et al. (2024)	–	–	<0.40	>0.63^a^	8.9	0.85 (0.68–0.94)	0.86 (0.60–0.96)	0.92 (0.75–0.98)	0.75 (0.51–0.9)
pTau217/Aβ42	GMM	–	≥0.12	–	–	–	0.88 (0.69–0.96)	0.68 (0.46–0.85)	0.78 (0.59–0.89)	0.81 (0.57–0.93)
	GMM	0.80	–	≤0.09	≥0.16	14	0.90 (0.71–0.97)	0.69 (0.44–0.86)	0.79 (0.60–0.91)	0.85 (0.58–0.96)
	GMM	0.85	–	≤0.08	≥0.17	20.9	0.89 (0.69–0.97)	0.73 (0.48–0.89)	0.81 (0.60–0.92)	0.85 (0.58–0.96)
	GMM	0.90	–	≤0.07	≥0.19	34.9	0.88 (0.66–0.97)	1 (0.74–1)	1 (0.8–1)	0.85 (0.58–0.96)

*Indeterm* indeterminate, *PPV* positive predictive value, *NPV* negative predictive value, *CI* confidence interval. Cut-offs for pTau217 are expressed in pg/mL and were evaluated in the subset of participants with paired CSF-plasma samples (*n* = 45).

Finally, we compared the performance of cohort-specific cut-offs with externally published thresholds. Because biomarker values depend on the analytical platform, we compared only studies using the same equipment (Simoa) and assay (Alzpath for pTau217, Neurology 4-plex for Aβ42). Ashton et al. ^4^ derived pTau217 cut-offs in the same conditions in cohorts from North America and Europe. Using their published single cut-off (0.42 pg/mL), 50% of participants were classified as pTau217 + , a higher proportion than with the cohort-specific threshold. Concordance with clinical diagnosis remained high; however, a substantially larger proportion of MCI participants were classified as pTau217+ (48.9% vs 27.3% with the cohort-specific cut-off; Fig. 2C). In contrast, the two-cut-off approach (lower cut-off: 0.40 pg/mL; upper cut-off: 0.63 pg/mL) yielded distributions more comparable to those obtained with cohort-specific cut-offs, with 47% of participants classified as pTau217-, 34.9% as pTau217 + , and 18.1% as indeterminate. The MCI group showed the highest proportion of indeterminate cases (25.2%; Fig. 2D), primarily reflecting a reclassification of participants previously categorised as pTau217+ under the single-threshold approach, while the proportion classified as pTau217- remained largely unchanged. In the subset of 45 participants with paired CSF samples, both approaches showed similar overall performance, although the published cut-offs yielded slightly higher NPV and sensitivity at the expense of PPV and specificity (Table 2). No external cut-offs for pTau217/Aβ42 using comparable analytical conditions were available, precluding a similar comparison for this biomarker.

### Plasma biomarkers in GeNED.ar–Argentina

We next applied the same descriptive framework used for plasma biomarkers in Cog-Aging-Study–Brazil to GeNED.ar–Argentina. As in Cog-Aging-Study–Brazil, all plasma biomarkers showed right-skewed distributions on the original scale, which became approximately symmetric after log-transformation (Supplementary Fig. 10). In contrast to Cog-Aging-Study–Brazil, all biomarkers showed higher levels in males (Supplementary Fig. 11A–D). This pattern is likely explained by differences in age distribution (mean age males: 73.2 years; mean age females: 71 years) and *APOE*-ε4 frequency (males: 20%; females: 13.3%).

In the overall sample, all biomarkers were significantly correlated with age (pTau217: r = 0.48, *p* = 4.96 × 10^−9^; *p*Tau217/Aβ42: r = 0.41, *p* = 1.31 × 10^−6^; NfL: r = 0.67, *p* < 2.2 × 10^−16^; GFAP: r = 0.51, *p* = 4.15 × 10^−10^), with generally stronger associations than in Cog-Aging-Study–Brazil. Within diagnostic groups, age associations were more heterogeneous, with pTau217 and pTau217/Aβ42 associated with age only in CU participants, NfL with CU and MCI participants, and GFAP with CU and DAT participants (Supplementary Fig. 11E–H). As observed in Cog-Aging-Study–Brazil, all biomarkers increased with disease severity across diagnostic groups (Supplementary Fig. 11I–L).

No significant associations were observed between biomarker levels and FAQ scores, likely linked to the high proportion of CU participants (Supplementary Fig. 12A–D). However, biomarker levels increased across CDR global score categories, consistent with greater global disease severity (Supplementary Fig. 12E–H). CERAD EVOC and CDR sum of boxes scores were not available in GeNED.ar–Argentina, precluding analyses equivalent to those performed in Cog-Aging-Study–Brazil.

Taken together, these descriptive analyses showed that plasma pTau217 and pTau217/Aβ42 consistently separated across clinical diagnostic groups in GeNED.ar–Argentina, supporting their selection for subsequent cut-off derivation analyses in this cohort.

### Data-driven plasma pTau217 and pTau217/Aβ42 cut-offs in GeNED.ar–Argentina

**Fig. 3:**
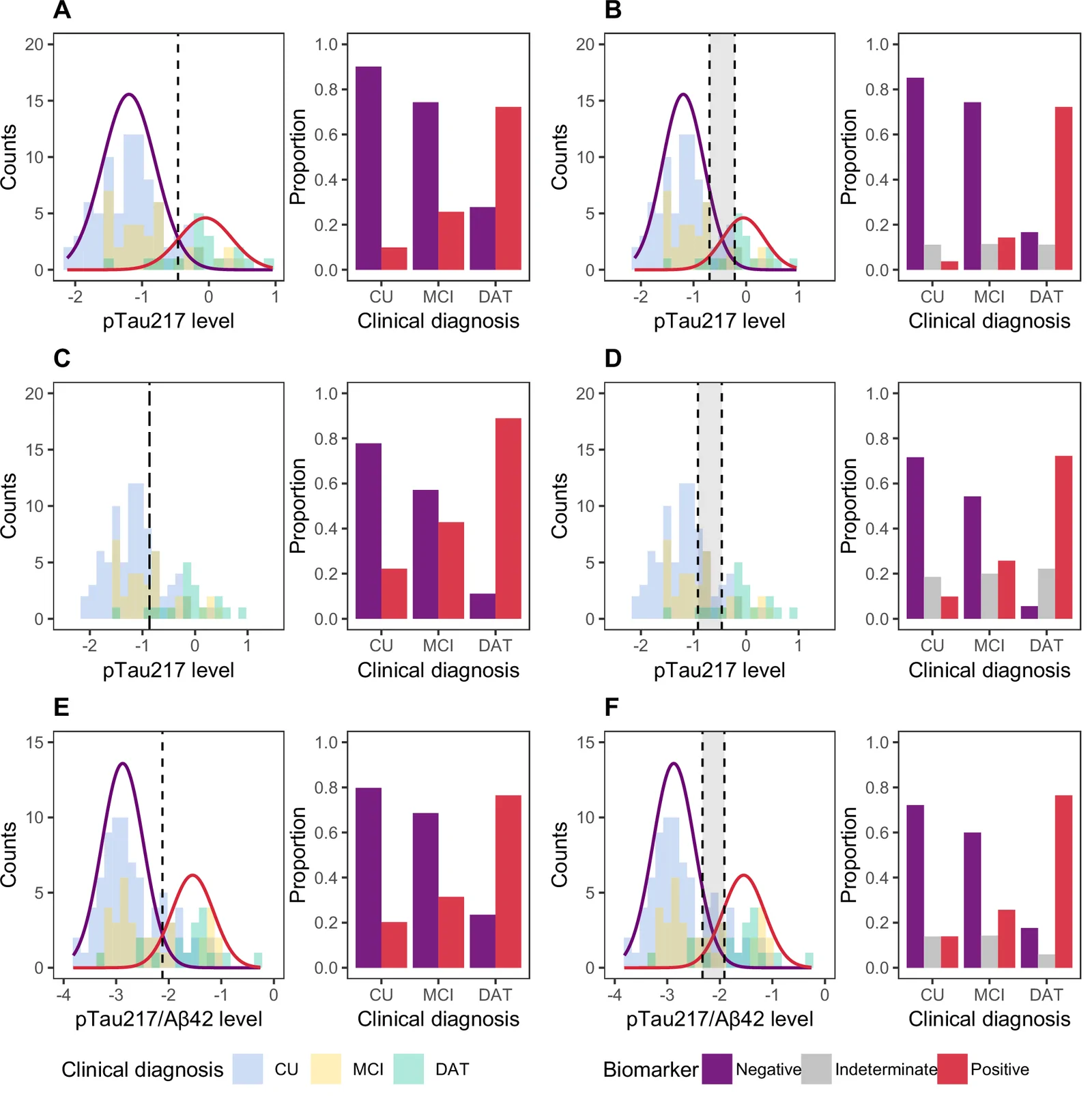
Data-driven cut-offs for plasma pTau217 and pTau217/Aβ42 in GeNED.ar–Argentina and comparison with externally published cut-offs. Histograms show the distribution of biomarker levels across clinical diagnostic groups. **A**,**B** and **E**,**F** show GMM-derived distributions of normal and abnormal biomarker values. In (**A**) and (**E**), the dashed line indicates the single cut-off separating normal (left) from abnormal (right) biomarker values. In (**B**) and (**F**), the dashed lines indicate the lower and upper cut-offs of the two-cut-off approach; values below the lower cut-off were classified as biomarker-negative, values above the upper cut-off as biomarker-positive, and values within the grey-shaded area as indeterminate. **C**,**D** show the classification obtained using externally published pTau217 cut-offs from Ashton et al. Bar plots represent the proportion of participants classified as biomarker-negative, indeterminate, or biomarker-positive within each diagnostic group. Plasma pTau217 and pTau217/Aβ42 values were log-transformed. CU cognitively unimpaired; MCI, mild cognitive impairment; DAT, dementia of the Alzheimer’s type.

To derive plasma pTau217 and pTau217/Aβ42 cut-offs in GeNED.ar–Argentina, we applied the same approach used in Cog-Aging-Study–Brazil. Models with one or two cut-offs, including an indeterminate category, were calculated for all participants (CU, *n* = 81; MCI, *n* = 35; DAT, *n* = 18). As in Cog-Aging-Study–Brazil, a bimodal distribution with equal variances provided the best fit (Supplementary Table 3). These models identified a single cut-off of 0.63 pg/mL for pTau217 (OVL coefficient, 0.15), and 0.12 for pTau217/Aβ42 (OVL, 0.09). Using these thresholds, 22.4% of participants were classified as pTau217+ (Fig. 3A), and 30.5% as pTau217/Aβ42 + (Fig. 3E). Notably, these cut-offs matched those from Cog-Aging-Study–Brazil. Biomarker status also showed high agreement with clinical diagnosis: at least 79% of CU participants were within the normal range, and at least 72% of DAT participants were within the abnormal range.

We next derived lower and upper cut-offs using the same probability thresholds as in Cog-Aging-Study–Brazil (Supplementary Fig. 13). For pTau217, at the ≥0.80 threshold (lower cut-off, 0.52 pg/mL; upper cut-off, 0.77 pg/mL), 73.1% were pTau217-, 16.4% pTau217 + , and 10.5% indeterminate (Supplementary Fig. 14C). At ≥0.85 (lower cut-off, 0.50 pg/mL; upper cut-off, 0.81 pg/mL), 73.1% were pTau217-, 15.7% pTau217 + , and 11.2% indeterminate (Fig. 3B and Supplementary Fig. 14E). At ≥0.90 (lower cut-off, 0.47 pg/mL; upper cut-off, 0.86 pg/mL), 68.6% were pTau217-, 15.7% pTau217 + , and 15.7% indeterminate (Supplementary Fig. 14G). Across all thresholds, concordance with clinical diagnosis remained high, with approximately 80% of the CU participants classified as pTau217- and about 72% of DAT participants as pTau217 + . The proportion of indeterminate cases was evenly distributed across diagnostic groups.

When applying the published pTau217 cut-offs from Ashton et al. ^4^, we observed a classification pattern broadly comparable to that seen in Cog-Aging-Study–Brazil. The single-threshold approach classified a higher proportion of participants as pTau217+ (36.7%) than the cohort-specific threshold, while maintaining high concordance with clinical diagnosis. This effect was particularly evident in the MCI group, where a substantially larger proportion was classified as pTau217+ (42.9 vs 25.7% using the cohort-specific cut-off; Fig. 3C). By contrast, the two-cut-off strategy resulted in lower proportions of participants classified as pTau217- (58.2%) and pTau217+ (22.4%), with a corresponding increase in indeterminate cases (19.4%) compared with the cohort-specific approach. As in Cog-Aging-Study–Brazil, the increased indeterminate classifications in MCI participants (20.0%; Fig. 3D) primarily reflected reclassification of individuals previously considered pTau217+ under the single-threshold strategy (42.9 vs 25.7%), whereas the proportion classified as pTau217- remained relatively stable.

For pTau217/Aβ42, at ≥0.80 (lower cut-off, 0.10; upper cut-off, 0.14), 61.8% were pTau217/Aβ42-, 26.7% pTau217/Aβ42 + , and 11.5% indeterminate (Supplementary Fig. 14D). At ≥0.85 (lower cut-off, 0.097; upper cut-off, 0.15), 61.8% were pTau217/Aβ42-, 25.2% pTau217/Aβ42 + , and 13.0% indeterminate (Fig. 3F, Supplementary Fig. 14F). Finally, at ≥0.90 (lower cut-off, 0.09; upper cut-off, 0.16), 60.3% were pTau217/Aβ42-, 23.7% pTau217/Aβ42 + , and 16.0% indeterminate (Supplementary Fig. 14G). Concordance with clinical diagnosis remained high. Approximately 70% of CU participants were classified as pTau217/Aβ42-, and about 76% of DAT participants were classified as pTau217/Aβ42 + . The proportion of indeterminate cases was evenly distributed across CU and MCI groups, whereas only one DAT participant fell into the indeterminate category.

### Stability of pTau217 cut-off estimation in pooled South American cohorts

To assess the stability of pTau217 cut-off estimates with an increased sample size, we conducted complementary pooled analyses combining participants from Cog-Aging-Study–Brazil and GeNED.ar–Argentina. Because cohort-specific analyses yielded highly comparable cut-off values, pooled analyses were used to evaluate whether threshold estimates remained consistent when derived from a larger combined sample.

We applied the same GMM framework to all participants with available plasma samples (CU, *n* = 173; MCI, *n* = 174; DAT, *n* = 91). As in the individual cohort analysis, a bimodal distribution with equal variances provided the best fit (Supplementary Table 4). Notably, pooled analyses yielded the same single cut-offs obtained independently in both cohorts: 0.63 pg/mL for pTau217 (OVL coefficient, 0.13) and 0.12 for pTau217/Aβ42 (OVL coefficient, 0.18). Using these thresholds, 31.1% participants were classified as pTau217+ (Supplementary Fig. 15A), and 31.6% as pTau217/Aβ42 + (Supplementary Fig. 15B). Similar to the cohort-specific analyses, biomarker classifications showed high concordance with clinical diagnosis, with at least 83% of CU participants classified within the normal distribution and at least 67% of DAT classified within the abnormal distribution.

We next evaluated the two-cut-off approach using probability thresholds of ≥0.80, ≥0.85, or ≥0.90. For pTau217, lower and upper cut-off values were highly similar to those obtained in the individual cohorts. Classification distributions were stable across thresholds, with only modest variation in category proportions. The proportion classified as pTau217+ remained relatively stable across thresholds (30.3% to 26.6%), as did the proportion classified as pTau217- (57.6 to 53.6%), while indeterminate classifications gradually increased from 12.1 to 19.7%, particularly at the ≥0.90 threshold (Supplementary Fig. 15C, E, G). Concordance with clinical diagnosis remained high, with approximately 80% of CU participants classified as pTau217- and 60-70% of DAT participants as pTau217 + . Indeterminate classifications were evenly distributed across diagnostic groups.

For pTau217/Aβ42, the lower and upper cut-offs were also highly comparable to those obtained in the individual cohorts. Across probability thresholds, classification distributions were stable, with only modest variation in category proportions. The proportion classified as pTau217/Aβ42+ showed minimal variation across thresholds (28.7 to 24.9%), as did the proportion classified as pTau217/Aβ42- (74.8 to 71.3%), while indeterminate classifications increased gradually from 22.9% to 30.2%, particularly at the ≥0.90 threshold (Supplementary Fig. 15D, F, H). Concordance with clinical diagnosis was also high, though consistently lower than for pTau217 alone. Approximately 75% of CU participants were classified as pTau217/Aβ42-, whereas around 50% of DAT participants were classified as pTau217/Aβ42 + .

Finally, to facilitate comparison with the cohort-specific analyses, we also evaluated plasma cut-off performance in the subset of participants with paired CSF samples from Cog-Aging-Study–Brazil (A-, *n* = 17; A + , *n* = 28). For pTau217, the two-cut-off model at the ≥0.85 probability threshold achieved the best overall performance, yielding a PPV of 0.88 and an NPV of 0.81. For pTau217/Aβ42, the model using the ≥0.80 probability threshold showed the best balance between PPV (0.90) and NPV (0.69), whereas the ≥0.90 threshold provided slightly higher NPV at the expense of a larger indeterminate category (Supplementary Table 5). Overall, these findings were consistent with the cohort-specific analyses, supporting the stability of GMM-derived plasma cut-offs when estimated in a larger pooled sample.

### Association of plasma pTau217 and pTau217/Aβ42 levels with covariates

**Fig. 4:**
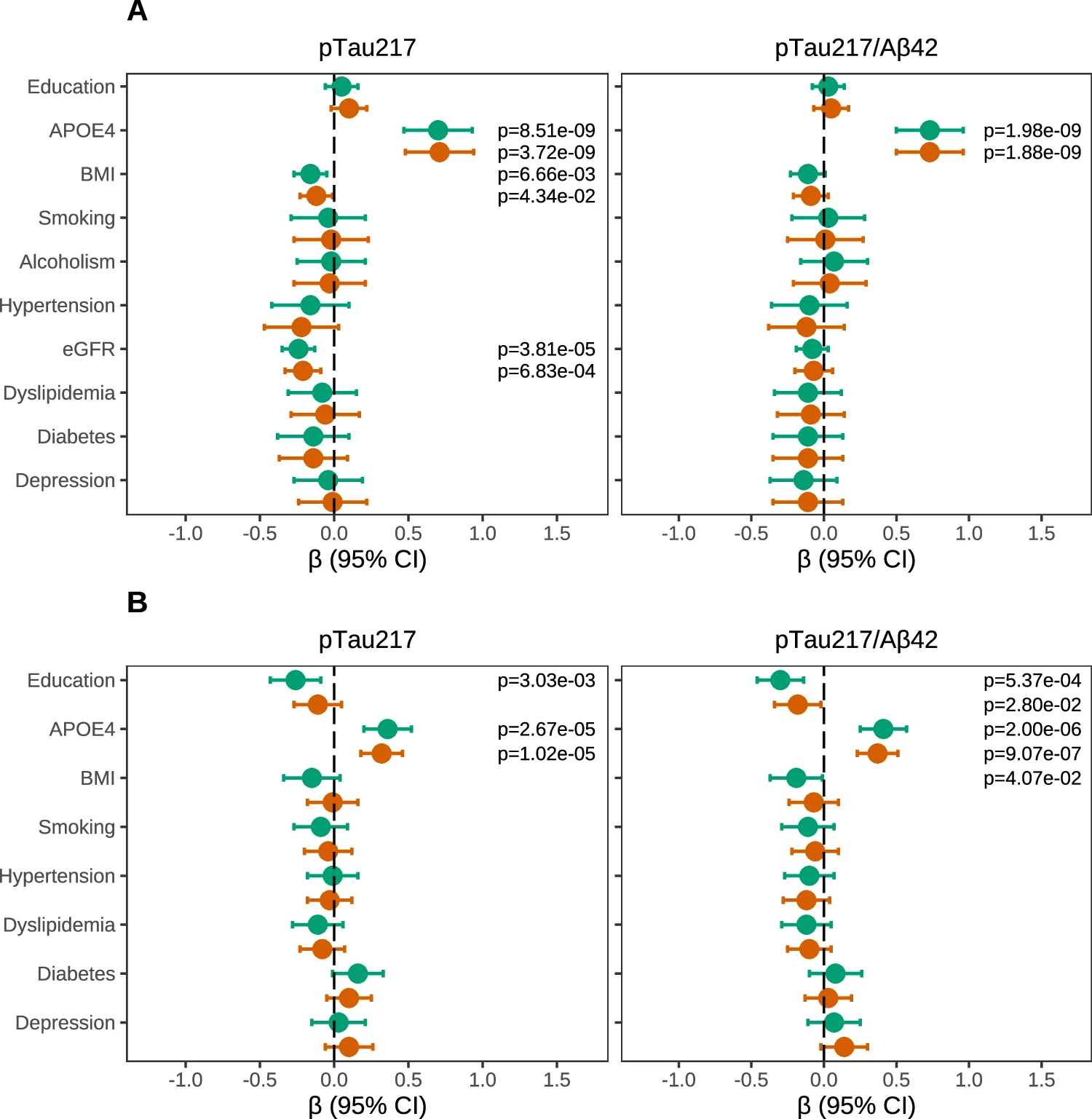
Association of plasma pTau217 and pTau217/Aβ42 with covariates in Cog-Aging-Study–Brazil and GeNED.ar–Argentina. Forest plots summarise associations from univariable models (green) and models adjusted for age and sex (orange) in Cog-Aging-Study-Brazil (**A**, *n* = 304) and GeNED.ar-Argentina (**B**, *n* = 134). Effect estimates (*β*) are presented as linear regression coefficients with 95% confidence intervals. Continuous variables were standardised before analysis. Nominal p-values are shown only for associations with *p* < 0.05. BMI body mass index, CI confidence interval, eGFR estimated glomerular filtration rate, *p* *p*-value.

We next evaluated whether modifiable risk factors for dementia and *APOE* genotype influenced plasma levels of pTau217 and pTau217/Aβ42. Both unadjusted and age- and sex-adjusted associations were assessed in all participants from Cog-Aging-Study–Brazil and GeNED.ar–Argentina. To allow comparison of effect sizes, biomarkers were *z*-transformed. In Cog Aging Study-Brazil, the presence of at least one *APOE-ε4* allele, lower eGFR, and lower BMI were significantly associated with higher pTau217 levels. These associations remained significant after adjusting for age and sex (Fig. 4A). By contrast, pTau217/Aβ42 levels were significantly associated only with *APOE-ε4* status, suggesting that the biomarker ratio is not sensitive to changes in BMI or eGFR. Adjusting for age and sex did not alter these results (Fig. 4B). We observed similar patterns in GeNED.ar–Argentina. Lower education and the presence of at least one *APOE-ε4* allele were significantly associated with higher pTau217 levels, but after adjusting for age and sex, only *APOE-ε4* status remained significant (Fig. 4C). For pTau217/Aβ42, higher levels were associated with lower education, the presence of at least one *APOE-ε4* allele, and lower BMI. However, after adjusting for age and sex, only education and *APOE-ε4* status remained significant (Fig. 4D). eGFR data were not available in GeNED.ar–Argentina, so kidney function could not be accounted for in this cohort.

The association of *APOE-*ε4 with plasma biomarkers prompted us to investigate whether ancestry admixture, observed in Brazilian and Argentinian populations, affects this relationship in CSF and plasma. Latin American populations exhibit significant genetic and phenotypic diversity due to the admixture of Indigenous Native American, European, and African ancestries^10^. The Brazilian population is predominantly of European and African ancestry. Consistent with this, Cog-Aging-Study–Brazil showed varying levels of African ancestry among participants, evenly distributed across the different diagnostic groups (Supplementary Fig. 17A and Supplementary Table 6). Notably, previous studies reported that African ancestry may attenuate the negative impact of *APOE-ε4* on AD risk^11,12^. First, we tested whether African ancestry modified the association between *APOE-ε4* and CSF Aβ42/Aβ40 (*APOE-ε4*×African ancestry) in participants with available CSF Aβ42/Aβ40 and genetic data (*n* = 45). The interaction was significant (*β* = 0.09, SE = 0.04, *p* = 0.04), indicating that the association between African ancestry and CSF Aβ42/Aβ40 differed by *APOE-ε4* status (Supplementary Fig. 18). In stratified analyses, higher African ancestry was associated with lower CSF Aβ42/Aβ40 among non-*APOE-ε4* carriers (*β* = −0.08, SE = 0.03, *p* = 0.03), but not among *APOE-ε4* carriers (*β* = 0.02, SE = 0.03, *p* = 0.55). Next, we tested the same interaction term in participants with available plasma biomarkers and genetic data (*n* = 147) and found no significant associations (pTau217, *p* = 0.82; pTau217/Aβ42, *p* = 0.86). Since African ancestry is negligible in the Argentinian population (Supplementary Fig. 17B, Supplementary Table 6), this analysis was not performed in GeNED.ar–Argentina.

## Discussion

In this study, we examined the concordance between clinical diagnosis and CSF-defined amyloid pathology and assessed the diagnostic performance of plasma pTau217 and pTau217/Aβ42 in two genetically admixed South American cohorts. Using an unbiased, data-driven approach to derive CSF and plasma cut-offs, we found high concordance between plasma biomarker-predicted and CSF-defined amyloid status in Cog-Aging-Study–Brazil. Applying a two-cut-off strategy with an indeterminate zone further improved predictive performance. In GeNED.ar–Argentina, a cohort without CSF but with comparable socioeconomic, demographic, and clinical characteristics, concordance between plasma-based classifications and clinical diagnosis suggests that this approach may be valuable in populations lacking gold-standard measures and could offer a practical framework for implementation in LMIC memory clinics.

Diagnostic approaches for AD vary substantially across countries and medical specialties^13^. In HICs, over 50% of individuals with dementia remain undiagnosed, and the rate may be even higher in LMICs^14^. Research using autopsy and biomarkers in HICs indicates that 15-30% of people who meet clinical criteria for DAT do not actually have underlying Aβ or tau pathology^15,16^. Misdiagnosis is particularly common in the early stages, when symptoms are mild, and only a small percentage of those with MCI receive an official diagnosis. The use of PET imaging and CSF biomarkers has substantially improved diagnostic accuracy, with dementia specialists increasing their accuracy from 73 to 91% when biomarker data were available^6^. In Cog-Aging-Study–Brazil, the agreement between clinical and CSF-based diagnosis (around 85% for DAT) suggests that the misdiagnosis rate in this LMIC cohort is comparable to that in HICs. Additionally, the benefit of CSF biomarkers in improving diagnosis appears to be similar, suggesting that comprehensive clinical assessments can achieve comparable accuracy even in less-resourced settings.

Two strategies have been proposed for defining plasma pTau217 cut-offs. The traditional single-cut-off approach classifies individuals as biomarker-positive or negative, while the emerging two-cut-off strategy introduces a “grey zone” (typically 15–20% of individuals) to account for assay variability and biological heterogeneity^17,18^. Individuals within this indeterminate range may undergo confirmatory testing (such as CSF or PET), thereby reducing misclassification and enhancing workflow efficiency. In the subset of participants with paired CSF-plasma samples in Cog-Aging-Study–Brazil, incorporating a grey zone at the 0.85 threshold representing 11.1% of participants increased PPV from 0.85 to 0.88 and NPV from 0.72 to 0.81 for CSF-based amyloid positivity, approaching the approximately 0.90 benchmark proposed by the BBM Workgroup for using plasma biomarkers in secondary care^8^. In contrast, the pTau217/Aβ42 ratio yielded a slightly larger grey zone (~21%) and did not improve overall performance: the PPV increased to 0.89, but the NPV decreased to 0.73. Using these cut-offs across the whole dataset, at least 70% of clinically diagnosed DAT and approximately 24% of MCI participants tested positive for pTau217, dropping to roughly 47% and 18%, respectively, when using pTau217/Aβ42. In GeNED.ar–Argentina, where CSF data were unavailable, a similar pattern was observed: pTau217 with a grey zone at the 0.85 threshold showed approximately 70% concordance with DAT and around 30% with MCI. pTau217/Aβ42 produced comparable results, with slightly higher concordance for DAT (~76%) and similar concordance for MCI (~20%), and higher concordance with clinical diagnosis than in Cog-Aging-Study–Brazil. Interestingly, the cut-offs obtained were identical in both cohorts. Moreover, pooled analyses combining both datasets yielded the same single cut-offs obtained in each dataset, further supporting the stability of the GMM-derived thresholds.

Conversely, the finding that pTau217/Aβ42 produced a larger grey zone contrasts with recent reports showing that pTau217/Aβ42 reduced the proportion of indeterminate cases despite not improving diagnostic accuracy^19,20^. One possible explanation is methodological. In both studies, plasma Aβ42 was measured using mass spectrometry-based approaches, which have shown superior discriminative performance for amyloid pathology defined by CSF Aβ42/Aβ40 compared with immunoassay-based methods. In our study, both pTau217 and Aβ42 were measured on the Simoa platform, which may have contributed to the slightly larger grey zone observed for pTau217/Aβ42. In addition, plasma Aβ42 and Aβ40 have been reported to be among the plasma biomarkers most sensitive to preanalytical variability, showing decreases greater than 10% under storage or centrifugation delays^21^, which may have further influenced the performance of the ratio. These observations also raise the broader question of how plasma cut-offs should be defined and applied across populations, as the platform used and preanalytical factors may differ from those of the populations in which current thresholds were originally derived.

As a complementary approach to deriving cohort-specific thresholds, assessing the transferability of externally published cut-offs may also be important for LMIC populations with limited access to CSF or PET. Since transferability can only be accurately evaluated when cut-offs are established under similar conditions, we tested the performance of previously published pTau217 cut-offs from North American and European cohorts using the same analytical procedures^4^. Overall, these thresholds worked well in both South American cohorts, demonstrating high agreement with clinical diagnosis and comparable results against CSF-defined amyloid status in Cog-Aging-Study–Brazil. Nevertheless, the single-threshold method identified a substantially larger proportion of MCI participants as pTau217 + , while adopting the two-cut-off approach reclassified many of these individuals into the indeterminate group, producing distributions more aligned with those derived from cohort-specific thresholds.

Importantly, the diagnostic performance seen in these South American cohorts was also largely consistent with findings from other underrepresented populations, including cohorts from East and South Asia and Native American-enriched groups. In these populations, plasma pTau217 similarly demonstrated high agreement with clinically diagnosed DAT^22–27^. When considering the comparable performance to predominantly White cohorts from North America and Europe, these results support the potential of pTau217-based biomarkers to be applicable across diverse populations, especially within a two-cut-off model. However, differences in genetic background, comorbidities, socioeconomic factors, and preanalytical conditions may still affect biomarker levels and the definition of optimal thresholds. This underscores the need for ongoing validation in heterogeneous populations as plasma biomarkers move toward routine clinical implementation.

To interpret plasma pTau217 and pTau217/Aβ42 results accurately, it is essential to understand which factors influence biomarker variability. Previous research from HICs with primarily European-ancestry groups shows that *APOE-ε4* and chronic kidney disease are associated with elevated pTau217 levels^28,29^. Our study extends this evidence to LMICs with more diverse socioeconomic and genetic backgrounds, filling a key gap in the applicability of BBMs beyond well-studied populations. In Cog-Aging-Study–Brazil, pTau217 and pTau217/Aβ42 increased with age in CU and MCI participants, whereas inverse associations were observed in DAT participants, aligning with previous reports showing positive age associations in amyloid-negative individuals and negative associations in amyloid-positive individuals^19^. Furthermore, carriers of at least one *APOE-ε4* allele, as well as those with lower BMI or eGFR, showed higher pTau217 levels, and these associations remained significant after adjustment for age and sex. Conversely, pTau217/Aβ42 was unaffected by BMI or eGFR, consistent with prior research^19^, though a slight decrease in NPV was noted. This stability could favor pTau217/Aβ42 in settings where comorbidities affecting renal function or nutrition are common or hard to measure, but its lower NPV warrants caution in ruling out pathology. In GeNED.ar–Argentina, pTau217 and pTau217/Aβ42 increased with age only in CU participants. No associations were found in DAT individuals, likely due to the small sample size of this clinical diagnostic group. Additionally, *APOE-ε4* similarly influenced both pTau217 and pTau217/Aβ42 levels. Notably, lower educational attainment was associated with higher pTau217/Aβ42 levels, even after adjusting for age and sex, suggesting that educational attainment may serve as a proxy for broader social determinants of biomarker levels and underscoring the importance of considering contextual factors when using pTau217 for diagnosis in LMICs.

Since African ancestry has been suggested to attenuate the detrimental effect of the *APOE-ε4* allele on AD risk^11,12^, we investigated whether the global African ancestry component modifies the effect of *APOE-ε4* on CSF and plasma biomarkers in participants from Cog-Aging-Study–Brazil, with a global African ancestry proportion ranging from 0% to 99%. We observed that a higher proportion of African ancestry was associated with lower CSF Aβ42/Aβ40 levels in individuals without the *APOE-ε4* allele. Conversely, in plasma, African ancestry did not affect the relationship between *APOE-ε4* and either pTau217 or pTau217/Aβ42. These results suggest that ancestry-related differences in AD risk may be more pronounced for central amyloid pathology than for peripheral pTau217 levels, highlighting the importance of considering the biomarker source when assessing ancestry effects in admixed populations.

Interestingly, the link between global African ancestry and CSF Aβ42/Aβ40 levels suggests that African ancestry alone does not protect against AD. Instead, higher African ancestry appears to be associated with increased amyloid pathology in *APOE-ε4* non-carriers, as indicated by lower CSF Aβ42/Aβ40 levels, suggesting a potential higher AD risk linked to this background. Although *APOE-ε4* generally promotes amyloid accumulation, this effect is attenuated in carriers, who already have lower CSF Aβ42/Aβ40 levels. One possible reason is that during human evolution, the *ε4* allele in people of African descent may have lost some of the genetic modifiers that increase its harmful effects, beyond the two canonical SNPs (rs429358 and rs7412) that define the allele. These findings align with previous reports showing greater amyloid deposition in Black individuals versus White individuals^30^. Further support for this hypothesis comes from a community-based cohort of admixed Brazilians, which found that global African ancestry did not affect cognition in *APOE-ε4* carriers. However, local *APOE* ancestry was associated with poorer cognitive outcomes among *APOE-ε4* non-carriers with non-European *APOE* backgrounds^31^. Together, these results indicate that ancestry-related effects are complex and involve more than just a single global component, encouraging future studies examining how different combinations of genetic ancestry, beyond global African ancestry alone, may impact biomarker levels and their clinical implications in underrepresented populations.

Key strengths of this study include the evaluation of plasma pTau217 and pTau217/Aβ42 ratio in two genetically admixed South American cohorts, along with the integration of genetic, sociodemographic, and ancestry-related factors to explore what influences biomarker variability. Limitations include the small number of participants with paired CSF and plasma samples in Cog-Aging-Study–Brazil, the lack of CSF samples in GeNED.ar–Argentina, which limited direct comparison of plasma biomarker performance between cohorts, and the use of global rather than local ancestry to assess the impact of *APOE-ε4* on plasma biomarkers, which might underestimate the effect of ancestry-specific modulation of *APOE-ε4*-related AD risk. Additionally, both cohorts were recruited from single centers, which may not fully represent the genetic diversity within each country. Although CSF was used as the reference standard, validation against neuropathologically confirmed cases across diverse populations is still necessary. Moreover, biomarker measurements can vary depending on the analytical platform used. While Simoa offers high sensitivity, differences among assay technologies should be considered when comparing absolute values or cut-offs across studies. Lastly, since detailed medication data were not consistently available, we could not separate the effects of comorbidities from their pharmacological treatments, and the observed associations might reflect their combined impact of both.

In conclusion, plasma pTau217 and pTau217/Aβ42 predicted amyloid positivity in Cog-Aging-Study–Brazil and showed concordance with clinical diagnosis comparable to that reports in studies from HICs in both Cog-Aging-Study–Brazil and GeNED.ar–Argentina. Beyond *APOE* genotype, plasma biomarker variation was partly influenced by sociodemographic and health-related factors, underscoring the importance of considering clinical context when interpreting biomarker results. Although global African ancestry appears to modulate the effect of *APOE-*ε4 on CSF biomarker levels, the absence of an effect on plasma biomarkers warrants further research in larger cohorts to confirm or challenge this finding. Overall, these results support the use of plasma pTau217-based biomarkers to assist dementia assessments in settings with limited access to CSF, while highlighting the need for additional validation in larger, more diverse LMIC populations.

## Methods

This study complied with all relevant ethical regulations. The Cog-Aging-Study–Brazil protocol was approved by the Ethics Committee of the University Hospital of the Federal University of Minas Gerais (CAAE 68351023.6.0000.5149), and the GeNED.ar–Argentina protocol was approved by the Research Ethics Committee of Hospital El Cruce (HEC) (D00079). All participants or their legally authorised representatives (in case of participants with cognitive impairment) provided written informed consent prior to study participation. Participants received no financial compensation for participation.

### Participants

Cog-Aging-Study–Brazil. The Cog-Aging-Study is a longitudinal cohort established at the Health Reference Centre for Older Adults, University Hospital, Federal University of Minas Gerais (Belo Horizonte, Brazil). A total of 353 individuals aged ≥60 years were enrolled through community-based senior socialization groups and referrals from the Geriatric Service of the University Hospital, and were followed annually. All participants underwent standardised clinical, cognitive, and functional evaluations conducted by trained geriatric specialists. Final clinical diagnoses (cognitively unimpaired [CU], mild cognitive impairment [MCI], dementia of the Alzheimer’s type [DAT]) were determined by multidisciplinary consensus. CSF and/or plasma samples were available for all participants, although availability differed according to recruitment and sampling pathways. Of these, 49 had only CSF, 239 only plasma, and 65 both. Full recruitment procedures, inclusion/exclusion criteria, neuropsychological instruments, and diagnostic procedures are detailed in the Supplementary Material.

GeNED.ar–Argentina. The GeNED.ar study (Genetics and Neuroimaging of Aging and Dementia in Argentina) is an ongoing study based at Hospital El Cruce/ENyS-CONICET, National University Arturo Jauretche (Florencio Varela, Buenos Aires, Argentina). Individuals aged ≥60 years were recruited from the community and a memory clinic. All participants underwent standardised clinical, cognitive, and functional assessments, as well as brain MRI, with final diagnoses (CU, MCI, DAT) assigned by multidisciplinary consensus. A total of 134 participants were included, all with available plasma samples. Full recruitment procedures, inclusion/exclusion criteria, and neuropsychological instruments are detailed in the Supplementary Material.

### Sample collection

CSF samples (Cog-Aging-Study–Brazil) were collected by lumbar puncture at the L3-4 or L4-5 interspace using sterile technique and 15 mL collection tubes. To minimise circadian variability in biomarker levels, procedures were performed at 10 AM. Samples were centrifuged within 2 h at 3000 *g* for 10 min at 4 °C, aliquoted into low-protein-binding tubes (Sarstedt SafeSeal reaction tube, 1.5 mL, Germany) and stored at −80 °C. Blood was collected in both cohorts in EDTA tubes using sterile technique, and within 2 hours, plasma was separated by centrifugation at 3000 *g* for 15 min at room temperature, aliquoted into low-protein-binding tubes (Cog-Aging-Study–Brazil: Sarstedt SafeSeal reaction tube 1.5 mL, Germany; GeNED.ar: Sigma-Aldrich Eppendorf® DNA LoBind 1.5 mL, USA), and stored at −80 °C.

### DNA analysis

DNA was isolated from whole blood samples using the QIAmp DNA mini kit (Qiagen) following the manufacturer’s recommendations. *APOE* was genotyped using TaqMan technology, assay IDs 3084793_20 (SNP rs429358) and 904973_10 (SNP rs7412). Single nucleotide polymorphisms (SNP) genotyping was performed using the Illumina Global Screening Array. Standard quality control (QC), imputation, and ancestry estimation were conducted using established pipelines (PLINK, TOPMed imputation, ADMIXTURE). After QC, 148 Cog-Aging-Study–Brazil and 126 GeNED.ar–Argentina samples were retained for genetic analyses. Full QC procedures, imputation details, and ancestry estimation methods are provided in the Supplementary Material.

### Biomarker measurement

CSF Aβ40, Aβ42, and pTau181 were measured using the Luminex xMAP platform with the Human Aβ and Tau Magnetic Bead Panel kit (HNABTMAG-68K, Millipore, Germany) at the Flow Cytometry Core Facility of FIOCRUZ-Minas, Belo Horizonte, Brazil. Plasma Aβ42, NfL, and GFAP were measured using the Neurology 4-plex E Simoa assay kit (Quanterix Billerica, USA), and pTau217 with the ALZpath V2 Simoa assay kit (Quanterix Billerica, USA) on a Simoa HD-X platform at the DZNE Bonn, Germany. Plasma biomarkers were run as singlicates by a researcher blinded to the clinical data. Calibrators were run in duplicates. Repeatability and intermediate precision were assessed using low- and high-concentration quality control samples. In Cog-Aging-Study–Brazil samples, coefficients of variation (%CV) ranged from 6.3 to 6.9% (repeatability) and 6.0–6.2% (intermediate precision) for pTau217, 2.6–3.9% and 2.0–3.2% for Aβ42, 9.9–13.1% and 6.7–8.0% for NfL, and 6.5–7.0% and 6.5–7.2% for GFAP, 5.7–10.2%, respectively. In GeNED.ar–Argentina samples, %CV ranged from 3.5–5.2% and 6.4–5.2% for pTau217, 1.9–4.1% and 2.8–4.3% for Aβ42, 2.5–6.1% and 4.6–5.7% for NfL, and 2.2–7.3% and 3.1–6.4% for GFAP.

### Statistics & reproducibility

Normality of biomarker distributions was assessed using visual inspection (Q-Q plots, histograms). Variables showing deviations from normality were log-transformed to reduce skewness and improve distributional properties for subsequent analyses, including Gaussian mixture modelling (GMM) and ANCOVA. Outliers were removed using the empirical rule (mean ± 3 SD).

Associations between biomarkers and sex were assessed using linear regression models. Associations with age were evaluated using Pearson correlation coefficients, both in the overall sample and within each clinical diagnostic group (CU, MCI, DAT). Differences in biomarker levels across diagnostic groups were examined using ANCOVA adjusted for age and sex, followed by Tukey post hoc tests for pairwise comparisons. Associations between biomarkers and cognitive and functional measures (CERAD EVOC, FAQ, and CDR sum of boxes) were assessed using linear regression models adjusted for age and sex. Differences in biomarker levels across levels of the ordinal CDR global score were examined using ANCOVA adjusted for age and sex, followed by Tukey post hoc tests.

Data-driven cut-offs in each of the studies or in the pooled dataset were determined using GMM implemented in the R package *mclust*, including all participants. Models with one to three latent classes were fitted, allowing also for varying variance. Model selection was based on the Bayesian Information Criterion (BIC), defined in *mclust* as: ${{{\rm{BIC}}}}=2\times l\left(\hat{\theta }\right)-m\times \log (n)$, where $l\left(\hat{\theta }\right)$ is the maximised log-likelihood, *m* the number of estimated parameters, and *n* the sample size^32^. In this formulation, models with higher BIC values are preferred. In cases of similar BIC values, the more parsimonious model was selected. In case of equal variances, the separation between latent classes was assessed using the overlapping coefficient (OVL), calculated with the formula for Gaussian distributions with equal variances: ${{{\rm{OVL}}}}=2\varphi (-\frac{\left|\mu 1-\,\mu 2\right|}{2\sigma })$, where *φ* is the standard normal distribution function, *μ*_*1*_ and *μ*_*2*_ the means of the two latent class distributions, and *σ* the common standard deviation^33^. Single cut-offs were defined as the intersection point of the two density functions. For the two-cut-off approach, lower and upper thresholds were determined as the boundaries of the “grey” or “indeterminate” zone, corresponding to posterior probabilities of <0.80, <0.85, or <0.90 for class assignment. Confidence intervals (95%) for positive predictive value (PPV), negative predictive value (NPV), sensitivity, and specificity were obtained via bootstrapping (10 000 replications) using the R package *bootLR*.

Clinically-based cut-offs in Cog-Aging-Study–Brazil were derived by including only CU and DAT participants. Logistic regression models were fitted with biomarker level as predictor and DAT status as the outcome. Cut-offs were defined using the Youden’s index, i.e., the threshold providing optimal trade-off between sensitivity and specificity on the receiver operating characteristic curve (ROC) implemented in the R package *pROC*.

The association between plasma biomarkers and each health or clinical-related covariate was examined using separate regression models: linear (continuous covariate) or logistic (dichotomous covariate) regression models, with covariates as predictors and biomarker levels as outcomes. The impact of global African ancestry on the association between *APOE-ε4* allele and biomarker levels was evaluated with the interaction term *APOE-ε4*×proportion of global African ancestry as predictor. All models were run both unadjusted and adjusted for age and sex. Continuous covariates were *z*-transformed to enable comparison of effect sizes across variables.

No statistical method was used to predetermine sample size. Outliers were excluded according to the prespecified criteria described above; otherwise, all available samples meeting the inclusion criteria were included in the analyses. Datasets were de-identified. The study was observational and therefore did not involve randomisation or blinding. Sex was self-reported. All analyses were conducted using R version 4.6.0.

### Reporting summary

Further information on research design is available in the Nature Portfolio Reporting Summary linked to this article.

## Supplementary information

Supplementary Information

Reporting Summary

Transparent Peer Review file

## Data Availability

The data generated in this study are available under restricted access due to ethical and legal restrictions related to the protection of participants’ clinical, cognitive, and genetic information, in accordance with the terms of the informed consent and the approval granted by the local Research Ethics Committees. Access to these data requires prior authorisation from the local Research Ethics Committees. For data relating to the Cog-Aging-Study, access requests should be sent by email to Professor Maria Aparecida Camargos-Bicalho (macbicalho@ufmg.br), who is responsible for the study. Requests will be reviewed and a response provided within 30 days. Access, when granted, will be provided for a period of 12 months following approval, restricted to the specific purpose stated in the request and to researchers who agree to comply with the terms established by the local Ethics Committee. The processed data required to interpret, verify, and extend the findings reported in this article can be made available for academic, non-commercial research purposes only. Access to the GeNED.ar data is available upon request. Data access requests should be submitted by email to Dr. Maria Carolina Dalmasso (mcdalmasso@unaj.edu.ar) at the Studies in Neuroscience and Complex Systems unite (ENyS-CONICET-HEC-UNAJ). Requests will be reviewed, and a response will be provided within 30 days. If approved, access will be granted for a period of six months and will be limited to the specific research purpose described in the request. Access will be restricted to researchers who agree to comply with the conditions established by the local Research Ethics Committee. The processed data necessary to interpret, verify, and extend the findings reported in this article will be made available exclusively for academic, non-commercial research purposes.

## Code availability

No custom code was developed specifically for this study.
