# Supplementary material for: Diagnostic performance of plasma pTau217 in genetically admixed South American populations
Source: Nat Commun. 2026 Aug 6;17:9464. doi: 10.1038/s41467-026-76434-2 (PMC13542151; doi:10.1038/s41467-026-76434-2)
Supplement: Supplementary file 1 [file 41467_2026_76434_MOESM1_ESM.pdf]

Supplementary Information

Supplementary Methods

Participants

Cog-Aging-Study–Brazil. Cog-Aging-Study is a longitudinal cohort established at the Health Reference Centre for Older Adults, University Hospital, Universidade Federal de Minas Gerais (UFMG), Belo Horizonte, Brazil. Recruitment was conducted through community-based senior socialization groups and referrals from the Geriatric Service of the University Hospital, which receives older adults referred by primary healthcare centers from across the city of Belo Horizonte. Eligible participants were ≥60 years of age and native Portuguese speakers. Exclusion criteria were cognitive impairment due to non-Alzheimer's dementia, severe major depressive or other psychiatric disorders, hydrocephalus, intracranial mass, history of head trauma, Parkinson's disease, active neoplasm, severe frailty, significant mobility limitations, serious visual or hearing impairment, delirium, active inflammatory disease, or ongoing oncologic treatment.

A total of 353 participants with varying levels of cognitive performance were enrolled and followed annually. All participants underwent a comprehensive and standardised research protocol, including clinical and neuropsychological evaluations conducted by a geriatrician and/or a geriatric psychiatrist. Cognitive assessments included the Mini-Mental State Examination (MMSE), Consortium to Establish a Registry for Alzheimer's Disease (CERAD) battery, and the Frontal Assessment Battery (FAB). Functional status was evaluated using the Functional Activities Questionnaire (FAQ) and the Clinical Dementia Rating (CDR) scale. The neuropsychological battery used in this study, previously validated for the assessment of cognitive performance in Brazilian populations with comparable characteristics[1], included Mattis Dementia Rating Scale, Rey Auditory Verbal Learning Test (RAVLT), Stick Design Test (SDT), Five Digit Test (FDT), TNLIN, Verbal Fluency Test, Wechsler Adult Intelligence Scale/Wechsler Abbreviated Scale of Intelligence (WAIS/WASI), Tower of London Test (TOL), and Digit Span. Diagnoses of mild cognitive impairment (MCI) and dementia were established according to DSM-5 criteria[2], and diagnosis of dementia of the Alzheimer's type (DAT) was based on McKhann et al[3]. Biochemical, hematologic, and serologic analyses were performed to assess potential aetiologies and comorbidities, and neuroimaging was conducted at baseline and repeated whenever cognitive status changed. Final clinical diagnoses were determined in a consensus meeting that integrated medical, neuropsychological, laboratory, and neuroimaging data. All participants or family members (in case of participants with cognitive impairment) signed informed consent forms previously approved by the Local Ethics Committee (CAAE 68351023.6.0000.5149).

All participants had available CSF and/or plasma samples. Differences in sample composition across these groups reflect distinct recruitment and sampling pathways. In particular, CSF collection was performed both in participants recruited through the cognitive outpatient clinic and in individuals undergoing procedures requiring spinal anaesthesia, whereas plasma samples were obtained more broadly within the cohort. Consequently, availability of CSF and paired samples was influenced by recruitment pathway, clinical indication, and logistical/ethical constraints. Of these, 49 participants had only CSF samples available (cognitively unimpaired [CU], n=20; MCI, n=10; DAT, n=16; missing diagnosis, n=3), 239 had only plasma samples (CU, n=83; MCI, n=109; DAT, n=47), 45 had paired CSF-plasma samples collected within a 12-month interval, and 20 had unpaired CSF and plasma samples collected more than 12 months apart. For participants with both CSF and plasma samples, clinical diagnosis occasionally differed between the two collection time points, reflecting diagnostic updates or disease progression over time. This occurred both among participants with paired samples and those with samples collected more than 12 months apart. For analyses involving CSF biomarkers, diagnostic classification was based on the clinical diagnosis at the time of CSF collection; for analyses involving plasma biomarkers, the diagnosis at the plasma collection time point was used. Analyses requiring both CSF and plasma measures included only participants with paired samples (≤12-month interval), whereas individuals with unpaired samples were included exclusively in the CSF or plasma analyses, as appropriate.

GeNED.ar–Argentina. The GeNED.ar study (Genetics and Neuroimaging of Aging and Dementia in Argentina) is an ongoing study conducted at the Studies in Neuroscience and Complex Systems Unit (ENyS-CONICET) from Hospital El Cruce (HEC), National University Arturo Jauretche (UNAJ) in Florencio Varela, Buenos Aires, Argentina (https://enys.conicet.gov.ar/genedar/). Recruitment is carried out both in the general population and through a memory clinic. All participants (aged ≥60 years) undergo clinical, neuropsychological, and behavioural assessments, routine blood tests, and brain MRI scans. Results are reviewed in a weekly meeting integrated by physicians and neuropsychologists, where clinical diagnoses are reached by consensus following the criteria described by McKhann et al[3].

Neuropsychological assessments include the Addenbrooke's Cognitive Examination Revised (ACE-R), MMSE, CDR, FAB, FAQ, Global Deteriorating Scale (GDS), and the Neuropsychiatric Inventory Questionnaire (NPI-Q). The study was approved by the HEC Research Ethics Committee (D00079), and all participants and/or family members provided written informed consent. A total of 134 participants were included in this study (CU, n=81 MCI, n=35; DAT, n=18), and all participants had available plasma samples. Participants with non-Alzheimer's dementia, other psychiatric disorders, history of head trauma, and/or cancer were excluded.

DNA analysis

Single nucleotide polymorphisms (SNPs) were genotyped using the Illumina Infinium Global Screening Array (GSA v1.0 with shared custom content and GSA-24 v3.0, for Cog-Aging-Study–Brazil and GeNED.ar–Argentina, respectively). Quality control (QC) and imputation were performed using PLINK 1.9 (https://www.cog-genomics.org/plink/), as previously described[4–6]. Briefly, samples with a call rate below 98%, excess of heterozygosity, sex discrepancies, duplicates, or evidence of relatedness (PI-HAT>0.2) were excluded from the analysis. Variants with a call rate below 97%, a deviation from the Hardy-Weinberg equilibrium (HWE, $p<1\times10\text{-}6$), or differential missingness between cases and controls were also removed. After QC, 148 samples from Cog-Aging-Study–Brazil and 126 samples from GeNED.ar–Argentina remained. To maximise genetic coverage, SNP imputation was performed on genome build GRCh38 using the Trans-Omics for Precision Medicine (TOPMed) imputation server[7–9]. Global ancestry was estimated as previously described[5,6]. Briefly, 356 autosomal Ancestry Informative Markers (AIMs), evenly distributed among chromosomes, were extracted from Cog-Aging-Study–Brazil and GeNED.ar–Argentina, as well as from reference populations in the 1000 Genomes Project (http://www.internationalgenome.org/): Europeans (CEU, n=85), Africans (YRI, n=88), and Native Americans (NAM, n=43). All datasets were merged in one PLINK v1.9 file (www.cog-genomics.org/plink/1.9/), and global ancestry was estimated using ADMIXTURE v1.3.0[10].

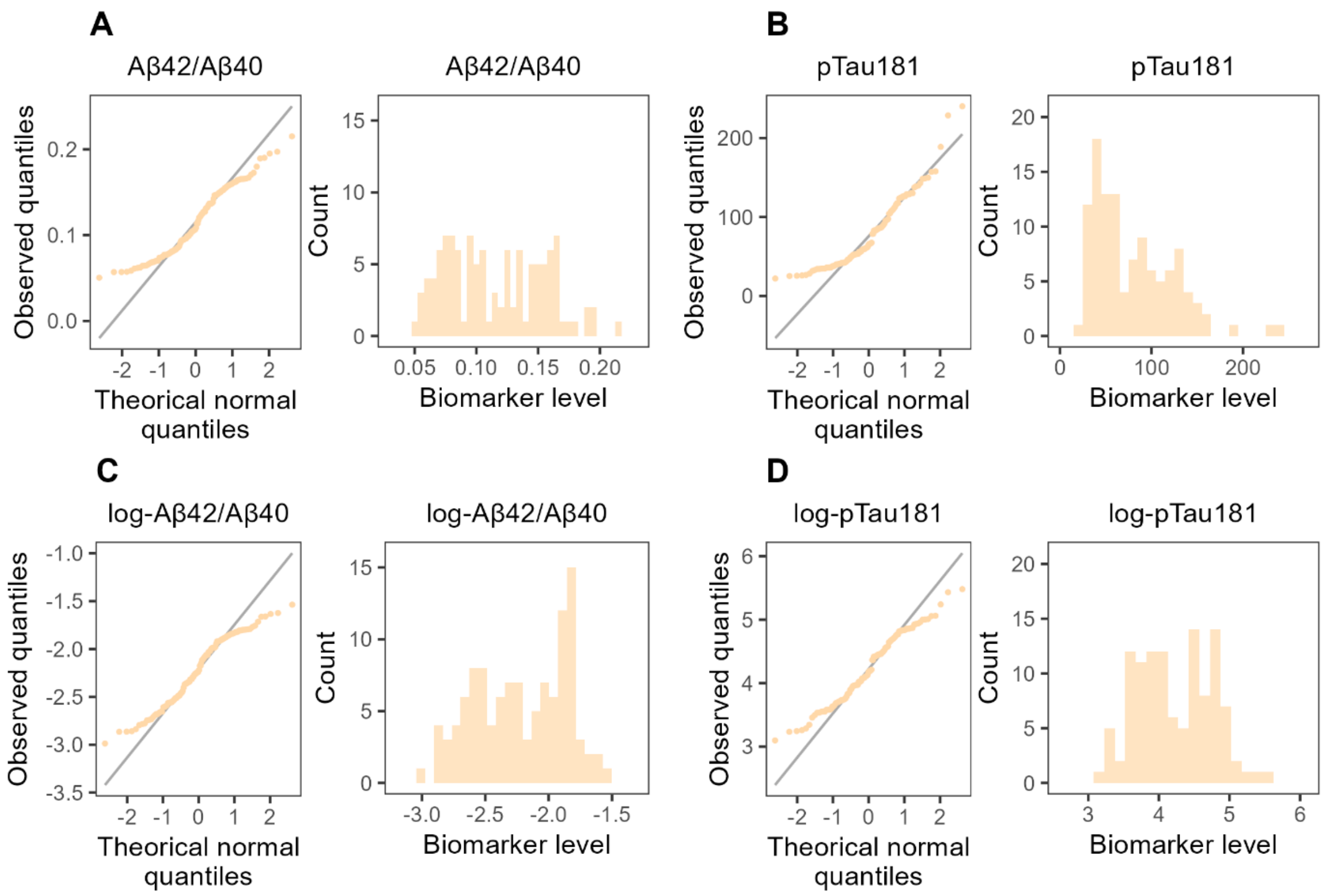

Supplementary Figure 1. Distribution properties of CSF Aβ42/Aβ40 and pTau181 in Cog-Aging-Study–Brazil. Panels A-B show the distributions of CSF Aβ42/Aβ40 and pTau181 in the original scale (pg/mL), presented as QQ-plots (left) and histograms (right). Panels C-D show the corresponding distributions after log-transformation.

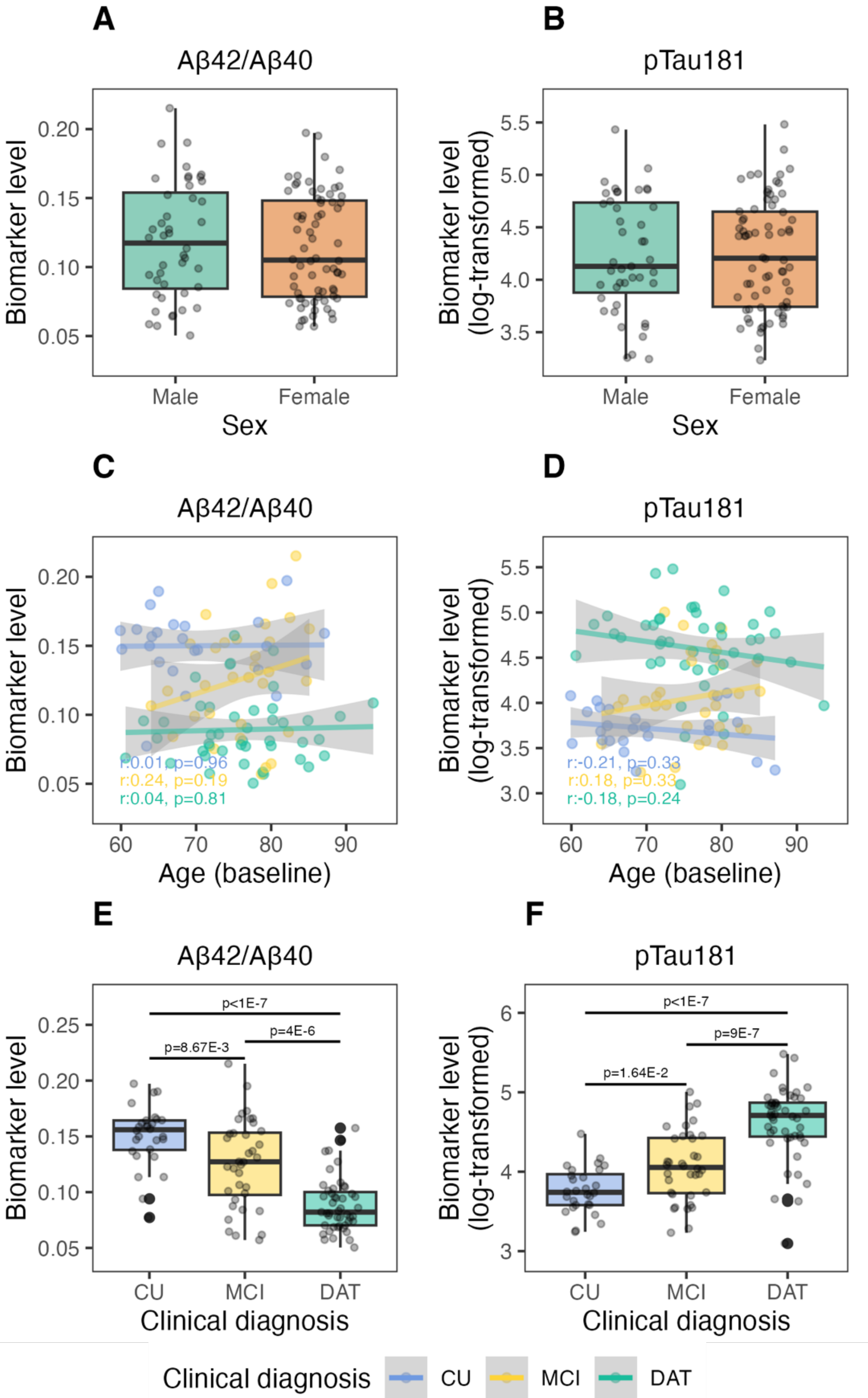

Supplementary Figure 2. Association of CSF Aβ42/Aβ40 and pTau181 with sex, age, and clinical diagnosis in Cog-Aging-Study–Brazil. Panels A-B show boxplots according to sex, panels C-D show scatter plots illustrating the correlation with age within each diagnostic group, and panels E-F show boxplots according to clinical diagnosis. Trend lines in panels C-D are shown for visualisation purposes only. pTau181 values were log-transformed. Association with categorical variables were evaluated using ANCOVA followed by Tukey's post-hoc test, and p-values are shown only for associations with $p<0.05$. CU, cognitively unimpaired; MCI, mild cognitive impairment; DAT, dementia of the Alzheimer's type; r, Pearson's correlation coefficient.

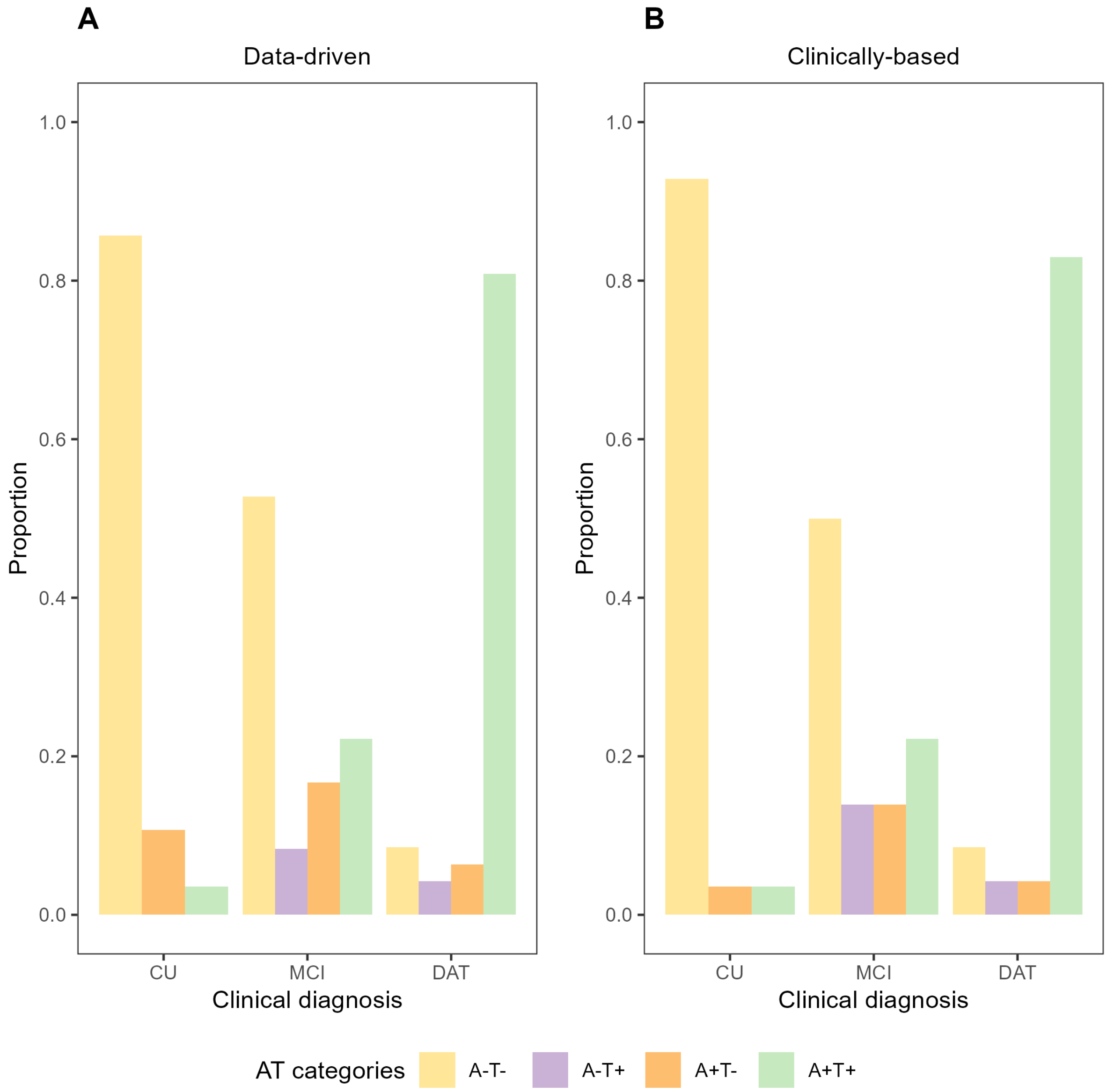

Supplementary Figure 3. AT groups across clinical diagnostic groups in Cog-Aging-Study–Brazil. Bar plots show the proportion of each AT group in each diagnostic group using either data-driven (A) or clinically-based (B) cut-offs for CSF Aβ42/Aβ40 and pTau181. CU, cognitively unimpaired (n=28); MCI, mild cognitive impairment (n=36); DAT, dementia of the Alzheimer's type (n=47).

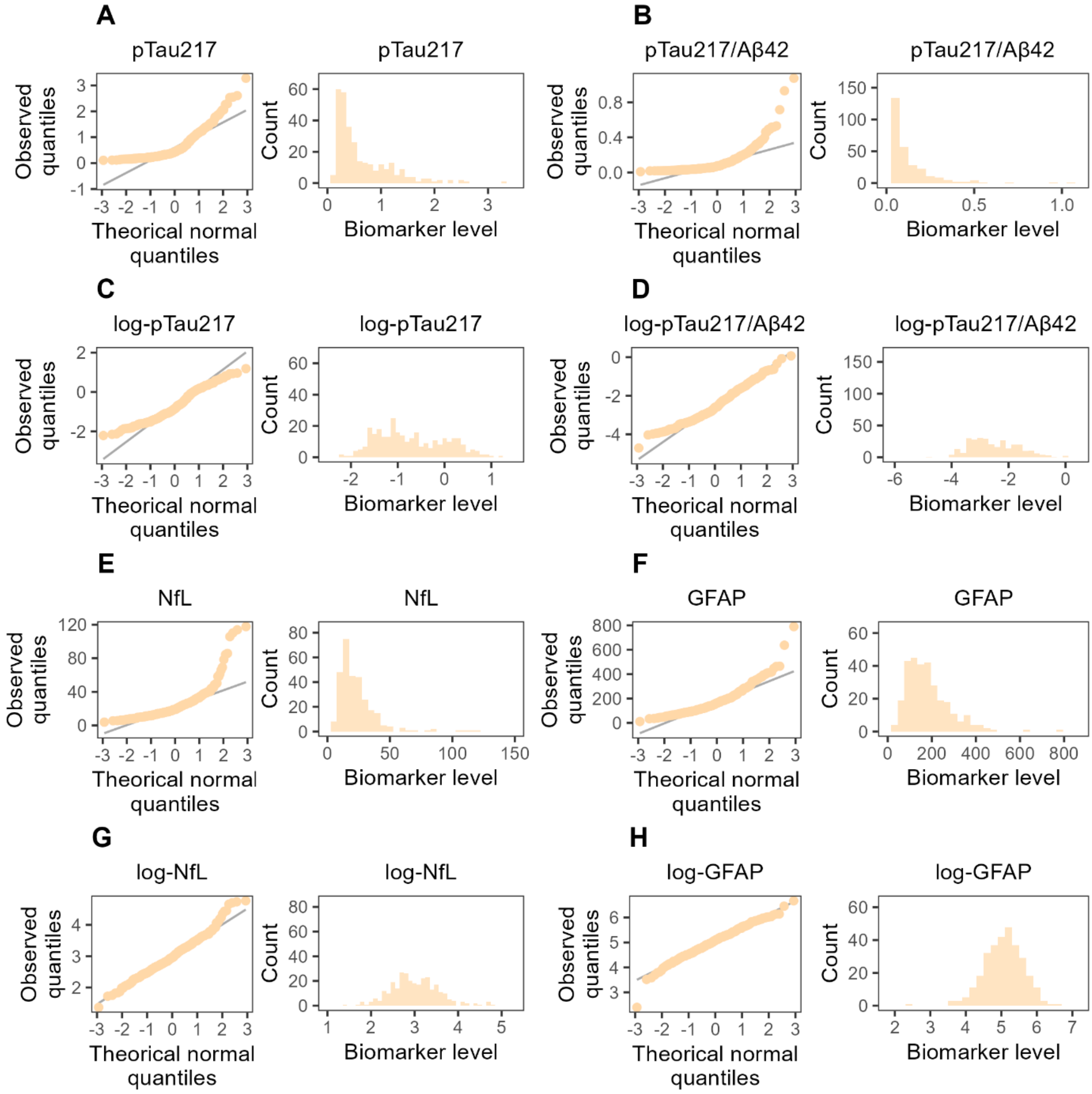

Supplementary Figure 4. Distribution properties of plasma pTau217, pTau217/Aβ42, NfL, and GFAP in Cog-Aging-Study–Brazil. Panels A-B and E-F show the distributions of plasma pTau217, pTau217/Aβ42, NfL, and GFAP in the original scale (pg/mL), presented as QQ-plots (left) and histograms (right). Panels C-D and G-H show the corresponding distributions after log-transformation.

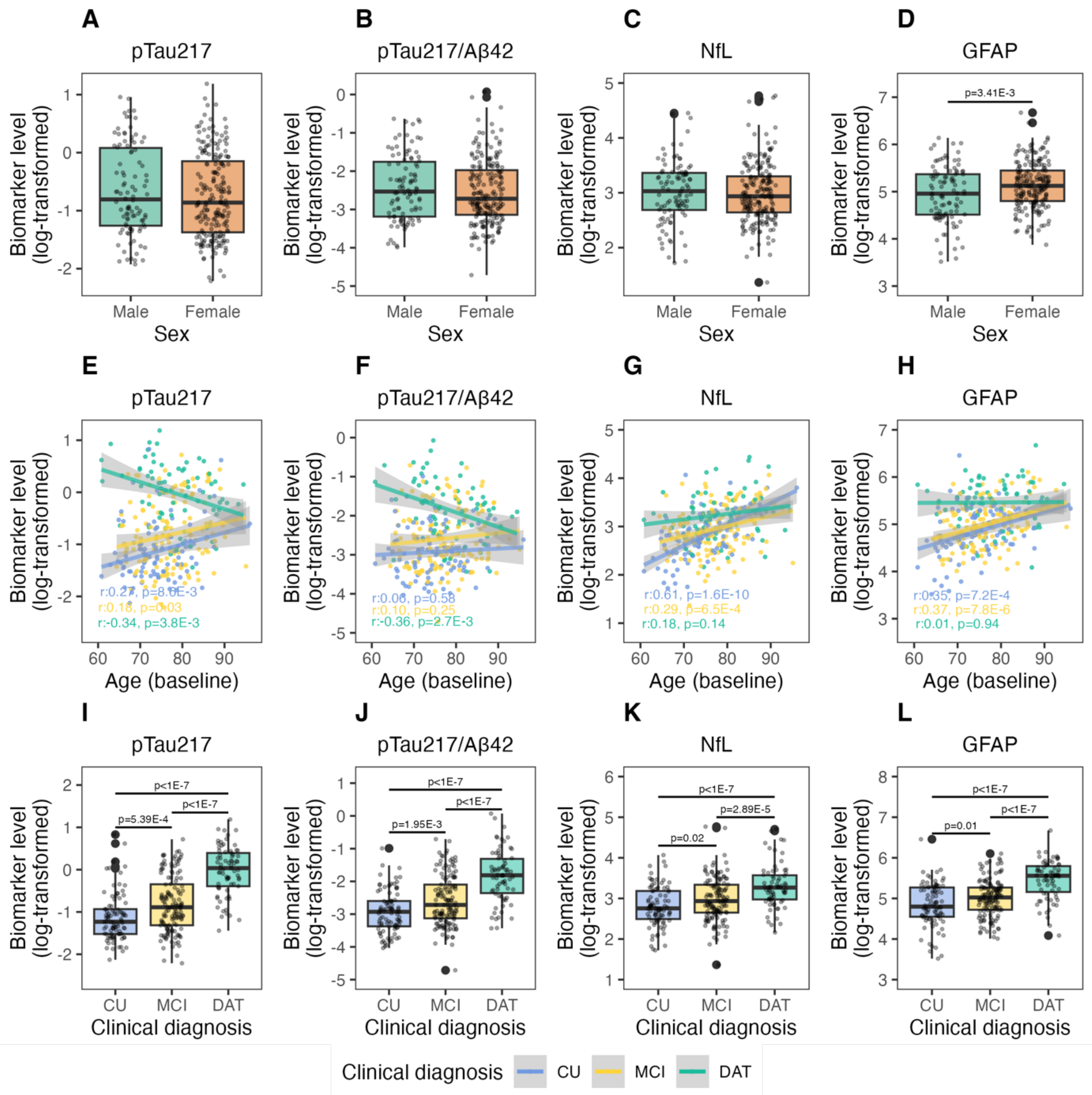

Supplementary Figure 5. Association of plasma pTau217, pTau217/Aβ42, NfL, and GFAP with sex, age, and clinical diagnosis in Cog-Aging-Study–Brazil. Panels A-D show boxplots according to sex, panels E-H show scatter plots illustrating the correlation with age within each diagnostic group, and panels I-L show boxplots according to clinical diagnosis. Trend lines in panels C-D are shown for visualisation purposes only. Biomarkers values were log-transformed. Associations in A-D and I-L were evaluated using ANCOVA followed by Tukey's post-hoc test, and p-values are shown only for associations with $p<0.05$. CU, cognitively unimpaired; MCI, mild cognitive impairment; DAT, dementia of the Alzheimer's type; r, Pearson's correlation coefficient.

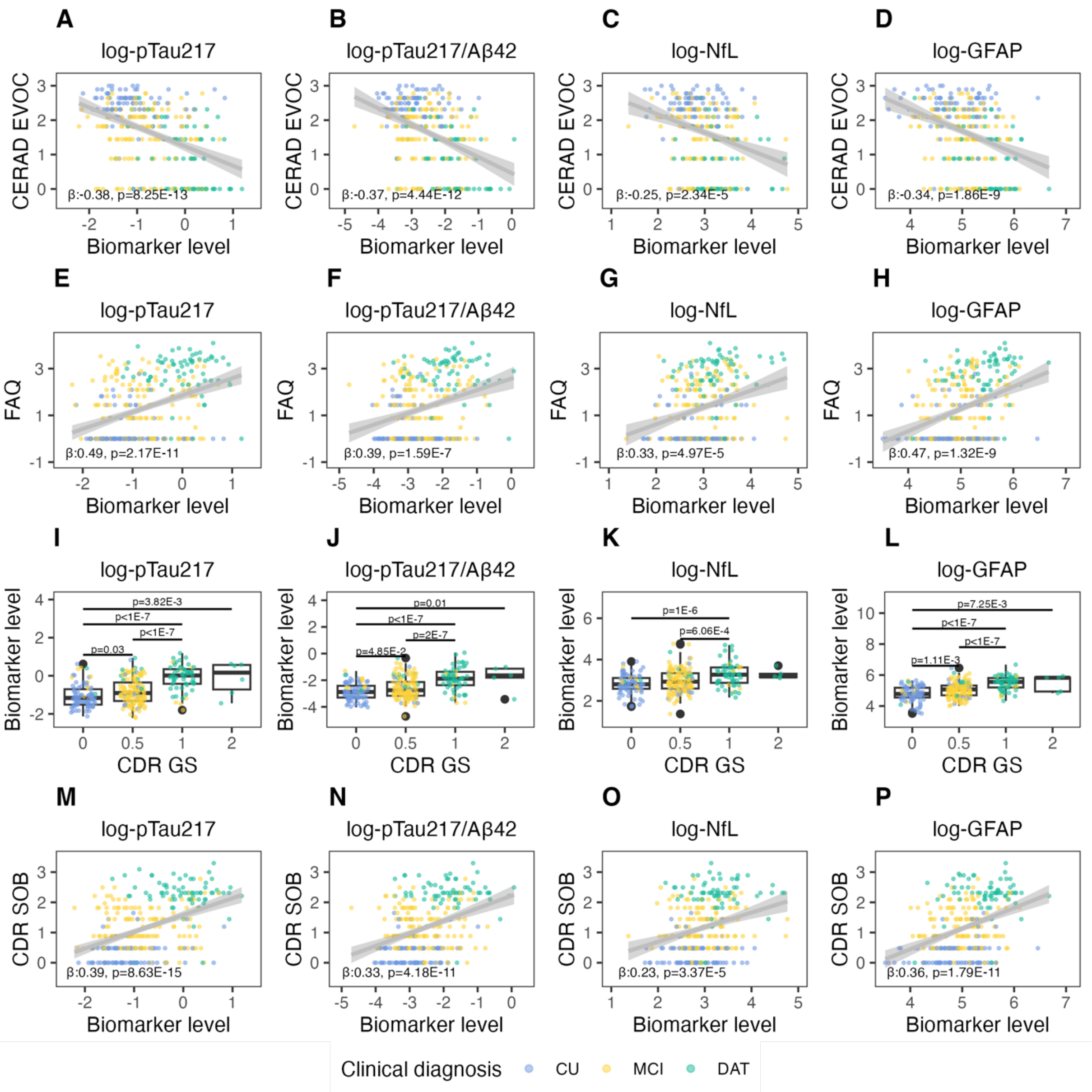

Supplementary Figure 6. Association of plasma pTau217, pTau217/Aβ42, NfL, and GFAP with cognitive and functional measures in Cog-Aging-Study–Brazil. Panels A-D show scatter plots illustrating the association with CERAD-EVOC (episodic memory) within each diagnostic group; panels E-H show scatter plots illustrating the association with FAQ (functional impairment) within each diagnostic group; panels I-L show boxplots according to CDR global score (overall dementia stage); and panels M-P show scatter plots illustrating the association with CDR sum of boxes (cognitive-functional impairment) within each diagnostic group. Trend lines are shown for visualisation purposes only. Biomarker values were log-transformed. All associations were evaluated using linear regressions (continuous variables) or ANCOVA followed by Tukey's post-hoc test (categorical variables) adjusted for age and sex. p-values in I-L are shown only for associations with $p<0.05$. CU, cognitively unimpaired; MCI, mild cognitive impairment; DAT, dementia of the Alzheimer's type; β, linear regression effect size.

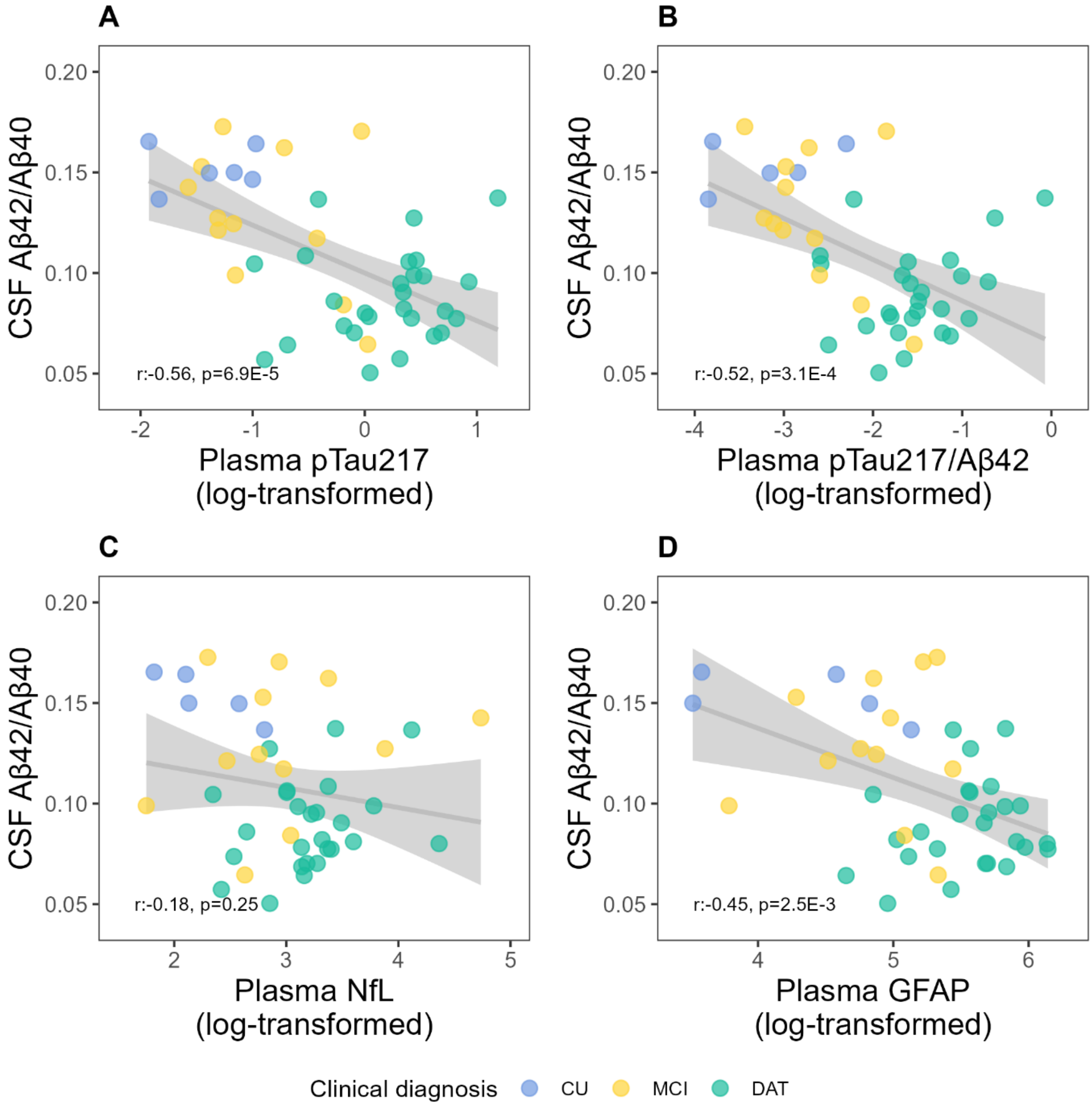

Supplementary Figure 7. Correlation between CSF Aβ42/Aβ40 and plasma biomarkers in Cog-Aging-Study–Brazil. Scatter plots show the correlation between CSF Aβ42/Aβ40 and plasma pTau217 (A), pTau217/Aβ42 (B), NfL (C), and GFAP (D). Trend lines are shown for visualisation purposes only. Plasma biomarker values were log-transformed. CU, cognitively unimpaired; MCI, mild cognitive impairment; DAT, dementia of the Alzheimer's type; r, Pearson's correlation coefficient.

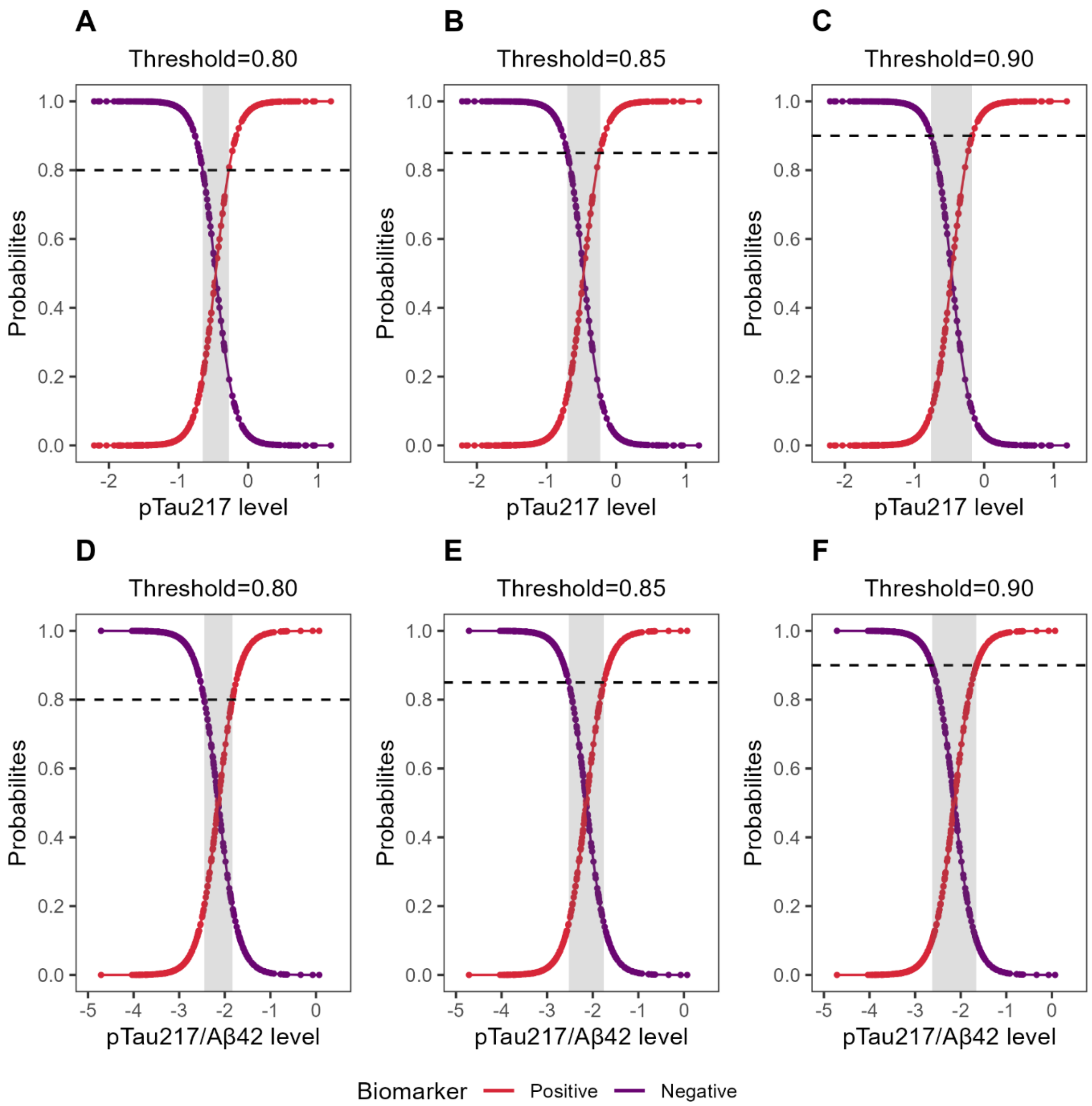

Supplementary Figure 8. Class membership probabilities and indeterminate ranges for plasma pTau217 and pTau217/Aβ42 in Cog-Aging-Study–Brazil. The purple line shows the probability of being classified as pTau217+ (A-C) or pTau217/Aβ42+ (D-F), and the red line shows the probability of being classified as pTau217- or pTau217/Aβ42- across biomarker levels. The grey shading indicates the range where classification was uncertain, defined by probability thresholds of <0.80 (A, D), <0.85 (B, E), or <0.90 (C, F). Plasma pTau217 and pTau217/Aβ42+ levels were log-transformed.

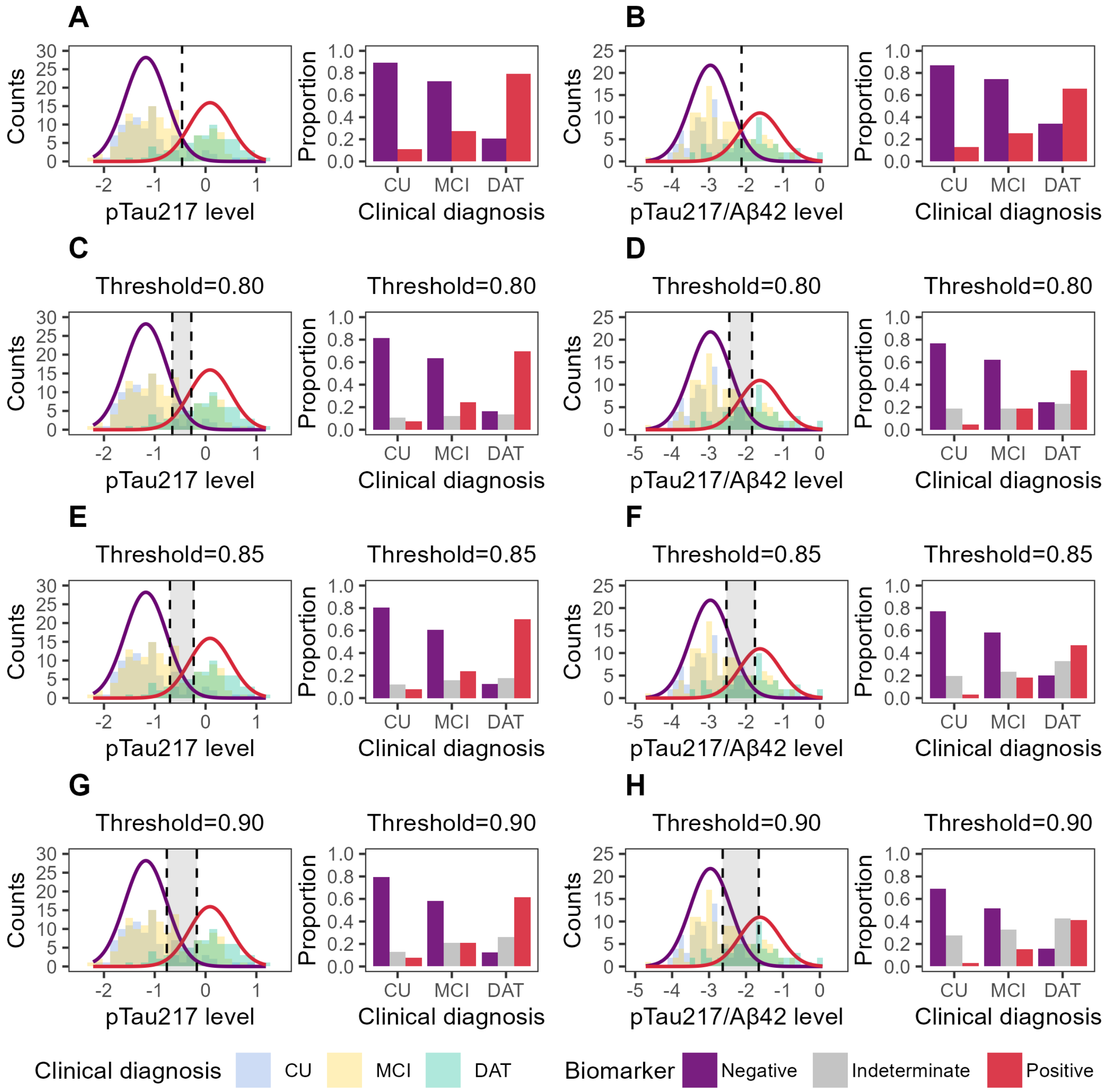

Supplementary Figure 9. Extended data-driven cut-offs for plasma pTau217 and pTau217/Aβ42 in Cog-Aging-Study–Brazil. This figure expands upon Figure 2. Histograms show the distribution of biomarker levels across clinical diagnostic groups, with overlaid curves representing the modelled distributions of normal and abnormal values. In panels A-B, values left of the dashed line indicate normal biomarker levels, and values to the right indicate abnormal levels. In panels C-H, the two dashed lines represent lower and upper cut-offs at probability thresholds of 0.80 (C-D), 0.85 (E-F), and 0.90 (G-H). Values left of the lower line are classified as normal, values right of the upper line as abnormal, and grey shading marks the intermediate range where status was uncertain. Bar plots display the proportion of each biomarker status (negative, indeterminate, positive) within diagnostic groups at each threshold. Plasma pTau217 and pTau217/Aβ42 values were log-transformed. CU, cognitively unimpaired; MCI, mild cognitive impairment; DAT, dementia of the Alzheimer's type.

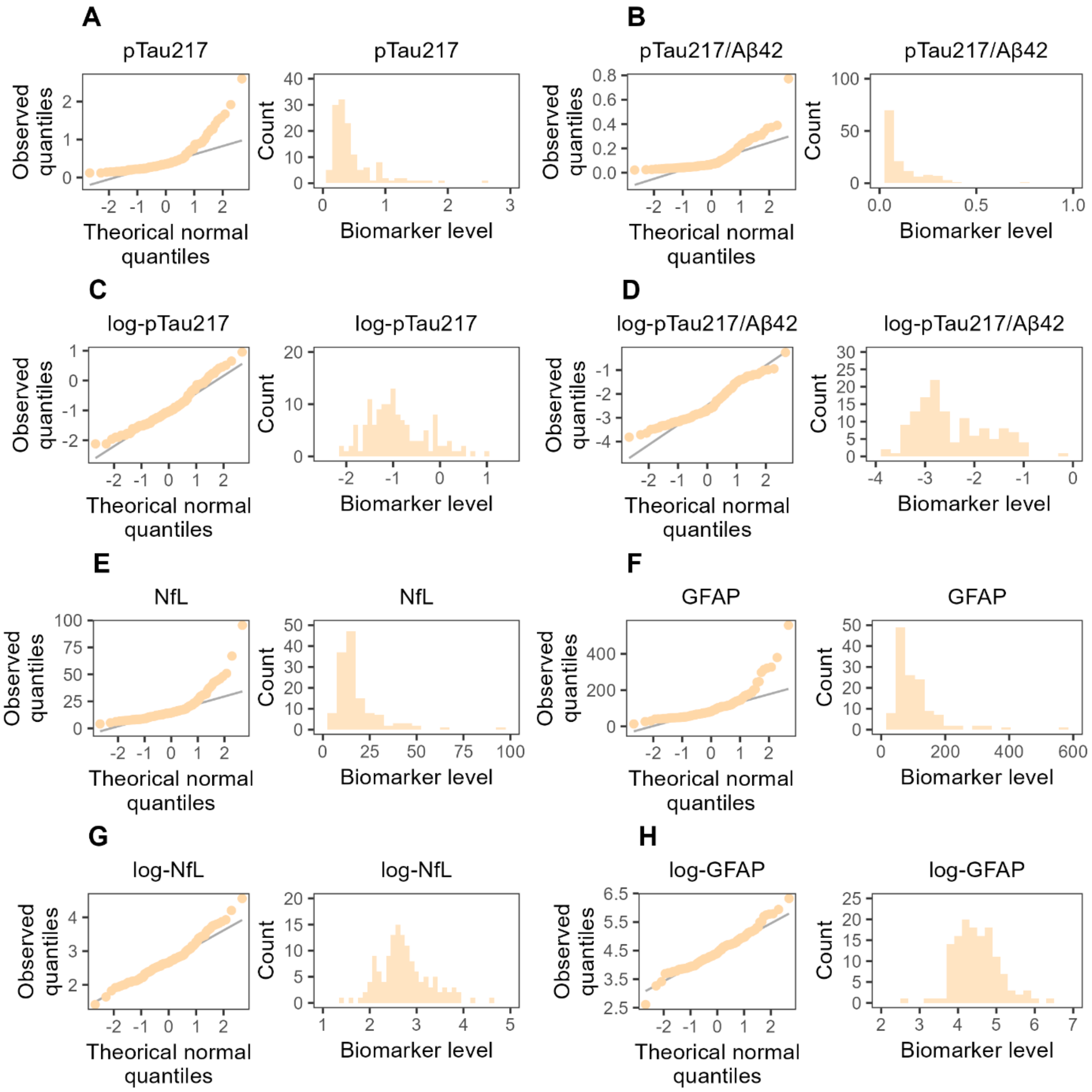

Supplementary Figure 10. Distribution properties of plasma pTau217, pTau217/Aβ42, NfL, and GFAP in GeNED.ar–Argentina. Panels A-B and E-F show the distributions of plasma pTau217, pTau217/Aβ42, NfL, and GFAP in the original scale (pg/mL), presented as QQ-plots (left) and histograms (right). Panels C-D and G-H show the corresponding distributions after log-transformation.

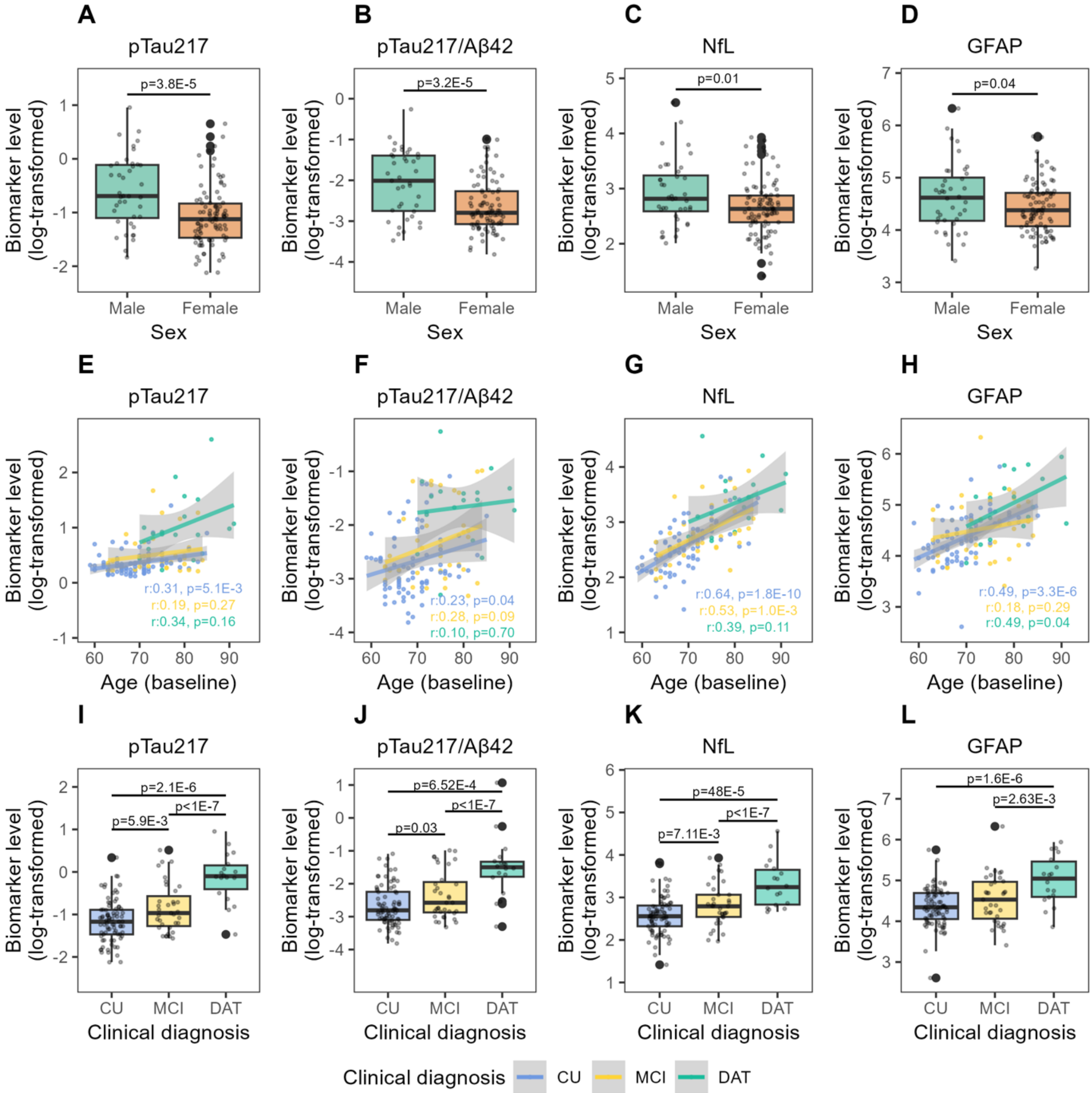

Supplementary Figure 11. Association of plasma pTau217, pTau217/Aβ42, NfL, and GFAP with sex, age, and clinical diagnosis in GeNED.ar–Argentina. Panels A-D show boxplots according to sex, panels E-H show scatter plots illustrating the correlation with age within each diagnostic group, and panels I-L show boxplots according to clinical diagnosis. Trend lines in panels C-D are shown for visualisation purposes only. Biomarkers values were log-transformed. Associations in A-D and I-L were evaluated using ANCOVA followed by Tukey's post-hoc test, and p-values are shown only for associations with $p<0.05$. CU, cognitively unimpaired; MCI, mild cognitive impairment; DAT, dementia of the Alzheimer's type; r, Pearson's correlation coefficient.

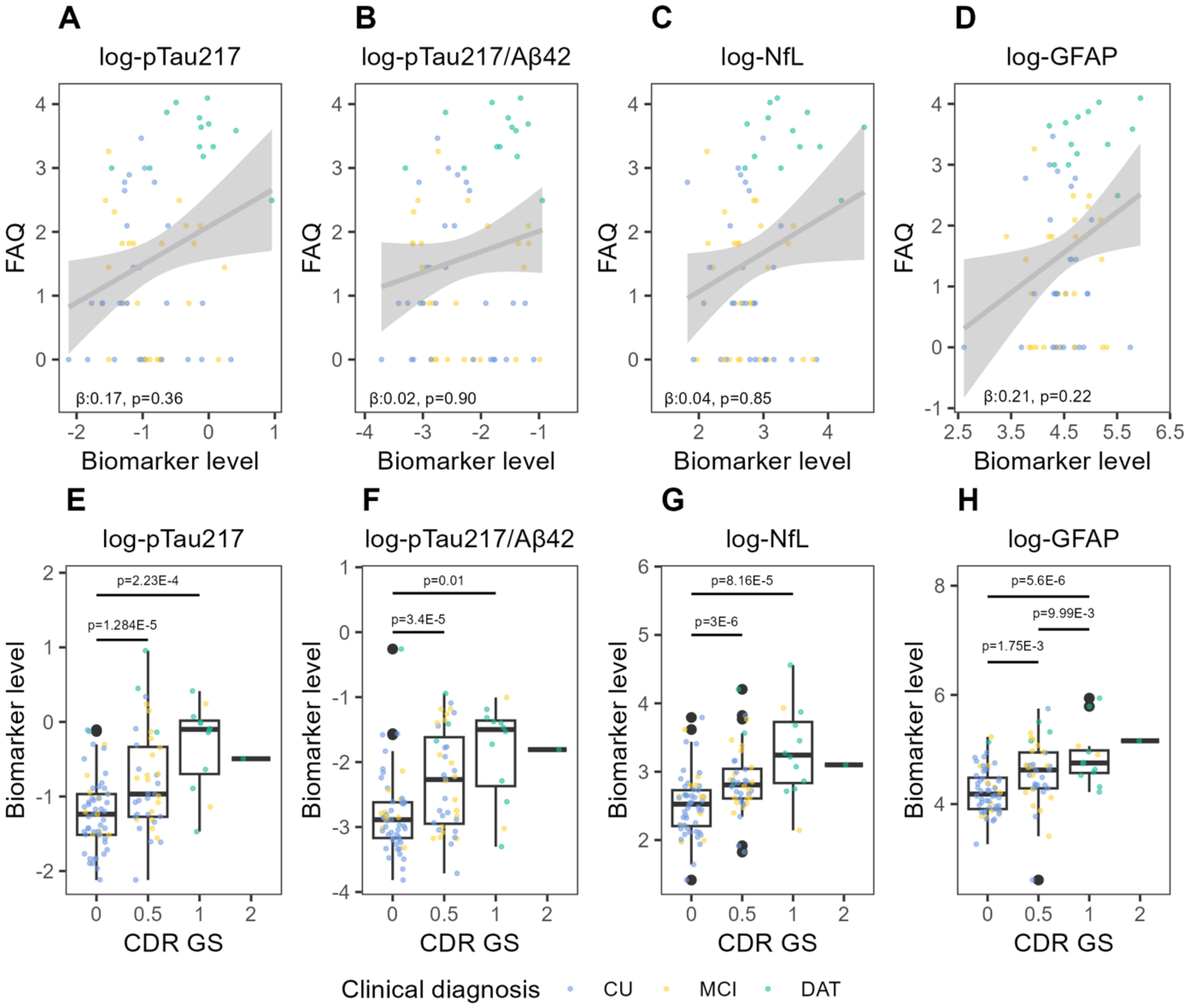

Supplementary Figure 12. Association of plasma pTau217, pTau217/Aβ42, NfL, and GFAP with cognitive and functional measures in GeNED.ar–Argentina. Panels A-D show scatter plots illustrating the association with FAQ (functional impairment) within each diagnostic group, and panels E-H show boxplots according to CDR global score (overall dementia stage). Trend lines are shown for visualisation purposes only. Biomarker values were log-transformed. All associations were evaluated using linear regressions (continuous variables) or ANCOVA followed by Tukey's post-hoc test (categorical variables) adjusted for age and sex. p-values in E-H are shown only for associations with $p<0.05$. CU, cognitively unimpaired; MCI, mild cognitive impairment; DAT, dementia of the Alzheimer's type; β, linear regression effect size.

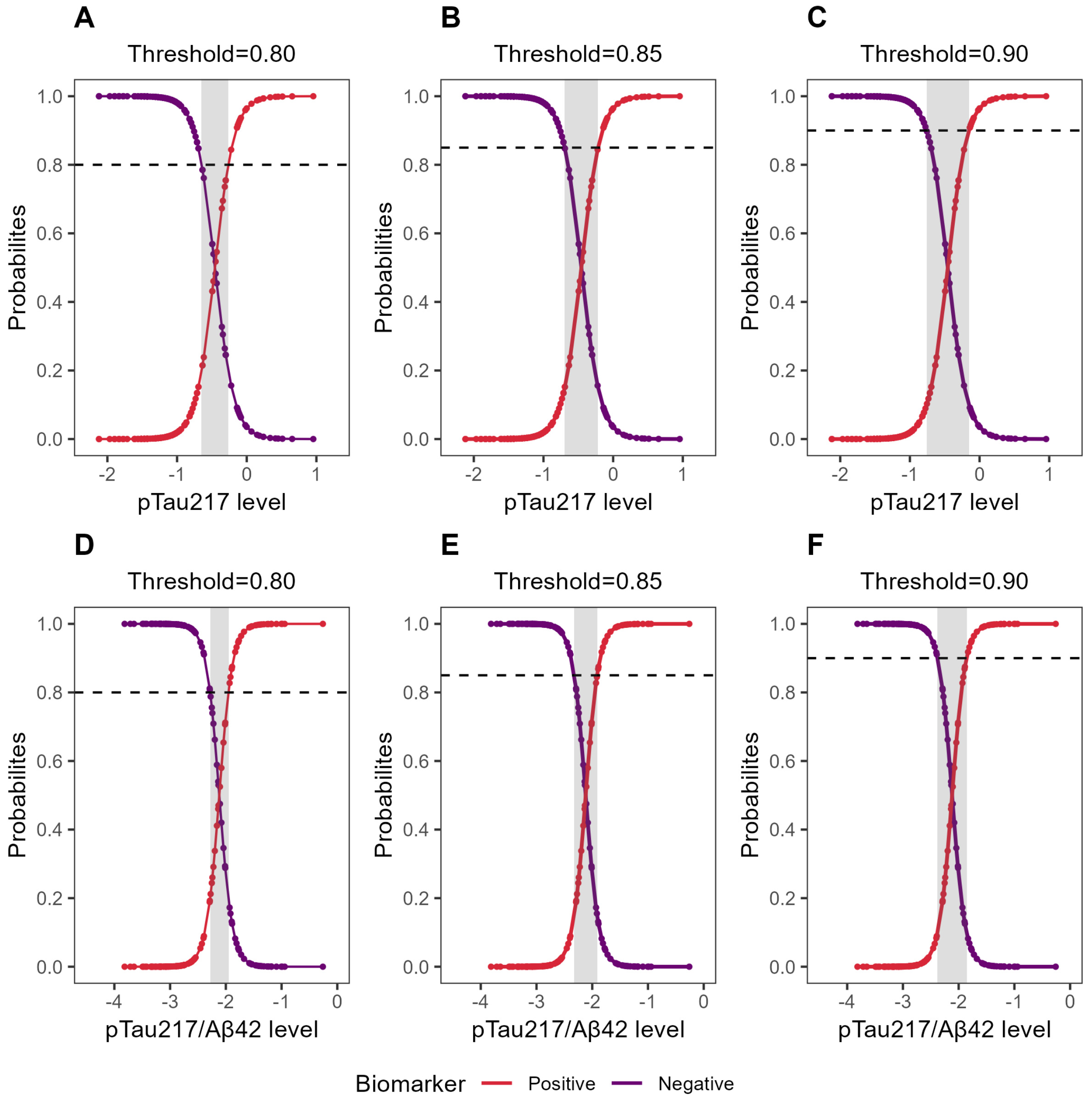

Supplementary Figure 13. Class membership probabilities and indeterminate ranges for plasma pTau217 and pTau217/Aβ42 in GeNED.ar–Argentina. The purple line shows the probability of being classified as pTau217+ (A-C) or pTau217/Aβ42+ (D-F), and the red line shows the probability of being classified as pTau217- or pTau217/Aβ42- across biomarker levels. The grey shading indicates the range where classification was uncertain, defined by probability thresholds of <0.80 (A, D), <0.85 (B, E), or <0.90 (C, F). Plasma pTau217 and pTau217/Aβ42+ levels were log-transformed.

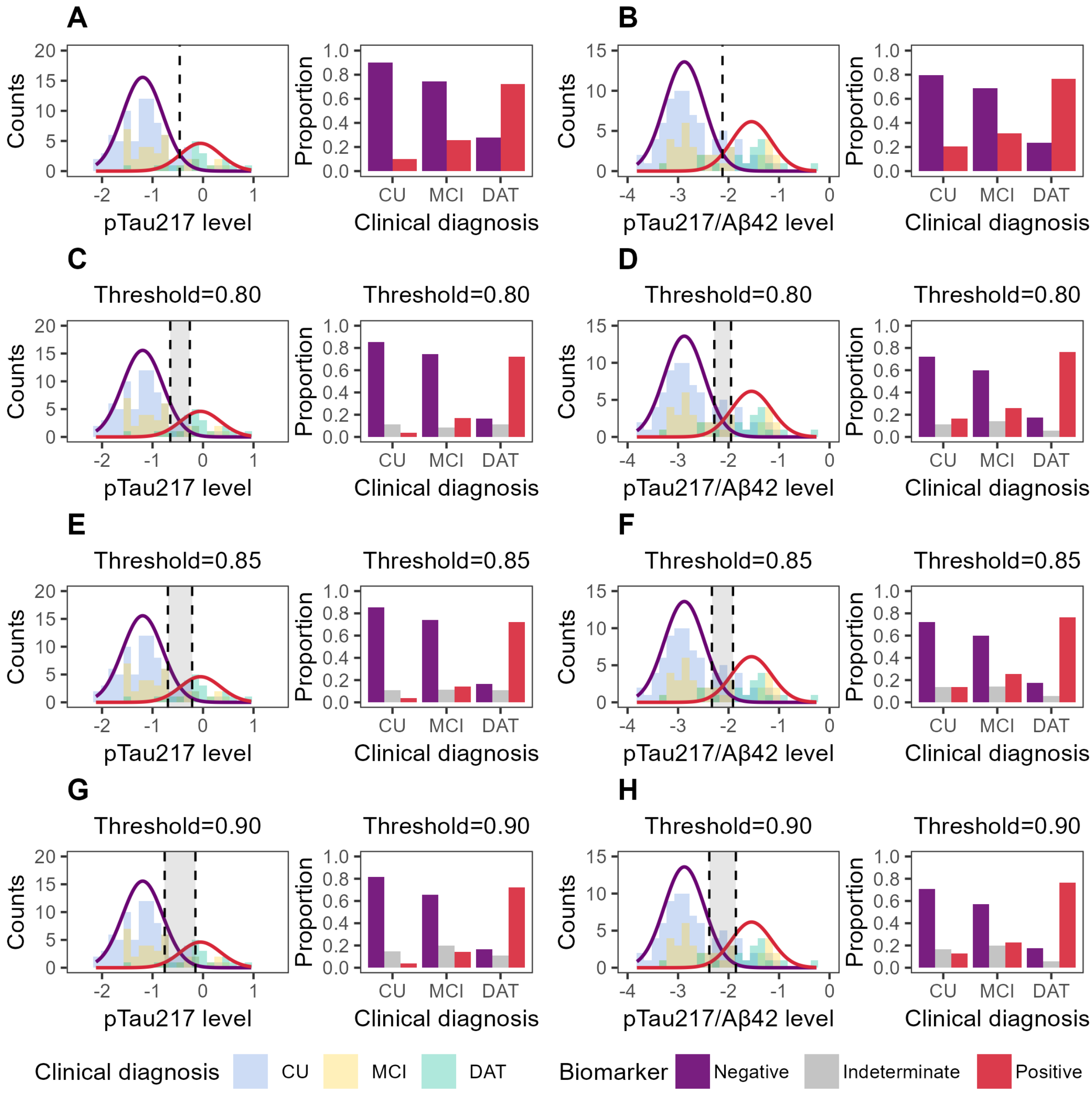

Supplementary Figure 14. Extended data-driven cut-offs for plasma pTau217 and pTau217/Aβ42 in GeNED.ar–Argentina. This figure expands upon Figure 2. Histograms show the distribution of biomarker levels across clinical diagnostic groups, with overlaid curves representing the modelled distributions of normal and abnormal values. In panels A-B, values left of the dashed line indicate normal biomarker levels, and values to the right indicate abnormal levels. In panels C-H, the two dashed lines represent lower and upper cut-offs at probability thresholds of 0.80 (C-D), 0.85 (E-F), and 0.90 (G-H). Values left of the lower line are classified as normal, values right of the upper line as abnormal, and grey shading marks the intermediate range where status was uncertain. Bar plots display the proportion of each biomarker status (negative, indeterminate, positive) within diagnostic groups at each threshold. Plasma pTau217 and pTau217/Aβ42 values were log-transformed. CU, cognitively unimpaired; MCI, mild cognitive impairment; DAT, dementia of the Alzheimer's type.

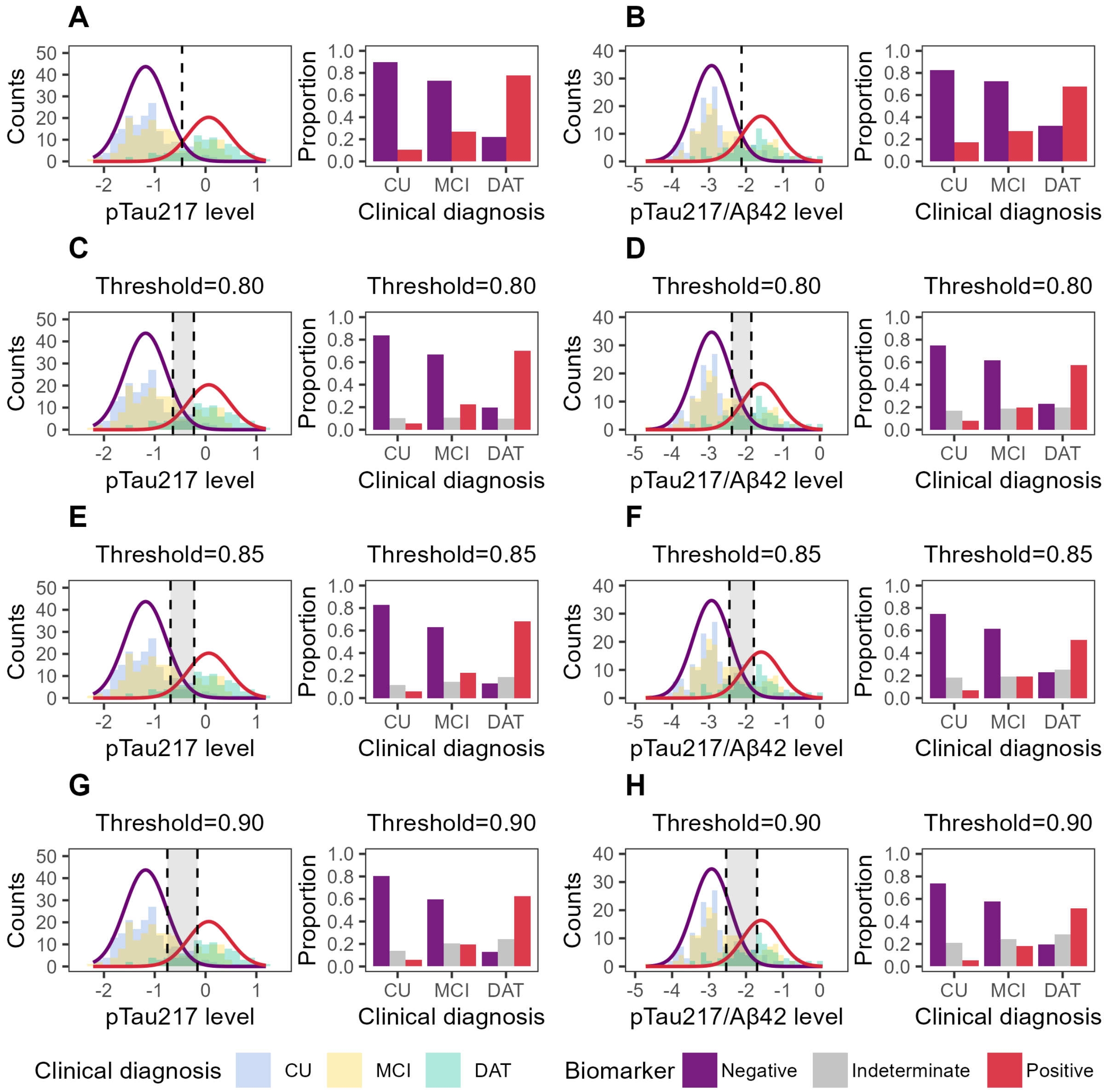

Supplementary Figure 15. Data-driven cut-offs for plasma pTau217 and pTau217/Aβ42 in the pooled Cog-Aging-Study–Brazil/GeNED.ar–Argentina dataset. Histograms show the distribution of biomarker levels across clinical diagnostic groups, with overlaid curves representing the modelled distributions of normal and abnormal values. In panels A-B, values left of the dashed line indicate normal biomarker levels, and values to the right indicate abnormal levels. In panels C-H, the two dashed lines represent lower and upper cut-offs at probability thresholds of 0.80 (C-D), 0.85 (E-F), and 0.90 (G-H). Values left of the lower line are classified as normal, values right of the upper line as abnormal, and grey shading marks the intermediate range where status was uncertain. Bar plots display the proportion of each biomarker status (negative, indeterminate, positive) within diagnostic groups at each threshold. Plasma pTau217 and pTau217/Aβ42 values were log-transformed. CU, cognitively unimpaired; MCI, mild cognitive impairment; DAT, dementia of the Alzheimer's type.

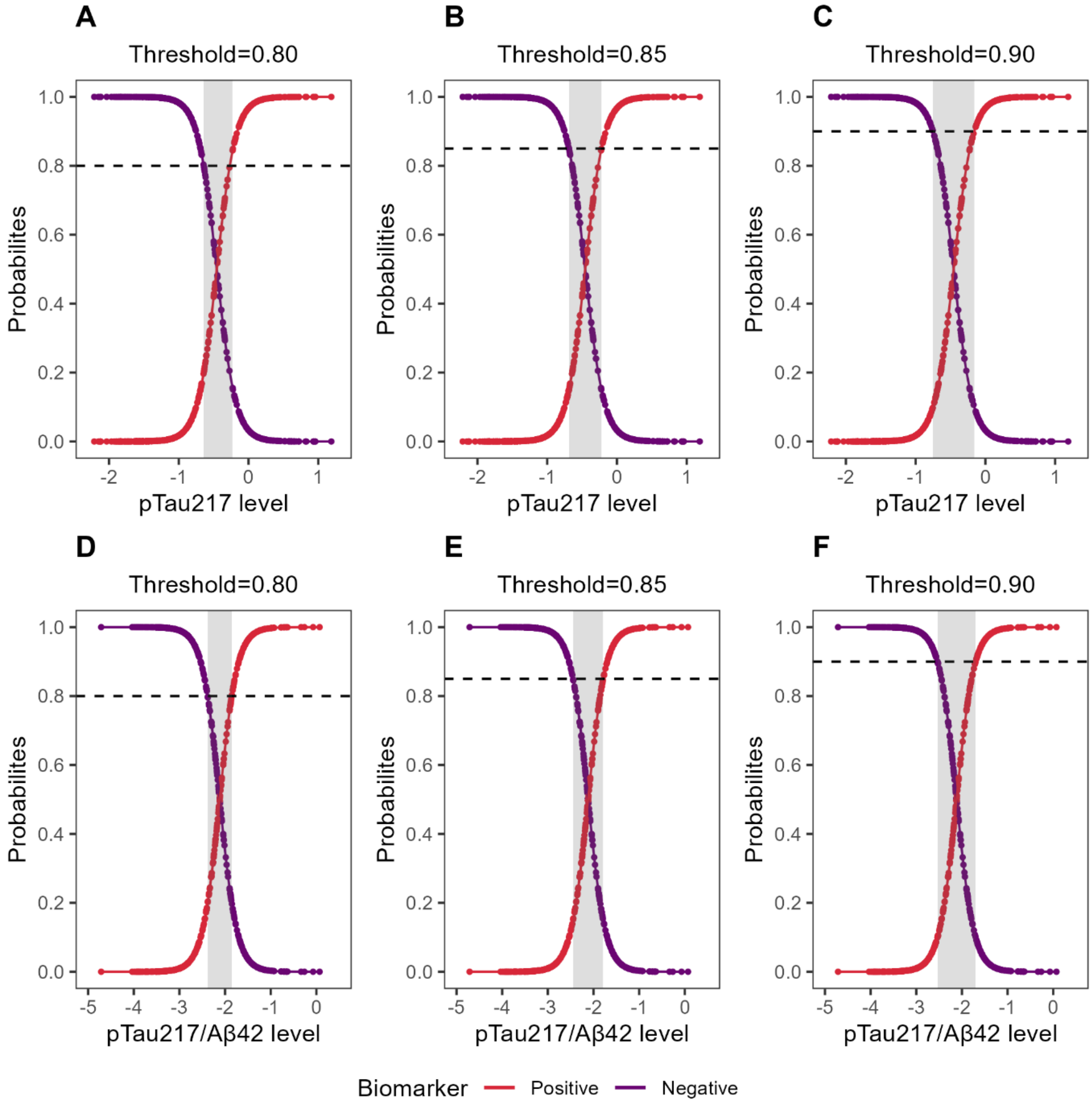

Supplementary Figure 16. Class membership probabilities and indeterminate ranges for plasma pTau217 and pTau217/Aβ42 in the pooled Cog-Aging-Study–Brazil/GeNED.ar–Argentina dataset. The purple line shows the probability of being classified as pTau217+ (A-C) or pTau217/Aβ42+ (D-F), and the red line shows the probability of being classified as pTau217- or pTau217/Aβ42- across biomarker levels. The grey shading indicates the range where classification was uncertain, defined by probability thresholds of <0.80 (A, D), <0.85 (B, E), or <0.90 (C, F). Plasma pTau217 and pTau217/Aβ42+ levels were log-transformed.

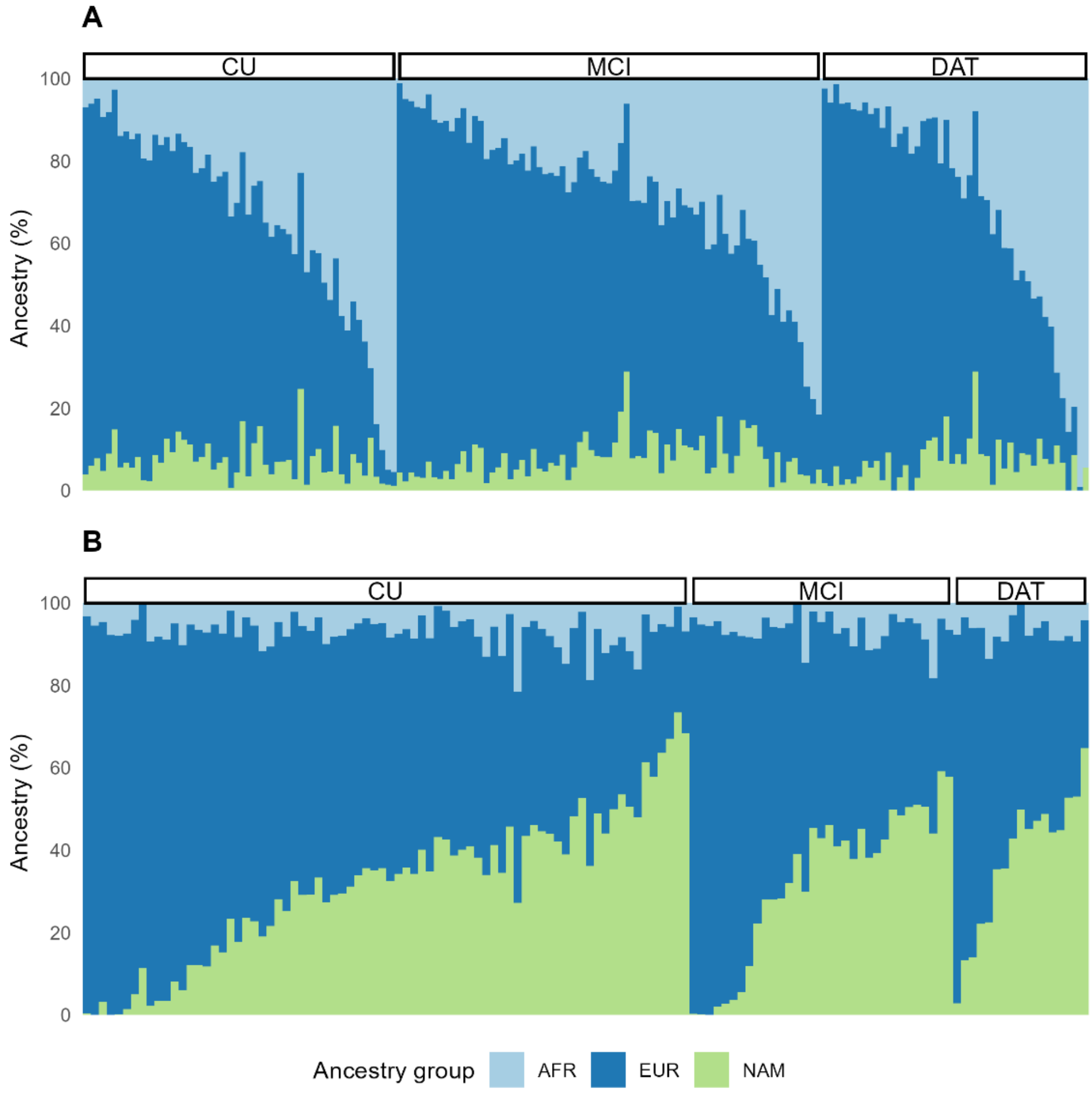

Supplementary Figure 17. Genetic ancestry of participants from Cog-Aging-Study–Brazil and GeNED.ar–Argentina. Each vertical bar represents one participant and shows individual admixture proportions for African (light blue), European (dark blue), and Native American (green) ancestry. Cog-Aging-Study–Brazil, n=173; GeNED.ar–Argentina, n=126. CU, cognitively unimpaired; MCI, mild cognitive impairment; DAT, dementia of the Alzheimer's type; AFR, African; EUR, European; NAM, Native American.

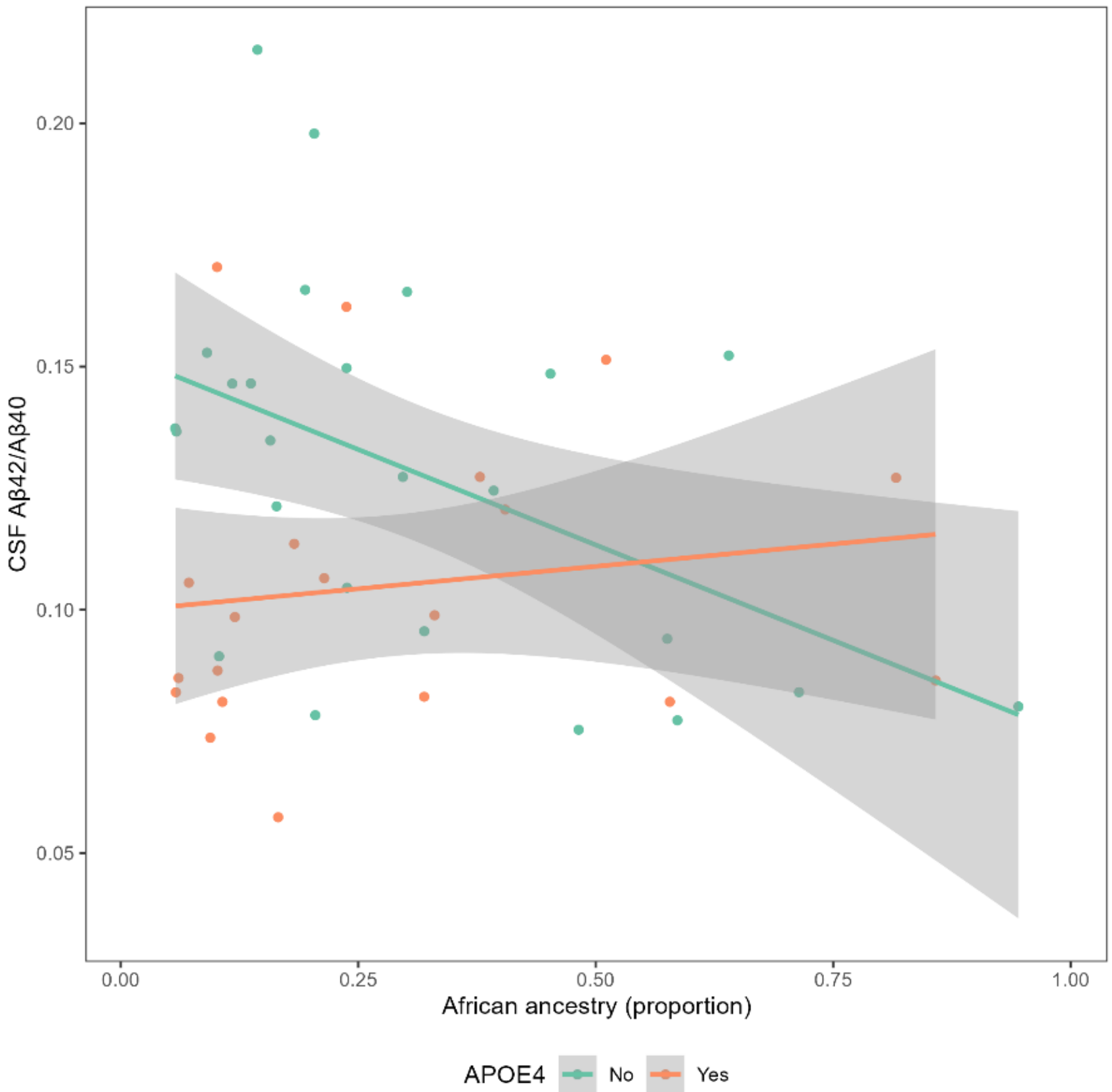

Supplementary Figure 18. Effect of African ancestry on the association between *APOE-ε4* and amyloid pathology. Dots show the association between global African ancestry and CSF Aβ42/Aβ40 levels (indicator of amyloid pathology) in *APOE-ε4* carriers (orange) and non-carriers (green). Trend lines were overlaid for visualisation purposes only.

Supplementary Table 1. Model fit indices in Gaussian Mixture Modelling of CSF biomarkers in Cog-Aging-Study–Brazil.

CSF biomarker	Number of components	Variance structure	Log likelihood	BIC	Means	Variances	Proportions
Aβ42/Aβ40	1	Equal	206.67	403.86	403.86	1.56×10^{-3}	1
	1	Varying	206.67	403.86	403.86	1.56×10^{-3}	1
	**2**	**Equal**	**218.18**	**417.41**	**405.10**	$\mathbf{4.07\times10^{-4}}$	**0.55; 0.45**
	2	Varying	220.03	416.37	401.99	2.47×10^{-4}; 6.41×10^{-4}	0.48; 0.52
	3	Equal	220.23	412.04	398.22	3.04×10^{-4}	0.53; 0.42; 0.05
	3	Varying	223.13	408.37	393.23	1.14×10^{-4}; 2.61×10^{-5}; 6.26×10^{-4}	0.35; 0.13; 0.53
pTau181	1	Equal	-91.70	-192.88	-192.88	4.23	0.29
	1	Varying	-91.70	-192.88	-192.88	4.23	0.29
	**2**	**Equal**	**-84.94**	**-188.83**	**-203.38**	**3.80; 4.70**	**0.09**
	2	Varying	-84.94	-193.56	-208.08	3.80; 4.70	0.09; 0.09
	3	Equal	-84.95	-198.33	-259.67	3.73; 3.98; 4.72	0.08
	3	Varying	-82.65	-203.19	-228.39	3.65; 4.05; 4.69	0.06; 0.01; 0.09

The model that fulfilled the criteria of best fit for each biomarker is highlighted in bold. Note that models with higher BIC are preferred using the formula implemented in *mclust* R package (see Methods). pTau181 levels were log-transformed. Aβ42/Aβ40-models, n=114; pTau181-models, n=114. BIC, Bayesian information criterion.

Supplementary Table 2. Model fit indices in Gaussian Mixture Modelling of plasma biomarkers in Cog-Aging-Study–Brazil.

Plasma biomarker	Number of components	Variance structure	Log likelihood	BIC	Means	Variances	Proportions
pTau217	1	Equal	-337.43	-686.30	-0.72	0.54	1
	1	Varying	-337.43	-686.30	-0.72	0.54	1
	**2**	**Equal**	**-314.66**	**-652.19**	**-1.18; 0.09**	**0.17**	**0.64; 0.36**
	2	Varying	-314.69	-657.97	-1.19; 0.07	0.16; 0.18	0.63; 0.37
	3	Equal	-313.93	-662.16	-1.35; -0.77; 0.19	0.12	0.42; 0.28; 0.31
	3	Varying	-313.48	-672.70	-1.44; -0.87; 0.12	0.09; 0.09; 0.15	0.34; 0.31; 0.35
pTau217/Aβ42	1	Equal	-369.17	-749.74	-2.51	0.7	1
	1	Varying	-369.17	-749.74	-2.51	0.7	1
	**2**	**Equal**	**-359.69**	**-742.17**	**-2.96; -1.62**	**0.3**	**0.67; 0.33**
	2	Varying	-356.30	-741.09	-3.19; -2.16	0.13; 0.63	0.34; 0.66
	3	Equal	-359.73	-753.64	-3.00; -2.89; -1.61	0.30	0.38; 0.3; 0.33
	3	Varying	-356.34	-758.26	-3.19; -2.63; -1.87	0.13; 0.52; 0.50	0.33; 0.27; 0.40

The model that fulfilled the criteria of best fit for each biomarker is highlighted in bold. Note that models with higher BIC are preferred using the formula implemented in *mclust* R package (see Methods). If the BIC difference between two models was less than 5, the most parsimonious model was chosen. Biomarker levels were log-transformed. pTau217-models, n=304; pTau217/Aβ42-models, n=298. BIC, Bayesian information criterion.

Supplementary Table 3. Model fit indices in Gaussian Mixture Modelling of plasma biomarkers in GeNED.ar–Argentina.

Plasma biomarker	Number of components	Variance structure	Log likelihood	BIC	Means	Variances	Proportions
pTau217	1	Equal	-126.87	-263.53	-0.93	0.39	1
	1	Varying	-126.87	-263.53	-0.93	0.39	1
	**2**	**Equal**	**-118.75**	**-257.08**	**-1.20; -0.05**	**0.16**	**0.77; 0.23**
	2	Varying	-118.69	-261.86	-1.22; -0.16	0.14; 0.21	0.73; 0.27
	3	Equal	-118.79	-266.96	-1.31; -1.11; -0.04	0.15	0.30; 0.48; 0.22
	3	Varying	-118.00	-275.19	-1.47; -1.00; -0.23	0.09; 0.04; 0.23	0.38; 0.30; 0.32
pTau217/Aβ42	1	Equal	-145.22	-300.18	-2.46	0.54	1
	1	Varying	-145.22	-300.18	-2.46	0.54	1
	**2**	**Equal**	**-130.85**	**-281.19**	**-2.88; -1.55**	**0.16**	**0.69; 0.31**
	2	Varying	-129.67	-283.72	-2.96; -1.72	0.11; 0.27	0.60; 0.40
	3	Equal	-130.86	-290.96	-2.90; -2.85; -1.54	0.16	0.37; 0.32; 0.31
	3	Varying	-129.50	-297.99	-3.08; -2.85; -1.77	0.10; 0.06; 0.29	0.31; 0.26; 0.43

The model that fulfilled the criteria of best fit for each biomarker is highlighted in bold. Note that models with higher BIC are preferred using the formula implemented in *mclust* R package (see Methods). Biomarker levels were log-transformed. pTau217-models, n=134; pTau217/Aβ42-models, n=131. BIC, Bayesian information criterion.

Supplementary Table 4. Model fit indices in Gaussian Mixture Modelling of plasma biomarkers in the pooled Cog-Aging-Study–Brazil/GeNED.ar–Argentina dataset.

Plasma biomarker	Number of components	Variance structure	Log likelihood	BIC	Means	Variances	Proportions
pTau217	1	Equal	-471.00	-954.16	-0.79	0.50	1
	1	Varying	-471.00	-954.16	-0.79	0.50	1
	**2**	**Equal**	**-438.88**	**-902.09**	**-1.18; 0.06**	**0.17**	**0.68; 0.32**
	2	Varying	-438.88	-908.16	-1.20; 0.02	0.16; 0.19	0.66; 0.34
	3	Equal	-438.96	-914.42	-1.34; -0.92; 0.12	0.14	0.39; 0.32; 0.29
	3	Varying	-438.08	-924.82	-1.46; -0.94; 0.02	0.09; 0.07; 0.19	0.34; 0.31; 0.35
pTau217/Aβ42	1	Equal	-518.10	-1048.34	-2.50	0.65	1
	1	Varying	-518.10	-1048.34	-2.50	0.65	1
	**2**	**Equal**	**-499.36**	**-1022.99**	**-2.93; -1.59**	**0.26**	**0.68; 0.32**
	2	Varying	-497.19	-1024.70	-3.08; -1.94	0.17; 0.48	0.49; 0.51
	3	Equal	-499.42	-1035.23	-2.98; -2.87; -1.58	0.26	0.34; 0.34; 0.32
	3	Varying	-495.61	-1039.76	-3.14; -3.01; -1.91	0.23; 0.07; 0.44	0.28; 0.22; 0.50

The model that fulfilled the criteria of best fit for each biomarker is highlighted in bold. Note that models with higher BIC are preferred using the formula implemented in *mclust* R package (see Methods). Biomarker levels were log-transformed. pTau217-models, n=134; pTau217/Aβ42-models, n=131. BIC, Bayesian information criterion.

Supplementary Table 5. Predictive performance of plasma pTau217 and pTau217/Aβ42 cut-offs from the pooled Cog-Aging-Study–Brazil/GeNED.ar–Argentina dataset.

Biomarker	Threshold	Single cut-off	Lower cut-off	Upper cut-off	Indeterm (%)	PPV (95% CI)	NPV (95% CI)	Sensitivity (95% CI)	Specificity (95% CI)
pTau217	-	≥ 0.63	-	-	-	0.85 (0.68-0.94)	0.72 (0.49-0.88)	0.82 (0.64-0.92)	0.76 (0.53-0.90)
	0.80	-	≤ 0.53	≥ 0.79	8.9	0.88 (0.69-0.96)	0.76 (0.53-0.90)	0.84 (0.65-0.94)	0.81 (0.57-0.93)
	0.85	-	≤ 0.50	≥ 0.80	11.1	0.88 (0.69-0.96)	0.81 (0.57-0.93)	0.88 (0.69-0.96)	0.81 (0.57-0.93)
	0.90	-	≤ 0.47	≥ 0.85	17.8	0.86 (0.67-0.95)	0.80 (0.55-0.93)	0.86 (0.67-0.95)	0.80 (0.55-0.93)
pTau217/Aβ42	-	≥ 0.12	-	-	-	0.88 (0.69-0.96)	0.68 (0.46-0.85)	0.78 (0.59-0.89)	0.81 (0.57-0.93)
	0.80	-	≤ 0.09	≥ 0.16	14.0	0.90 (0.71-0.97)	0.69 (0.44-0.86)	0.79 (0.60-0.91)	0.85 (0.58-0.96)
	0.85	-	≤ 0.09	≥ 0.17	18.6	0.89 (0.69-0.97)	0.69 (0.44-0.86)	0.77 (0.57-0.90)	0.85 (0.58-0.96)
	0.90	-	≤ 0.08	≥ 0.18	20.9	0.89 (0.69-0.97)	0.73 (0.48-0.89)	0.81 (0.60-0.92)	0.85 (0.58-0.96)

Indeterm, indeterminate; PPV, positive predictive value; NPV, negative predictive value, CI, confidence interval. Cut-offs for pTau217 are expressed in pg/mL, and were evaluated in the subset of participants from Cog-Aging-Study–Brazil with paired CSF-plasma data (n=45).

Supplementary Table 6. Genetic ancestry in Cog-Aging-Study–Brazil and GeNED.ar–Argentina.

Ancestry group (proportion)	Cog-Aging-Study–Brazil (n=173)	GeNED.ar–Argentina (n=126)
African (mean, SD)	0.30 (0.22)	0.07 (0.04)
European (mean, SD)	0.62 (0.22)	0.61 (0.18)
Native American (mean, SD)	0.07 (0.05)	0.33 (0.18)

Values represent the mean (SD) proportion of genetic ancestry components estimated for each cohort. SD, standard deviation.

References

1. de Paula JJ, Bertola L, Ávila RT, et al. Clinical Applicability and Cutoff Values for an Unstructured Neuropsychological Assessment Protocol for Older Adults with Low Formal Education. Herholz K, ed. *PLoS One*. 2013;8(9):e73167. doi:10.1371/journal.pone.0073167

2. American Psychiatric Association. *Diagnostic and Statistical Manual of Mental Disorders*. American Psychiatric Association Publishing; 2022. doi:10.1176/appi.books.9780890425787

3. McKhann GM, Knopman DS, Chertkow H, et al. The diagnosis of dementia due to Alzheimer's disease: Recommendations from the National Institute on Aging-Alzheimer's Association workgroups on diagnostic guidelines for Alzheimer's disease. *Alzheimer's Dement*. 2011;7(3):263-269. doi:10.1016/j.jalz.2011.03.005

4. Marees AT, de Kluiver H, Stringer S, et al. A tutorial on conducting genome-wide association studies: Quality control and statistical analysis. *Int J Methods Psychiatr Res*. 2018;27(2). doi:10.1002/mpr.1608

5. Dalmasso MC, Brusco LI, Olivar N, et al. Transethnic meta-analysis of rare coding variants in PLCG2, ABI3, and TREM2 supports their general contribution to Alzheimer's disease. *Transl Psychiatry*. 2019;9(1):55. doi:10.1038/s41398-019-0394-9

6. Dalmasso MC, de Rojas I, Olivar N, et al. The first genome-wide association study in the Argentinian and Chilean populations identifies shared genetics with Europeans in Alzheimer's disease. *Alzheimer's Dement*. 2024;20(2):1298-1308. doi:10.1002/alz.13522

7. Taliun D, Harris DN, Kessler MD, et al. Sequencing of 53,831 diverse genomes from the NHLBI TOPMed Program. *Nature*. 2021;590(7845):290-299. doi:10.1038/s41586-021-03205-y

8. Das S, Forer L, Schönherr S, et al. Next-generation genotype imputation service and methods. *Nat Genet*. 2016;48(10):1284-1287. doi:10.1038/ng.3656

9. Fuchsberger C, Abecasis GR, Hinds DA. minimac2: faster genotype imputation. *Bioinformatics*. 2015;31(5):782-784. doi:10.1093/bioinformatics/btu704

10. Alexander DH, Novembre J, Lange K. Fast model-based estimation of ancestry in unrelated individuals. *Genome Res*. 2009;19(9):1655-1664. doi:10.1101/gr.094052.109
