# Supplementary material for: Diagnostic performance of plasma pTau217 in genetically admixed South American populations
Source: Nat Commun. 2026 Aug 6;17:9464. doi: 10.1038/s41467-026-76434-2 (PMC13542151; doi:10.1038/s41467-026-76434-2)
Supplement: Supplementary file 2 [file 41467_2026_76434_MOESM2_ESM.pdf]

nature portfolio

Corresponding author(s): Alfredo Ramirez

Last updated by author(s): Jul 7, 2026

Reporting Summary

Nature Portfolio wishes to improve the reproducibility of the work that we publish. This form provides structure for consistency and transparency in reporting. For further information on Nature Portfolio policies, see our Editorial Policies and the Editorial Policy Checklist.

Statistics

For all statistical analyses, confirm that the following items are present in the figure legend, table legend, main text, or Methods section.

n/a	Confirmed	
☐	☒	The exact sample size (*n*) for each experimental group/condition, given as a discrete number and unit of measurement
☐	☒	A statement on whether measurements were taken from distinct samples or whether the same sample was measured repeatedly
☐	☒	The statistical test(s) used AND whether they are one- or two-sided<br>*Only common tests should be described solely by name; describe more complex techniques in the Methods section.*
☐	☒	A description of all covariates tested
☐	☒	A description of any assumptions or corrections, such as tests of normality and adjustment for multiple comparisons
☐	☒	A full description of the statistical parameters including central tendency (e.g. means) or other basic estimates (e.g. regression coefficient) AND variation (e.g. standard deviation) or associated estimates of uncertainty (e.g. confidence intervals)
☐	☒	For null hypothesis testing, the test statistic (e.g. *F*, *t*, *r*) with confidence intervals, effect sizes, degrees of freedom and *P* value noted<br>*Give P values as exact values whenever suitable.*
☒	☐	For Bayesian analysis, information on the choice of priors and Markov chain Monte Carlo settings
☒	☐	For hierarchical and complex designs, identification of the appropriate level for tests and full reporting of outcomes
☐	☒	Estimates of effect sizes (e.g. Cohen's *d*, Pearson's *r*), indicating how they were calculated

Our web collection on statistics for biologists contains articles on many of the points above.

Software and code

Policy information about availability of computer code

Data collection	No software was used
Data analysis	All statistical analyses were performed using R language (version 4.6.0). Packages used include: mclust (version 6.1.2), bootLR (version 1.0.2), pROC (version 1.19.0.1), and ggplot2 (4.0.3). GWAS analysis was performed using PLINK (v1.9) and ADMIXTURE (v1.3.010)

For manuscripts utilizing custom algorithms or software that are central to the research but not yet described in published literature, software must be made available to editors and reviewers. We strongly encourage code deposition in a community repository (e.g. GitHub). See the Nature Portfolio guidelines for submitting code & software for further information.

Data

Policy information about availability of data

All manuscripts must include a data availability statement. This statement should provide the following information, where applicable:

- Accession codes, unique identifiers, or web links for publicly available datasets
- A description of any restrictions on data availability
- For clinical datasets or third party data, please ensure that the statement adheres to our policy

The data generated in this study are available under restricted access due to ethical and legal restrictions related to the protection of participants' clinical, cognitive, and genetic information, in accordance with the terms of the informed consent and the approval granted by the local Research Ethics Committees. Access to these data requires prior authorisation from the local Research Ethics Committees.
For data relating to the Cog-Aging-Study, access requests should be sent by email to Professor Maria Aparecida Camargos-Bicalho (macbicalho@ufmg.br), who is

responsible for the study. Requests will be reviewed and a response provided within 30 days. Access, when granted, will be provided for a period of 12 months following approval, restricted to the specific purpose stated in the request and to researchers who agree to comply with the terms established by the local Ethics Committee. The processed data required to interpret, verify, and extend the findings reported in this article can be made available for academic, non-commercial research purposes only.

Access to the GeNED.ar data is available upon request. Data access requests should be submitted by email to Dr. Maria Carolina Dalmasso (mcdalmasso@unaj.edu.ar) at the Studies in Neuroscience and Complex Systems unite (ENyS-CONICET-HEC-UNAJ). Requests will be reviewed, and a response will be provided within 30 days. If approved, access will be granted for a period of six months and will be limited to the specific research purpose described in the request. Access will be restricted to researchers who agree to comply with the conditions established by the local Research Ethics Committee. The processed data necessary to interpret, verify, and extend the findings reported in this article will be made available exclusively for academic, non-commercial research purposes.

Research involving human participants, their data, or biological material

Policy information about studies with human participants or human data. See also policy information about sex, gender (identity/presentation), and sexual orientation and race, ethnicity and racism.

Reporting on sex and gender	The association between biomarkers and each health or clinical-related variable was examined using separate regression models: linear regression models (continuous variables) or ANCOVA (dichotomous variables) with biomarker levels as outcomes. All models were run both unadjusted and adjusted for age and sex (biological attribute, self-reported).
Reporting on race, ethnicity, or other socially relevant groupings	Global genetic ancestry was estimated using GWAS data. Briefly, 356 autosomal Ancestry Informative Markers (AIMs), evenly distributed among chromosomes, were extracted from Cog-Aging-Study–Brazil and GeNED.ar–Argentina, as well as from reference populations in the 1000 Genomes Project (http://www.internationalgenome.org/): Europeans (CEU, n=85), Africans (YRI, n=88), and Native Americans (NAM, n=43). All datasets were merged in one PLINK v1.9 file (www.cog-genomics.org/plink/1.9/), and global ancestry was estimated using ADMIXTURE v1.3.010. The impact of global African ancestry on the association between APOE-ε4 allele and biomarker levels was evaluated with the interaction term APOE-ε4×proportion of global African ancestry as predictor.
Population characteristics	In Cog-Aging-Study–Brazil, a total of 353 participants were included. Of these, 114 had CSF samples and 304 plasma samples. However, 49 had only CSF samples available, 239 had only plasma samples, 45 had paired CSF and plasma samples collected within 12 months, and 20 had unpaired CSF and plasma samples collected more than 12 months apart. Differences between groups partly reflect distinct recruitment pathways, as described in the Supplementary Methods. The average age across groups ranged from 72.8 years in the CSF-only group to 77 years in the plasma-only group. Women made up between 51.1% and 71.1% of each group. APOE-ε4 carriers were more common among participants with paired samples (44.4%) and unpaired samples (45.0%) than among those with plasma only (28.8%). Years of education were generally low, with means from 4.4 to 6.5. Clinical diagnoses varied across groups. Among those with CSF only, 43.5% were cognitively unimpaired (CU), 21.7% had mild cognitive impairment (MCI), and 34.8% had dementia of the Alzheimer's type (DAT). In the plasma-only group, 34.7% were CU, 45.6% MCI, and 19.7% DAT. For participants with paired CSF and plasma, at the time of CSF collection, 13.3% were CU, 26.7% MCI, and 60% DAT, whereas at plasma collection, 15.6% were CU, 35.6% MCI, and 48.9% DAT. Among those with unpaired samples, 10% were classified as CU, 70% as MCI, and 20% as DAT at both collection times. Across groups, BMI and kidney function were similar. A high prevalence of vascular and metabolic comorbidities was observed, including hypertension (62.2%-95.0%), dyslipidaemia (50.0%-80.0%), and diabetes (27.8%-35.6%). The history of smoking ranged from 27.3% to 41.2%, and depression was reported by 44.2% to 60.3% of participants.<br>In GeNED.ar–Argentina, 134 participants with plasma samples were included in the study. The mean age was 71.7 years, and 70.1% were women. APOE-ε4 carriers were less frequent (27.9%) than in Cog-Aging-Study–Brazil. Participants had, on average, higher educational attainment (8.7 years). Most individuals were CU (60.5%), followed by those with MCI (26.1%) and DAT (13.4%). The mean BMI was 28.9, and the prevalence of diabetes was 35.1%, similar to that in Cog-Aging-Study–Brazil. Hypertension was reported by 68.1% of participants, dyslipidaemia by 37.5%, smoking by 16.3%, and depression by 16.3%, all less frequent than in Cog-Aging-Study–Brazil. Information on alcoholism and kidney function was not available.
Recruitment	The Cog-Aging-Study is a longitudinal cohort established at the Health Reference Centre for Older Adults, University Hospital, Federal University of Minas Gerais (Belo Horizonte, Brazil). A total of 353 individuals aged ≥60 years were enrolled through community-based senior socialization groups and referrals from the Geriatric Service of the University Hospital, and were followed annually. All participants underwent standardised clinical, cognitive, and functional evaluations conducted by trained geriatric specialists. CSF collection was performed both in participants recruited through the cognitive outpatient clinic and in individuals undergoing procedures requiring spinal anaesthesia, whereas plasma samples were obtained more broadly within the cohort. Consequently, availability of CSF and paired samples was influenced by recruitment pathway, clinical indication, and logistical/ethical constraints.<br>The GeNED.ar study (Genetics and Neuroimaging of Aging and Dementia in Argentina) is an ongoing study based at Hospital El Cruce/ENyS-CONICET, National University Arturo Jauretche (Florencio Varela, Buenos Aires, Argentina). Individuals aged ≥60 years were recruited from the community and a memory clinic. All participants underwent standardised clinical, cognitive, and functional assessments, as well as brain MRI, with final diagnoses assigned by multidisciplinary consensus. A total of 134 participants were included.
Ethics oversight	The study was approved by the Ethics committees from the University Hospital of the Universidade Federal de Minas Gerais (CAAE 68351023.6.0000.5149, Cog-Aging-Study) and from Hospital El Cruce (D00079, GeNED.ar study).

Note that full information on the approval of the study protocol must also be provided in the manuscript.

Field-specific reporting

Life sciences study design

All studies must disclose on these points even when the disclosure is negative.

Sample size	No statistical method was used to predetermine sample size. Outliers were excluded according to the prespecified criteria described below; otherwise, all available samples meeting the inclusion criteria were included in the analyses.
Data exclusions	In the plasma biomarker analyses, observations were excluded if biomarker values were more than three standard deviations above or below the mean. In the genetic analysis, samples with a call rate below 98%, excess of heterozygosity, sex discrepancies, duplicates, or evidence of relatedness (PI-HAT>0.2) were excluded. Variants with a call rate below 97%, a deviation from the Hardy-Weinberg equilibrium (HWE, p<1×10-6), or differential missingness between cases and controls were also removed.
Replication	To verify reproducibility, the same plasma analyses were performed in both cohorts.
Randomization	This study uses existing observational cohorts. Participants were not assigned to interventions, so randomization is not applicable.
Blinding	Participants were not assigned to interventions and data analysis was performed on de-identified datasets, so blinding is not applicable.

Reporting for specific materials, systems and methods

We require information from authors about some types of materials, experimental systems and methods used in many studies. Here, indicate whether each material, system or method listed is relevant to your study. If you are not sure if a list item applies to your research, read the appropriate section before selecting a response.

Materials & experimental systems

n/a	Involved in the study	
☒	☐	Antibodies
☒	☐	Eukaryotic cell lines
☒	☐	Palaeontology and archaeology
☒	☐	Animals and other organisms
☐	☒	Clinical data
☒	☐	Dual use research of concern
☒	☐	Plants

Methods

n/a	Involved in the study	
☒	☐	ChIP-seq
☒	☐	Flow cytometry
☒	☐	MRI-based neuroimaging

Clinical data

Policy information about clinical studies

All manuscripts should comply with the ICMJE guidelines for publication of clinical research and a completed CONSORT checklist must be included with all submissions.

Clinical trial registration	Not applicable
Study protocol	Not applicable
Data collection	The Cog-Aging-Study is a longitudinal cohort established at the Health Reference Centre for Older Adults, University Hospital, Federal University of Minas Gerais (Belo Horizonte, Brazil). A total of 353 individuals aged ≥60 years were enrolled through community-based senior socialization groups and referrals from the Geriatric Service of the University Hospital, and were followed annually. All participants underwent standardised clinical, cognitive, and functional evaluations conducted by trained geriatric specialists.<br>The GeNED.ar study (Genetics and Neuroimaging of Aging and Dementia in Argentina) is an ongoing study based at Hospital El Cruce/ ENyS-CONICET, National University Arturo Jauretche (Florencio Varela, Buenos Aires, Argentina). Individuals aged ≥60 years were recruited from the community and a memory clinic. All participants underwent standardised clinical, cognitive, and functional assessments, as well as brain MRI, with final diagnoses assigned by multidisciplinary consensus. A total of 134 participants were included.
Outcomes	Not applicable

Plants

Seed stocks	Not applicable
Novel plant genotypes	Not applicable
Authentication	Not applicable
