# Supplementary material for: Diagnostic performance of plasma pTau217 in genetically admixed South American populations
Source: Nat Commun. 2026 Aug 6;17:9464. doi: 10.1038/s41467-026-76434-2 (PMC13542151; doi:10.1038/s41467-026-76434-2)
Supplement: Supplementary file 3 [file 41467_2026_76434_MOESM3_ESM.pdf]

nature portfolio

Peer Review File

Diagnostic performance of plasma pTau217 in genetically admixed South American populations

Corresponding Author: Professor Alfredo Ramirez

This file contains all reviewer reports in order by version, followed by all author rebuttals in order by version.

Version 0:

Reviewer comments:

Reviewer #1

(Remarks to the Author)
Methods
1. Page 13, line 345. Presenting associations between plasma biomarkers and formal cognitive and functional scales would help fill an important data gap in underrepresented groups. At least include comparisons of plasma biomarker levels by CDR global score and correlations between biomarker levels and CDR sum of boxes, FAQ, and CERAD scores.
2. Page 14, line 360. In the description of blood sample processing also mention room temperature during centrifugation, estimated average needle-to-freezer time, and brand and type (or catalog number) of "low protein binding tubes" used. This is especially critical for local researchers or other researchers in Latin America looking to replicate the findings.
3. Page 14, line 373. Describe the performance features of the biomarkers in your lab (at least the overall coefficient of variation for each marker), whether samples were run as duplicates, and whether Simoa operators were blinded to clinical data.

Results
1. Page 5, line 96. "The same procedure was applied" is not possible, since GENED.ar did not have CSF data. The phrase needs to be reworded. Also the rationale and pros and cons of generating cutoffs based on CSF biomarkers or clinical diagnosis should be more clearly stated.
2. Page 5, line 103. Provide the overall prevalence of APOE e3/e3, APOE e3/e4 and APOE e4/e4 genotypes in each cohort.
3. Page 6, line 22. Given the novelty of the biofluid phenotypes in these Latin American populations, there is a unique opportunity to provide elements to close the age, race and sex data gaps relevant to underrepresented groups. This is important for comparison with other populations and replication purposes. These sections should start with a more elementary description of data in both cohorts: 1) Results of test of normality for the biofluid data, 2) Differences in fluid biomarker by sex, 3) Overall and within-diagnostic group relationships between biomarkers and age. 4) Median (±IQR) CSF and biomarker levels by clinical diagnostic groups.
4. Page 6, line 22. This is an opportunity to present data in the context of the ATN(I) system. It's understandable to narrow the scope of the study to Abeta and p-tau markers, but since the 4 Neuro-Plex E kit was used for fluid phenotyping, the data on NfL (N) and GFAP (I) should be presented, at least median and IQR by diagnostic group. This is in the spirit of taking advantage of the uniqueness of these cohorts and to allow a richer biofluid characterization of underrepresented groups. For example, because there is no neuropath data, seen rising levels of GFAP with disease severity would support that the cohorts are enriched for typical AD pathology.
5. Page 6, line 22. If CSF values in this study are given the important role of being used as determinants for amyloid pathology, their performance should be further described as well. At least provide 1) levels of CSF biomarker by clinical diagnostic group, 2) CSF cutoff values used for amyloid positivity, and 3) correlations between CSF and plasma biomarkers, when available.

Discussion
1. Page 10, line 241. This section should discuss the clinical associations of the plasma biomarkers and their diagnostic performance in contrast to those observed in the White, and non-diverse populations: e.g., Native-American, African, Asian. My gestalt is that the accuracy may be similar to that described in the White population, with amyloid PET or neuropath-defined cohorts. This scope is relevant for two reasons: 1) highlight similarities and discrepancies to guide further clinical tool development and investigations on variables that mediate the diagnostic utility of these markers in different populations, and 2) advance practical parameters to implement precision medicine when clinicians encounter diverse populations (e.g.,

Can results of an AD plasma biomarker in Brazilian or Argentinian people living in the US or Europe be equally regarded and interpreted?).
2. Page 11, line 298. The study of the effect of African ancestry is important. A unique opportunity in this cohort, however, is the study of the particular effects of local genetic backgrounds (e.g., pardo, mestizo, indigenous) on the clinical associations of the biomarkers. If not pursued, this should be at least acknowledged as an area of opportunity.
3. Page 12, line 321. Other limitations to acknowledge: 1. CSF is not a perfect gold-standard. Studies on path-confirmed cases are still needed in diverse populations. 2) Both cohorts recruit through single centers and the racial and ethnic heterogeneity within a given Latin American country can still be missed. 3) Simoa is not an optimal analytical platform for AD fluid biomarkers. In head-to-head studies that compared either p-tau217 or Ab42/Ab40 measured with different analytical platforms, IPMS was significantly superior than Simoa. ECL also has better performance than Simoa for p-tau217. The performance for Ab with Simoa is among the worst to identify amyloid pathology. The use of Simoa to conduct the analyses should also be mentioned in the abstract.

Reviewer #2

(Remarks to the Author)
This study evaluates plasma pTau217 (and pTau217/Aβ42) as biomarkers for Alzheimer's disease in two cohorts from Brazil and Argentina, addressing the lack of data from low and middle income countries. Using data-driven cut-offs (GMM), the authors show good concordance of these plasma biomarkers with clinical AD diagnosis and, in a subgroup of the Brazilian cohort, with CSF status. Demographic and clinical factors (APOE ε4, BMI, eGFR, education) influenced biomarker levels. African ancestry modified APOE effects on CSF.
The main strength and novelty of this study is including genetically admixed South American populations, and two independent cohorts. I particularly like the findings on the effect of ancestry in blood biomarkers.

There are some limitations that need to be addressed:

1. A main limitation of the study is that only a subgroup of the Brazilian cohort had CSF data available, and some of these were not temporally matched with plasma collection, limiting validation against a biological reference standard. Consequently, concordance is largely based on clinical diagnosis. In addition, the absence of CSF or PET data in the Argentinian cohort further restricts conclusions about diagnostic accuracy in that population.

2. There are multiple differences between participants with plasma only, CSf only, and those with both plasma and CSF in the Brazilian cohort, raising concerns about potential selection bias. The criteria for CSF availability and the representativeness of this subgroup should be clarified.

3. The rationale for using Youden's index derived from logistic regression models including only CU and AD dementia participants is unclear.

4. It is also unclear why plasma biomarker cut-offs were primarily derived using a data-driven Gaussian mixture modelling approach rather than being directly calculated against the available CSF reference standard.

5. The finding that the pTau217/Aβ42 ratio shows a slightly larger grey zone than pTau217 alone contrasts with previous studies and warrants further discussion. May this may be partly related to the fact that the ratio combines measurements obtained from different analytical assays (Alzpath for pTau217 and Quanterix N4PE assay for Aβ42)? which should be explicitly acknowledged as a limitation.

6. Finally, when evaluating the impact of comorbidities such as hypertension, dyslipidemia, and diabetes on plasma biomarkers, it is unclear how the authors disentangle effects of the conditions themselves from those of their treatments. This limitation should be discussed, and medication use considered where possible.

Reviewer #3

(Remarks to the Author)
This is an important study seeking to evaluate plasma ptau217 and its ratio with AB42 in two Latin American cohorts. These works are urgently needed in helping to democratize blood biomarker use worldwide. The work needs quite a few necessary improvements to get to the level needed for this journal. I have a few comments.

- It is unclear to what end the cutpoint estimation serves. Instead, I suggest using published cutpoints for the assays used and examine their transferability.

- - Comment on why or why not cutpoints from elsewhere may or may not be transferable to yours. Include analytical and clinical reasoning.

- The authors should use the plasma-CSF paired samples to a better advantage than they do currently. The main focus of the work should be on plasma. However, in subanalyses, show how well correlated the plasma and CSF set are.

- In Table 1, focus on only plasma and have a separate table for characteristics of CSF-plasma pairs.

- Use pooled samples from both cohorts to increase sample size e.g. in cutpoint estimation analyses.

- If you have clinical follow-up information, use that to evalaute the biomarker and cutpoint performance.

Version 1:

Reviewer comments:

Reviewer #1

(Remarks to the Author)
The authors have thoroughly addressed the comments provided by the other two reviewers and me. This has translated in incorporation of additional data and analyses that, in my opinion, solidify the findings, increase their internal validity and increase their generalizability. In its current form, the manuscript will constitute a precedent of a sound methodology for studying AD fluid biomarkers in new populations. The findings support the value of plasma AD biomarkers for identification of people with Alzheimer's disease in Argentina and Brazil. This is outstanding work, which is expected to have high impact. I recommend to accept.

Reviewer #2

(Remarks to the Author)
The authors have partially addressed the comments raised during the review process. Those issues that could not be fully addressed are appropriately and transparently acknowledged as limitations of the study. Overall, I believe the manuscript has been improved and is suitable for publication.

Reviewer #3

(Remarks to the Author)
The authors have addressed my concerns beautifully. I recommend acceptance.

Open Access This Peer Review File is licensed under a Creative Commons Attribution 4.0 International License, which permits use, sharing, adaptation, distribution and reproduction in any medium or format, as long as you give appropriate credit to the original author(s) and the source, provide a link to the Creative Commons license, and indicate if changes were made.
In cases where reviewers are anonymous, credit should be given to 'Anonymous Referee' and the source.
The images or other third party material in this Peer Review File are included in the article's Creative Commons license, unless indicated otherwise in a credit line to the material. If material is not included in the article's Creative Commons license and your intended use is not permitted by statutory regulation or exceeds the permitted use, you will need to obtain permission directly from the copyright holder.
To view a copy of this license, visit https://creativecommons.org/licenses/by/4.0/

REVIEWER COMMENTS

Reviewer #1 (Remarks to the Author):

Methods

1. Page 13, line 345. Presenting associations between plasma biomarkers and formal cognitive and functional scales would help fill an important data gap in underrepresented groups. At least include comparisons of plasma biomarker levels by CDR global score and correlations between biomarker levels and CDR sum of boxes, FAQ, and CERAD scores.

We appreciate the reviewer's suggestion. We investigated the links between plasma biomarker levels and cognitive and functional assessments, including CERAD EVOC, FAQ, CDR global score, and CDR sum of boxes in the Cog-Aging-Study–Brazil cohort, and FAQ and CDR global score in the GeNED.ar–Argentina cohort, where CERAD scores and CDR sum of boxes were unavailable. These analyses are now included in the Results sections *"Plasma biomarkers in Cog-Aging-Study–Brazil"* and *"Plasma biomarkers in GeNED.ar–Argentina,"* as well as in Supplementary Figures 6 and 12 as follows:

Page 8:

> *"Higher biomarker levels were associated with lower CERAD EVOC scores, indicating worse episodic memory performance, with larger effect sizes for pTau217, pTau217/Aβ42, and GFAP (Supplementary Fig. 6A-D). Similarly, higher biomarker levels were associated with higher FAQ scores, reflecting greater functional impairment, with more pronounced effects for pTau217, pTau217/Aβ42, and GFAP (Supplementary Fig. 6E-H). Consistent associations were observed for global disease severity: biomarker levels increased across CDR global score categories, with pTau217, pTau217/Aβ42, and GFAP showing the clearest separation (Supplementary Fig. 6I-L), and were positively associated with CDR sum of boxes, with the strongest effects observed for these same biomarkers (Supplementary Fig. 6M-P)"*

Pages 10-11:

> *"No significant associations were observed between biomarker levels and FAQ scores, likely linked to the high proportion of CU participants (Supplementary Fig. 12A-D). However, biomarker levels increased across CDR global score categories, consistent with greater global disease severity (Supplementary Fig. 12E-H). CERAD EVOC and CDR sum of boxes scores were not available in GeNED.ar–Argentina, precluding analyses equivalent to those performed in Cog-Aging-Study–Brazil"*

2. Page 14, line 360. In the description of blood sample processing also mention room temperature during centrifugation, estimated average needle-to-freezer time, and brand and type (or catalog number) of "low

protein binding tubes" used. This is especially critical for local researchers or other researchers in Latin America looking to replicate the findings.

We appreciate the reviewer's remark. We have added these details in the Methods subsection *"Sample collection"* as follows:

Page 20:

> *"CSF samples (Cog-Aging-Study–Brazil) were collected by lumbar puncture at the L3-4 or L4-5 interspace using sterile technique and 15 mL collection tubes. To minimise circadian variability in biomarker levels, procedures were performed at 10 AM. Samples were centrifuged within 2 hours at 3000 g for 10 minutes at 4°C, aliquoted into low-protein-binding tubes (Sarstedt SafeSeal reaction tube, 1.5 mL, Germany) and stored at -80 °C. Blood was collected in both cohorts in EDTA tubes using sterile technique, and within 2 hours, plasma was separated by centrifugation at 3000 g for 15 minutes at room temperature, aliquoted into low-protein-binding tubes (Cog-Aging-Study–Brazil: Sarstedt SafeSeal reaction tube 1.5 mL, Germany; GeNED.ar: Sigma-Aldrich Eppendorf® DNA LoBind 1.5 mL, USA), and stored at -80 °C."*

3. Page 14, line 373. Describe the performance features of the biomarkers in your lab (at least the overall coefficient of variation for each marker), whether samples were run as duplicates, and whether Simoa operators were blinded to clinical data.

We thank the reviewer for this observation. We have included this information in the Methods subsection *"Biomarker measurement"* as follows:

Pages 20-21:

> *"Plasma Aβ42, NfL, and GFAP were measured using the Neurology 4-plex E Simoa assay kit (Quanterix Billerica, USA), and pTau217 with the ALZpath V2 Simoa assay kit (Quanterix Billerica, USA) on a Simoa HD-X platform at the DZNE Bonn, Germany. Plasma biomarkers were run as singlicates by a researcher blinded to the clinical data. Calibrators were run in duplicates. Repeatability and intermediate precision were assessed using low- and high-concentration quality control samples. In Cog-Aging-Study–Brazil samples, coefficients of variation (%CV) ranged from 6.3-6.9% (repeatability) and 6.0-6.2% (intermediate precision) for pTau217, 2.6-3.9% and 2.0-3.2% for Aβ42, 9.9-13.1% and 6.7-8.0% for NfL, and 6.5-7.0% and 6.5-7.2% for GFAP, 5.7-10.2%, respectively. In GeNED.ar–Argentina samples, %CV ranged from 3.5-5.2% and 6.4-5.2% for pTau217, 1.9-4.1% and 2.8-4.3% for Aβ42, 2.5-6.1% and 4.6-5.7% for NfL, and 2.2-7.3% and 3.1-6.4% for GFAP."*

Results

1. Page 5, line 96. "The same procedure was applied" is not possible, since GENED.ar did not have CSF data. The phrase needs to be reworded. Also the rationale and pros and cons of generating cutoffs based on CSF biomarkers or clinical diagnosis should be more clearly stated.

We agree with the reviewer that the sentence about the analysis framework needed to be rephrased and that the rationale for generating the cut-offs needed to be stated more clearly. We have revised both in the Results subsections *"Participant characteristics"* and *"CSF Aβ42/Aβ40 and pTau181 cut-offs and diagnostic concordance in Cog-Aging-Study–Brazil"* as follows:

Page 5:

> *"Cut-offs for CSF biomarkers were first derived in Cog-Aging-Study–Brazil, and plasma biomarker cut-offs were then derived independently and subsequently validated against CSF-based classifications within the same cohort. In GeNED.ar–Argentina, plasma cut-offs were also derived independently; however, no CSF data were available for validation. We next describe the clinical and demographic characteristics of each cohort"*

Pages 6-7:

> *"To derive cohort-specific CSF cut-offs, we employed two methods and compared their performance. First, we used an unbiased, data-driven approach. We applied Gaussian mixture modelling (GMM) to the full sample with available CSF data (CU, n=28; MCI, n=36; DAT, n=47). By identifying natural groupings in biomarker distributions without relying on predefined clinical labels, this approach reduces potential bias from diagnostic misclassification[7](…) Next, we derived clinically based cut-offs by identifying the CSF values that maximised discrimination using Youden's index from a logistic regression model. To ensure clear separation between groups, only CU and DAT participants were included, as MCI is a heterogeneous and diagnostically uncertain group that could attenuate discrimination. Because these cut-offs rely on predefined clinical labels, they may reflect underlying inaccuracies in diagnostic classification (…) Given the high concordance between methods, GMM-derived cut-offs were selected as the reference approach for all subsequent analyses. Although performance was comparable to clinically derived thresholds, GMM provides a data-driven, label-independent framework that can be consistently applied across CSF and plasma biomarker analyses"*

2. Page 5, line 103. Provide the overall prevalence of APOE e3/e3, APOE e3/e4 and APOE e4/e4 genotypes in each cohort.

We have updated Table 1 by adding the prevalence of each *APOE* genotype in both cohorts.

3. Page 6, line 22. Given the novelty of the biofluid phenotypes in these Latin American populations, there is a unique opportunity to provide elements to close the age, race and sex data gaps relevant to underrepresented groups. This is important for comparison with other populations and replication purposes. These sections should start with a more elementary description of data in both cohorts: 1) Results of test of normality for the biofluid data, 2) Differences in fluid biomarker by sex, 3) Overall and within-diagnostic group relationships between biomarkers and age. 4) Median (±IQR) CSF and biomarker levels by clinical diagnostic groups.

We thank the reviewer for this suggestion. We have added the Results subsections *titled "CSF biomarkers in Cog-Aging-Study–Brazil"*, *"Plasma biomarkers in Cog-Aging-Study–Brazil"*, and *"Plasma biomarkers in GeNED.ar–Argentina"*. Additionally, we included Supplementary Figures 1-2, 4-5, and 10-11, which display the biomarker distributions and their associations with sex, age, and clinical diagnosis as follows:

Page 6:

> *"Given the limited availability of CSF biomarker data from Latin American populations, we first performed descriptive analyses assessing their distributional properties, sex differences, age-related associations, and comparison across clinical diagnostic groups.*
>
> *The distributions of the available CSF Aβ42/Aβ40 ratio and pTau181 in this cohort were assessed by visual inspection of histograms and Q-Q plots. The Aβ42/Aβ40 ratio was approximately symmetric on the original scale, and log-transformation did not materially alter its distribution (Supplementary Fig. 1A, C); therefore, subsequent analyses were performed using untransformed values. In contrast, pTau181 was right-skewed on the original scale, and log-transformation substantially reduced the skewness, yielding an approximately symmetric distribution suitable for subsequent analyses (Supplementary Fig. 1B, D).*
>
> *No significant associations were observed between either biomarker and sex (Supplementary Fig. 2A-B) or with age, either in the overall sample or within clinical diagnostic groups (CU, MCI, DAT) (Supplementary Fig. 2C-D). In contrast, both biomarkers were significantly associated with clinical diagnosis, with lower Aβ42/Aβ40 ratio and higher pTau181 levels observed in more advanced disease stages (Supplementary Fig. 2E-F)"*

Page 7:

> *"We next extended the same descriptive framework used for CSF biomarkers to plasma biomarkers, assessing distributional properties, sex differences, age-related associations, and differences across clinical diagnostic groups. Given the greater accessibility of plasma biomarkers for clinical use compared to CSF, we further evaluated their associations with cognitive and functional scales. We focused on pTau217 and pTau217/Aβ42, proposed biomarkers of Aβ pathology, as well as NfL and GFAP, which reflect more general neurodegenerative and astroglial processes involved in AD pathophysiology.*
>
> *All plasma biomarkers exhibited right-skewed distributions on the original scale, which were subsequently log-transformed, yielding approximately symmetric distributions suitable for subsequent analyses (Supplementary Fig. 4). Only GFAP showed a significant association with sex, with higher levels in females ($p=3.41\times10^{-3}$; Supplementary Fig. 5A-D). In the overall sample, pTau217, NfL, and GFAP were significantly correlated with age (pTau217: $r=0.17$, $p=2.74\times10^{-3}$; NfL: $r=0.39$, $p=2.11\times10^{-11}$; GFAP: $r=0.32$, $p=1.28\times10^{-8}$), whereas no association was observed for pTau217/Aβ42 ($r=0.07$, $p=0.23$). Within diagnostic groups, pTau217 showed positive associations with age in CU and MCI participants and a negative association in DAT. Similarly, pTau217/Aβ42 showed a negative association with age in DAT, despite the absence of an overall correlation. NfL and GFAP were associated with age only in CU and MCI groups (Supplementary Fig. 5E-H). Regarding clinical diagnosis, all biomarkers showed increasing levels with disease severity, with pTau217 and pTau217/Aβ42 providing the clearest separation between diagnostic groups (Supplementary Fig. 5I-L)"*

Page 10:

"We next applied the same descriptive framework used for plasma biomarkers in Cog-Aging-Study–Brazil to GeNED.ar–Argentina. As in Cog-Aging-Study–Brazil, all plasma biomarkers showed right-skewed distributions on the original scale, which became approximately symmetric after log-transformation (Supplementary Fig. 10). In contrast to Cog-Aging-Study–Brazil, all biomarkers showed higher levels in males (Supplementary Fig. 11A-D). This pattern is likely explained by differences in age distribution (mean age males: 73.2 years; mean age females: 71 years) and APOE-ε4 frequency (males: 20%; females: 13.3%).

In the overall sample, all biomarkers were significantly correlated with age (pTau217: r=0.48, p=4.96×10-9; pTau217/Aβ42: r=0.41, p=1.31×10-6; NfL: r=0.67, p<2.2×10-16; GFAP: r=0.51, p=4.15×10-10), with generally stronger associations than in Cog-Aging-Study–Brazil. Within diagnostic groups, age associations were more heterogeneous, with pTau217 and pTau217/Aβ42 associated with age only in CU participants, NfL with CU and MCI participants, and GFAP with CU and DAT participants (Supplementary Fig. 11E-H). As observed in Cog-Aging-Study–Brazil, all biomarkers increased with disease severity across diagnostic groups (Supplementary Fig. 11I-L)"

4. Page 6, line 22. This is an opportunity to present data in the context of the ATN(I) system. It's understandable to narrow the scope of the study to Abeta and p-tau markers, but since the 4 Neuro-Plex E kit was used for fluid phenotyping, the data on NfL (N) and GFAP (I) should be presented, at least median and IQR by diagnostic group. This is in the spirit of taking advantage of the uniqueness of these cohorts and to allow a richer biofluid characterization of underrepresented groups. For example, because there is no neuropath data, seen rising levels of GFAP with disease severity would support that the cohorts are enriched for typical AD pathology.

We concur with the reviewer that including NfL and GFAP offers a more comprehensive biofluid profile of these underrepresented groups. Consequently, we have added descriptive analyses of both biomarkers in the Results sections *"Plasma biomarkers in Cog-Aging-Study–Brazil"* and *"Plasma biomarkers in GeNED.ar–Argentina*." These analyses cover biomarker distributions as well as their correlations with sex, age, clinical diagnosis, and cognitive scales. The results are shown in Supplementary Figures 4-6 and 10-12 (see also responses to Comments Methods #1 and Results #3).

5. Page 6, line 22. If CSF values in this study are given the important role of being used as determinants for amyloid pathology, their performance should be further described as well. At least provide 1) levels of CSF biomarker by clinical diagnostic group, 2) CSF cutoff values used for amyloid positivity, and 3) correlations between CSF and plasma biomarkers, when available.

We thank the reviewer for the suggestion. The associations between CSF biomarkers and clinical diagnostic groups are now detailed in the Results section (see response to Comment Results #3). The CSF cut-off for amyloid positivity (Aβ42/Aβ40 ratio: 0.12) was already specified in the original submission (page 6). Additionally, we examined links between CSF and plasma biomarkers in the Cog-Aging-Study–Brazil. These analyses are now presented in the Results subsection *"Plasma biomarkers in Cog-Aging-Study–Brazil"* and shown in Supplementary Figure 7 as follows:

Page 8:

> *"Finally, associations between plasma biomarkers and CSF Aβ42/Aβ40 were examined in the subset of participants with paired CSF-plasma samples (n=45) to assess their relationship to amyloid pathology. pTau217, pTau217/Aβ42, and GFAP showed significant negative associations, whereas NfL showed no association. pTau217 and pTau217/Aβ42 exhibited the strongest associations with CSF Aβ42/Aβ40 (Supplementary Fig. 7)"*

Discussion

1. Page 10, line 241. This section should discuss the clinical associations of the plasma biomarkers and their diagnostic performance in contrast to those observed in the White, and non-diverse populations: e.g., Native-American, African, Asian. My gestalt is that the accuracy may be similar to that described in the White population, with amyloid PET or neuropath-defined cohorts. This scope is relevant for two reasons: 1) highlight similarities and discrepancies to guide further clinical tool development and investigations on variables that mediate the diagnostic utility of these markers in different populations, and 2) advance practical parameters to implement precision medicine when clinicians encounter diverse populations (e.g., Can results of an AD plasma biomarker in Brazilian or Argentinian people living in the US or Europe be equally regarded and interpreted?).

We appreciate the reviewer's valuable suggestion. Our goal was not to make direct comparisons of diagnostic performance between ethnic groups but to examine plasma pTau217 performance in two underrepresented and genetically admixed South American cohorts. However, we agree that contextualizing our results within diverse populations is vital for understanding the broader relevance of these biomarkers. Therefore, we have added to the Discussion a comparison of our findings with previous studies involving underrepresented populations, including cohorts from East and South Asia, Native American-enriched groups, as well as predominantly White cohorts from North America and Europe.

Pages 16-17:

> *"Importantly, the diagnostic performance seen in these South American cohorts was also largely consistent with findings from other underrepresented populations, including cohorts from East and South Asia and Native American-enriched groups. In these populations, plasma pTau217 similarly*

demonstrated high agreement with clinically diagnosed DAT22–27. When considering the comparable performance to predominantly White cohorts from North America and Europe, these results support the potential of pTau217-based biomarkers to be applicable across diverse populations, especially within a two-cut-off model. However, differences in genetic background, comorbidities, socioeconomic factors, and preanalytical conditions may still affect biomarker levels and optimal threshold definition. This underscores the need for ongoing validation in heterogeneous populations as plasma biomarkers move toward routine clinical implementation"

2. Page 11, line 298. The study of the effect of African ancestry is important. A unique opportunity in this cohort, however, is the study of the particular effects of local genetic backgrounds (e.g., pardo, mestizo, indigenous) on the clinical associations of the biomarkers. If not pursued, this should be at least acknowledged as an area of opportunity.

We thank the reviewer for the suggestion. In this study, we specifically examined the interaction between global African ancestry and *APOE-ε4*, as prior research indicated this relationship influences amyloid-related outcomes, making it a pre-specified, hypothesis-driven analysis. We concur that the genetic diversity within these populations offers valuable opportunities for future research into other ancestry components beyond African ancestry and their possible impact on biomarker-phenotype links. We have now acknowledged this in the Discussion section as follows:

Page 18:

> *"Together, these results indicate that ancestry-related effects are complex and involve more than just a single global component, encouraging future studies examining how different combinations of genetic ancestry, beyond global African ancestry alone, may impact biomarker levels and their clinical implications in underrepresented populations"*

3. Page 12, line 321. Other limitations to acknowledge: 1. CSF is not a perfect gold-standard. Studies on path-confirmed cases are still needed in diverse populations. 2) Both cohorts recruit through single centers and the racial and ethnic heterogeneity within a given Latin American country can still be missed. 3) Simoa is not an optimal analytical platform for AD fluid biomarkers. In head-to-head studies that compared either p-tau217 or Ab42/Ab40 measured with different analytical platforms, IPMS was significantly superior than Simoa. ECL also has better performance than Simoa for p-tau217. The performance for Ab with Simoa is among the worst to identify amyloid pathology. The use of Simoa to conduct the analyses should also be mentioned in the abstract.

We thank the reviewer for highlighting these important limitations. Accordingly, we have added in the abstract that we used Simoa.

Page 3:

> *"Using data-driven mixture modelling with single- and two-cut-off approaches on Simoa-based plasma measurements, we assessed concordance with clinical diagnosis (…)"*

Furthermore, we have acknowledged the limitations in the Discussion section.

Pages 18-19:

> *"Limitations include the small number of participants with paired CSF and plasma samples in Cog-Aging-Study–Brazil, the lack of CSF samples in GeNED.ar–Argentina, which limited direct comparison of plasma biomarker performance between cohorts, and the use of global rather than local ancestry to assess the impact of APOE-ε4 on plasma biomarkers, which might underestimate the effect of ancestry-specific modulation of APOE-ε4-related AD risk. Additionally, both cohorts were recruited from single centers, which may not fully represent the genetic diversity within each country. Although CSF was used as the reference standard, validation against neuropathologically confirmed cases across diverse populations is still necessary. Moreover, biomarker measurements can vary depending on the analytical platform used. While Simoa offers high sensitivity, differences among assay technologies should be considered when comparing absolute values or cut-offs across studies. Lastly, since detailed medication data were not consistently available, we could not separate the effects of comorbidities from their pharmacological treatments, and the observed associations might reflect their combined impact of both"*

Reviewer #2 (Remarks to the Author):

This study evaluates plasma pTau217 (and pTau217/Aβ42) as biomarkers for Alzheimer's disease in two cohorts from Brazil and Argentina, addressing the lack of data from low and middle income countries. Using data-driven cut-offs (GMM), the authors show good concordance of these plasma biomarkers with clinical AD diagnosis and, in a subgroup of the Brazilian cohort, with CSF status. Demographic and clinical factors (APOE ε4, BMI, eGFR, education) influenced biomarker levels. African ancestry modified APOE effects on CSF.
The main strength and novelty of this study is including genetically admixed South American populations, and two independent cohorts. I particularly like the findings on the effect of ancestry in blood biomarkers.

There are some limitations that need to be addressed:

1. A main limitation of the study is that only a subgroup of the Brazilian cohort had CSF data available, and some of these were not temporally matched with plasma collection, limiting validation against a biological reference standard. Consequently, concordance is largely based on clinical diagnosis. In addition, the absence of CSF or PET data in the Argentinian cohort further restricts conclusions about diagnostic accuracy in that population.

We appreciate the reviewer highlighting these significant limitations. We recognize that only a subset of Cog-Aging-Study–Brazil included paired CSF and plasma data, preventing validation against CSF-defined amyloid status across all participants. Additionally, the lack of CSF biomarkers in GeNED.ar–Argentina restricted direct evaluation of diagnostic accuracy against established amyloid markers. These issues were already discussed in the original submission's Discussion section.

The data-driven GMM framework demonstrated strong agreement with both clinical diagnoses and CSF-defined amyloid status in Cog-Aging-Study–Brazil. Similarly, GeNED.ar–Argentina, despite not having CSF biomarkers, showed comparable socioeconomic, demographic, and clinical features, along with similar concordance with clinical diagnosis. These results collectively endorse the potential use of this framework in underrepresented and resource-limited groups where gold-standard biomarkers are often unavailable.

2. There are multiple differences between participants with plasma only, CSf only, and those with both plasma and CSF in the Brazilian cohort, raising concerns about potential selection bias. The criteria for CSF availability and the representativeness of this subgroup should be clarified.

We thank the reviewer for highlighting this important point. The differences among participants with plasma-only, CSF-only, and paired CSF-plasma samples in Cog-Aging-Study–Brazil are primarily due to differences in recruitment pathways and ethical constraints related to CSF collection.

Participants with plasma samples were primarily recruited via our outpatient cognitive evaluation centre. The subgroup with available CSF samples also included individuals recruited from patients already scheduled for procedures requiring spinal anaesthesia. Since lumbar puncture is invasive and ethically unjustifiable solely for research in asymptomatic, cognitively unimpaired people, recruitment through surgical settings enabled the inclusion of cognitively unimpaired participants with CSF data without subjecting them to additional invasive procedures for research. As a result, the CSF subgroup exhibited differences from the overall cohort in several clinical and demographic factors, including the distribution of diagnostic groups.

Importantly, regardless of recruitment source, all participants completed the same standardized clinical, neurological, and cognitive assessments conducted by the same research team. For those recruited via the surgical pathway, assessments took place one to three months post-surgery to reduce effects of transient postoperative or anaesthesia-related cognitive changes. This information is included in the Supplementary Methods as follows:

Page 2:

> *"Differences in sample composition across these groups reflect distinct recruitment and sampling pathways. In particular, CSF collection was performed both in participants recruited through the*

cognitive outpatient clinic and in individuals undergoing procedures requiring spinal anaesthesia, whereas plasma samples were obtained more broadly within the cohort. Consequently, availability of CSF and paired samples was influenced by recruitment pathway, clinical indication, and logistical/ethical constraints"

3. The rationale for using Youden's index derived from logistic regression models including only CU and AD dementia participants is unclear.

We thank the reviewer for this comment. We have rewritten the rationale in the Results subsection *"CSF Aβ42/Aβ40 and pTau181 cut-offs and diagnostic concordance in Cog-Aging-Study–Brazil"* as follows:

Pages 6-7:

> *"Next, we derived clinically based cut-offs by identifying the CSF values that maximised discrimination using Youden's index from a logistic regression model. To ensure clear separation between groups, only CU and DAT participants were included, as MCI is a heterogeneous and diagnostically uncertain group that could attenuate discrimination. Because these cut-offs rely on predefined clinical labels, they may reflect underlying inaccuracies in diagnostic classification"*

4. It is also unclear why plasma biomarker cut-offs were primarily derived using a data-driven Gaussian mixture modelling approach rather than being directly calculated against the available CSF reference standard.

We appreciate the reviewer's important comment. Our main reason for choosing the data-driven GMM approach was to create a framework applicable across different cohorts, including those lacking gold-standard biomarkers like CSF or PET. This was especially relevant for GeNED.ar–Argentina, where CSF biomarkers were not available, highlighting a common issue in underrepresented and resource-limited cohorts in the region. To clarify this point, we revised the Results subsection *"CSF Aβ42/Aβ40 and pTau181 cut-offs and diagnostic concordance in Cog-Aging-Study–Brazil"* to better justify why GMM-derived cut-offs were selected as follows:

Page 7:

> *"Given the high concordance between methods, GMM-derived cut-offs were selected as the reference approach for all subsequent analyses. Although performance was comparable to clinically derived thresholds, GMM provides a data-driven, label-independent framework that can be consistently applied across CSF and plasma biomarker analyses"*

Importantly, we are not proposing this framework de novo. Recent work by TM Liew[1], also cited in the original submission, proposed using GMM-based approaches for biomarker cut-off estimation in cohorts without gold-standard reference measures.

5. The finding that the pTau217/Aβ42 ratio shows a slightly larger grey zone than pTau217 alone contrasts with previous studies and warrants further discussion. May this may be partly related to the fact that the

ratio combines measurements obtained from different analytical assays (Alzpath for pTau217 and Quanterix N4PE assay for Aβ42)? which should be explicitly acknowledged as a limitation.

We concur with the reviewer that the larger grey zone for pTau217/Aβ42 versus pTau217 alone merits additional discussion. We think this difference may stem from Aβ42 being measured via the Simoa platform, which generally performs worse than mass spectrometry-based methods. Additionally, Aβ42 levels are highly sensitive to preanalytical variability. We have updated the Discussion to reflect these points as follows:

Page 16:

> *"Conversely, the finding that pTau217/Aβ42 produced a larger grey zone contrasts with recent reports showing that pTau217/Aβ42 reduced the proportion of indeterminate cases despite not improving diagnostic accuracy[19,20]. One possible explanation is methodological. In both studies, plasma Aβ42 was measured using mass spectrometry-based approaches, which have shown superior discriminative performance for amyloid pathology defined by CSF Aβ42/Aβ40 compared with immunoassay-based methods. In our study, both pTau217 and Aβ42 were measured on the Simoa platform, which may have contributed to the slightly larger grey zone observed for pTau217/Aβ42. In addition, plasma Aβ42 and Aβ40 have been reported to be among the plasma biomarkers most sensitive to preanalytical variability, showing decreases greater than 10% under storage or centrifugation delays21, which may have further influenced the performance of the ratio. These observations also raise the broader question of how plasma cut-offs should be defined and applied across populations, as the platform used and preanalytical factors may differ from those of the populations in which current thresholds were originally derived"*

6. Finally, when evaluating the impact of comorbidities such as hypertension, dyslipidemia, and diabetes on plasma biomarkers, it is unclear how the authors disentangle effects of the conditions themselves from those of their treatments. This limitation should be discussed, and medication use considered where possible.

We thank the reviewer for pointing out this limitation. Accordingly, we have discussed it in the Discussion section as follows:

Page 19:

> *"Lastly, since detailed medication data were not consistently available, we could not separate the effects of comorbidities from their pharmacological treatments, and the observed associations might reflect their combined impact of both"*

Reviewer #3 (Remarks to the Author):

This is an important study seeking to evaluate plasma ptau217 and its ratio with AB42 in two Latin American cohorts. These works are urgently needed in helping to democratize blood biomarker use worldwide. The work needs quite a few necessary improvements to get to the level needed for this journal.

I have a few comments.

- It is unclear to what end the cutpoint estimation serves. Instead, I suggest using published cutpoints for the assays used and examine their transferability.
- - Comment on why or why not cutpoints from elsewhere may or may not be transferable to yours. Include analytical and clinical reasoning.

We appreciate the reviewer's valuable comments. Our reason for establishing cohort-specific cut-offs is that biomarker threshold transferability across different populations cannot be taken for granted, especially in underrepresented groups with unique demographic, socioeconomic, and genetic traits compared to populations where most plasma biomarker cut-offs were originally determined. Therefore, we viewed local cut-off estimation as a crucial initial step to assess whether existing thresholds could be applicable to South American cohorts. Following the reviewer's suggestion, we further assessed the performance of externally published pTau217 cut-offs, derived using the same analysis platform and assay conditions[2]. These analyses are now included in the Results sections *titled "Data driven plasma pTau217 and pTau217/Aβ42 cut-offs in Cog-Aging-Study–Brazil"* and *"Data driven plasma pTau217 and pTau217/Aβ42 cut-offs in GeNED.ar–Argentina*," as well as in Table 2 and Figures 2-3 as follows:

Pages 9-10:

> *"Finally, we compared the performance of cohort-specific cut-offs with externally published thresholds. Because biomarker values depend on the analytical platform, we compared only studies using the same equipment (Simoa) and assay (Alzpath for pTau217, Neurology 4-plex for Aβ42). Ashton et al.4 derived pTau217 cut-offs in the same conditions in cohorts from North America and Europe. Using their published single cut-off (0.42 pg/mL), 50% of participants were classified as pTau217+, a higher proportion than with the cohort-specific threshold. Concordance with clinical diagnosis remained high; however, a substantially larger proportion of MCI participants were classified as pTau217+ (48.9% vs 27.3% with the cohort-specific cut-off; Fig. 2C). In contrast, the two-cut-off approach (lower cut-off: 0.40 pg/mL; upper cut-off: 0.63 pg/mL) yielded distributions more comparable to those obtained with cohort-specific cut-offs, with 47% of participants classified as pTau217-, 34.9% as pTau217+, and 18.1% as indeterminate. The MCI group showed the highest proportion of indeterminate cases (25.2%; Fig. 2D), primarily reflecting a reclassification of participants previously categorised as pTau217+ under the single-threshold approach, while the proportion classified as pTau217- remained largely unchanged. In the subset of 45 participants with paired CSF samples, both approaches showed similar overall performance, although the published cut-offs yielded slightly higher NPV and sensitivity at the expense of PPV and specificity (Table 2). No external cut-offs for pTau217/Aβ42 using comparable analytical conditions were available, precluding a similar comparison for this biomarker"*

Page 11-12:

> *"When applying the published pTau217 cut-offs from Ashton et al.4, we observed a classification pattern broadly comparable to that seen in Cog-Aging-Study–Brazil. The single-threshold approach*

classified a higher proportion of participants as pTau217+ (36.7%) than the cohort-specific threshold, while maintaining high concordance with clinical diagnosis. This effect was particularly evident in the MCI group, where a substantially larger proportion was classified as pTau217+ (42.9% vs 25.7% using the cohort-specific cut-off; Fig. 3C). By contrast, the two-cut-off strategy resulted in lower proportions of participants classified as pTau217- (58.2%) and pTau217+ (22.4%), with a corresponding increase in indeterminate cases (19.4%) compared with the cohort-specific approach. As in Cog-Aging-Study–Brazil, the increased indeterminate classifications in MCI participants (20.0%; Fig. 3D) primarily reflected reclassification of individuals previously considered pTau217+ under the single-threshold strategy (42.9% vs 25.7%), whereas the proportion classified as pTau217- remained relatively stable"

Overall, the published cut-offs closely aligned with clinical diagnoses and performed comparably to the cohort-specific thresholds. However, they classified more participants, especially those with MCI, as pTau217+. No published external cut-offs for pTau217/Aβ42 under similar analytical conditions were available, which prevented similar analyses for this biomarker.

These findings indicate that published pTau217 thresholds could be broadly applicable across different populations if measured consistently, while also emphasizing the importance of assessing cohorts individually, especially in populations underrepresented in biomarker studies.

- The authors should use the plasma-CSF paired samples to a better advantage than they do currently. The main focus of the work should be on plasma. However, in subanalyses, show how well correlated the plasma and CSF set are.

We appreciate the reviewer's suggestion. This study mainly focused on assessing plasma pTau217 and the pTau217/Aβ42 ratio as accessible biomarkers in underrepresented South American populations. Meanwhile, CSF biomarkers served mainly as reference measures for validation, using a subset of participants from Cog-Aging-Study–Brazil who had paired CSF and plasma samples.

In response to the reviewer's suggestion, we also examined the relationships between CSF and plasma biomarkers in the same group of participants with paired CSF-plasma samples from Cog-Aging-Study–Brazil. These analyses are now part of the Results section *titled "Plasma biomarkers in Cog-Aging-Study–Brazil"* and are shown in Supplementary Figure 7 as follows:

Page 8:

"Finally, associations between plasma biomarkers and CSF Aβ42/Aβ40 were examined in the subset of participants with paired CSF-plasma samples (n=45) to assess their relationship to amyloid pathology. pTau217, pTau217/Aβ42, and GFAP showed significant negative associations, whereas NfL showed no association. pTau217 and pTau217/Aβ42 exhibited the strongest associations with CSF Aβ42/Aβ40 (Supplementary Fig. 7)"

- In Table 1, focus on only plasma and have a separate table for characteristics of CSF-plasma pairs.

We appreciate the reviewer's suggestion. Our goal with Table 1 was to offer a detailed overview of the various participant groups involved in each analysis, especially in the Cog-Aging-Study–Brazil, where CSF and plasma availability varied significantly among participants and collection timepoints. We think that displaying all groups in one table makes it easier to compare demographic and clinical features across the different analysed populations and enhances transparency of the cohort makeup. To make the table clearer, we updated it by adding aggregated "All CSF" and "All plasma" columns that summarise all participants contributing to CSF- and plasma-based analyses, respectively.

- Use pooled samples from both cohorts to increase sample size e.g. in cutpoint estimation analyses.

We thank the reviewer for this suggestion. In response, we conducted additional sensitivity analyses using pooled data from the Cog-Aging-Study–Brazil and GeNED.ar–Argentina to evaluate whether the GMM-derived cut-off estimates remained stable when based on a larger combined sample. These analyses are now included in the Results subsection *"Stability of pTau217 cut-off estimation in pooled South American cohorts"*, in Supplementary Tables 4-5, and in Supplementary Figure 15 as follows:

Pages 11-12:

> *"To assess the stability of pTau217 cut-off estimates with an increased sample size, we conducted complementary pooled analyses combining participants from Cog-Aging-Study–Brazil and GeNED.ar–Argentina. Because cohort-specific analyses yielded highly comparable cut-off values, pooled analyses were used to evaluate whether threshold estimates remained consistent when derived from a larger combined sample.*
>
> *We applied the same GMM framework to all participants with available plasma samples (CU, n=173; MCI, n=174; DAT, n=91). As in the individual cohort analysis, a bimodal distribution with equal variances provided the best fit (Supplementary Table 4). Notably, pooled analyses yielded the same single cut-offs obtained independently in both cohorts: 0.63 pg/mL for pTau217 (OVL coefficient, 0.13) and 0.12 for pTau217/Aβ42 (OVL coefficient, 0.18). Using these thresholds, 31.1% participants were classified as pTau217+ (Supplementary Fig. 15A), and 31.6% as pTau217/Aβ42+ (Supplementary Fig. 15B). Similar to the cohort-specific analyses, biomarker classifications showed high concordance with clinical diagnosis, with at least 83% of CU participants classified within the normal distribution and at least 67% of DAT classified within the abnormal distribution.*
>
> *We next evaluated the two-cut-off approach using probability thresholds of ≥0.80, ≥0.85, or ≥0.90. For pTau217, lower and upper cut-off values were highly similar to those obtained in the individual cohorts. Classification distributions were stable across thresholds, with only modest variation in category proportions. The proportion classified as pTau217+ remained relatively stable across thresholds (30.3% to 26.6%), as did the proportion classified as pTau217- (57.6% to 53.6%), while indeterminate classifications gradually increased from 12.1% to 19.7%, particularly at the ≥0.90 threshold*

(Supplementary Fig. 15C, E, G). Concordance with clinical diagnosis remained high, with approximately 80% of CU participants classified as pTau217- and 60-70% of DAT participants as pTau217+. Indeterminate classifications were evenly distributed across diagnostic groups.

For pTau217/Aβ42, the lower and upper cut-offs were also highly comparable to those obtained in the individual cohorts. Across probability thresholds, classification distributions were stable, with only modest variation in category proportions. The proportion classified as pTau217/Aβ42+ showed minimal variation across thresholds (28.7% to 24.9%), as did the proportion classified as pTau217/Aβ42- (74.8% to 71.3%), while indeterminate classifications increased gradually from 22.9% to 30.2%, particularly at the ≥0.90 threshold (Supplementary Fig. 15D, F, H). Concordance with clinical diagnosis was also high, though consistently lower than for pTau217 alone. Approximately 75% of CU participants were classified as pTau217/Aβ42-, whereas around 50% of DAT participants were classified as pTau217/Aβ42+.

Finally, to facilitate comparison with the cohort-specific analyses, we also evaluated plasma cut-off performance in the subset of participants with paired CSF samples from Cog-Aging-Study–Brazil (A-, n=17; A+, n=28). For pTau217, the two-cut-off model at the ≥0.85 probability threshold achieved the best overall performance, yielding a PPV of 0.88 and an NPV of 0.81. For pTau217/Aβ42, the model using the ≥0.80 probability threshold showed the best balance between PPV (0.90) and NPV (0.69), whereas the ≥0.90 threshold provided slightly higher NPV at the expense of a larger indeterminate category (Supplementary Table 5). Overall, these findings were consistent with the cohort-specific analyses, supporting the stability of GMM-derived plasma cut-offs when estimated in a larger pooled sample."

Overall, pooled analyses yielded cut-off estimates identical to those from the individual cohorts, and classification patterns remained highly consistent across diagnostic groups and probability thresholds.

- If you have clinical follow-up information, use that to evalaute the biomarker and cutpoint performance.

We agree that longitudinal clinical follow-up would provide additional information on biomarker and cut-off performance over time. However, longitudinal follow-up data were not available for the present analyses in either cohort.

References

1. Liew, T. M. A modelling approach to derive population-specific cutoff for plasma p-Tau217. *J. Prev. Alzheimer's Dis.* **12**, 100264 (2025).
2. Ashton, N. J. *et al.* Diagnostic Accuracy of a Plasma Phosphorylated Tau 217 Immunoassay for Alzheimer Disease Pathology. *JAMA Neurol.* **81**, 255 (2024).
